# Macrocyclic Aromatic Oligoamides with Diphenyladamantane Units: Conformational Change from Folded to Open by N-Alkyl Removal

**DOI:** 10.3390/molecules30214185

**Published:** 2025-10-26

**Authors:** Sae Maeda, Ryota Usami, Kei Takamatsu, Moemi Takato, Shoko Kikkawa, Hidemasa Hikawa, Isao Azumaya

**Affiliations:** Faculty of Pharmaceutical Sciences, Toho University, 2-2-1 Miyama, Funabashi 274-8510, Chiba, Japan

**Keywords:** macrocyclic aromatic amides, cyclic oligomer, folded structure, open structure

## Abstract

Macrocyclic aromatic amide oligomers of various sizes containing tertiary amides and diphenyladamantane moieties were synthesized by condensation reactions using PhP_3_Cl_2_ or LiHMDS. The corresponding oligomers with secondary amides were obtained by removal of N-decyloxybenzyl groups from the amide nitrogen. X-ray crystal structure analysis showed that the oligomers with tertiary N-ethyl amides adopted folded conformations, consistent with the *cis*-preference of tertiary aromatic amides. In contrast, comparison of ^1^H NMR spectra and conformational analysis by force field calculations indicated that the oligomers with secondary amides adopted open conformations. These results also demonstrated that the synthetic strategy is effective for the preparation of macrocyclic molecules with large inner cavities.

## 1. Introduction

Macrocyclic compounds are widely used as platforms for artificial molecules with various functions [1]. In particular, many macrocyclic compounds with repeating structures have been synthesized so far, depending on the monomer design and choice of condensation reaction [2,3,4,5]. A major application of such macrocyclic compounds is molecular recognition [6]. For effective molecular recognition, it is important that the inside of the ring has an appropriate size and shape for the guest molecule. Moreover, through the incorporation of various functional groups into the inner pores of a macrocycle, the molecules can achieve precise molecular recognition through multipoint interaction.

Aromatic amides have been widely used as part of the repeating units in the synthesis of macrocyclic compounds [7,8]. We previously reported on the conformational properties of aromatic amides: aromatic secondary amides such as benzanilide exist in *trans* conformation both in the crystal and in solution, while the N-methylated derivative exists in *cis* conformation [9]. Utilizing this stereochemical property of aromatic amides, we synthesized macrocyclic aromatic amides linked by tertiary amides, which produced cavities of various sizes and shapes [10,11]. Furthermore, we demonstrated that a macrocycle composed of alternating secondary and tertiary amide bonds was synthesized by dealkylation of a cyclic oligomer having chemically removable alkyl groups, and that the resulting cyclic structure with a triangular-shaped large cavity due to alternating *cis* and *trans* conformations of the amide moieties [12,13].

**Figure 1:**
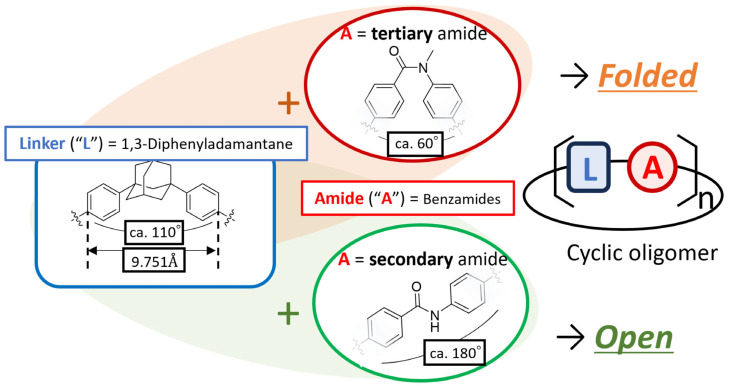
Outline of this paper.

In this study, we designed and synthesized cyclic aromatic oligoamides containing the 1,3-diphenyladamantane skeleton as novel macrocyclic compounds with cavities of specific size and shape (Figure 1). First, derivatives of the macrocyclic compounds bearing tertiary amide groups, in which ethyl groups were introduced as substituents on the amide nitrogens, were synthesized and subjected to X-ray crystal structure analysis. Next, the macrocyclic compounds containing secondary amides were synthesized by first preparing cyclic compounds bearing N-decyloxybenzyl groups as substituents on the amide nitrogens, followed by removal of these substituents. The structures of the secondary amide derivatives were estimated from ^1^H NMR measurements in solution and conformational analysis using force field calculations, and then compared with those bearing tertiary amides. The bonding direction at the *para* position of the two phenyl groups of 1,3-diphenyladamantane corresponds to the bond angle of the sp^3^ hybridization orbital, and the spatial distance is about 9.75 Å according to the crystal structure [14]. The adamantane substructure is hydrophobic [15,16,17] and can impart lipophilicity even to compounds bearing highly polar substituents, such as aromatic secondary amides, which otherwise reduce solubility in organic solvents. This property is a distinctive feature of the adamantane unit.

## 2. Results and Discussion

### 2.1. Synthesis of Macrocyclic Compounds Comprising Tertiary Amide Moieties

#### 2.1.1. Synthesis of a Monomer

**Scheme 1:**
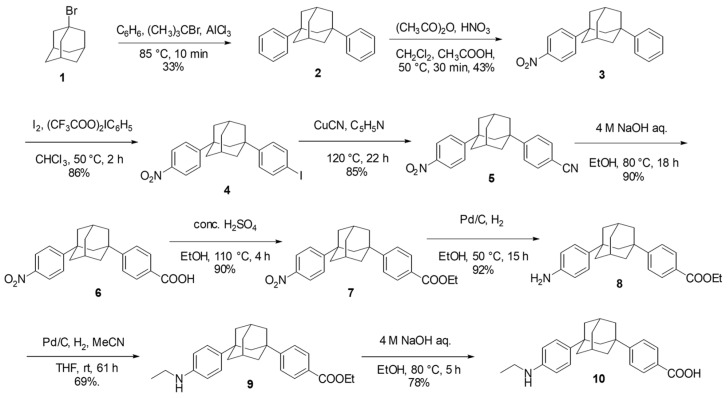
A synthetic route of monomer **10** (N-ethyl derivative).

A monomer for the synthesis of the cyclic amide bearing diphenyladamantane moieties was synthesized according to Scheme 1. First, 1,3-diphenyladamantane (**2**) was obtained by Friedel-Crafts type arylation of 1-bromoadamantane (**1**) [18,19]. Compound **2** was mononitrated with a mixed anhydride of acetic acid and nitric acid [20], followed by iodination using iodine with di(trifluoroacetoxy)iodobenzene [21] to give compound **4**. The iodo group was then substituted by a cyano group by a Rosenmund–von Braun reaction using copper cyanide [22], and the resulting nitrile was hydrolyzed to give compound **6**. After esterification of the carboxy group, the nitro group was reduced to an amino group by catalytic hydrogenation using Pd/C [23]. The subsequent reductive amination [24] was carried out in the presence of acetonitrile in tetrahydrofuran (THF) to give compound **9,** which has an ethyl group on the amino nitrogen. Finally, compound **9** was hydrolyzed to give compound **10**.

#### 2.1.2. Cyclization of the Monomer and Isolation of Cyclic Oligomers

**Scheme 2:**
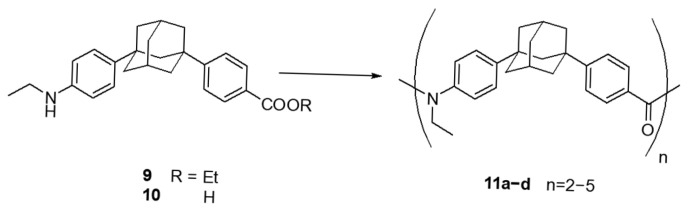
Synthesis of cyclic dimer (**11a**), cyclic trimer (**11b**), cyclic tetramer (**11c**) and cyclic pentamer (**11d**) from compounds **9** and **10**.

**Figure 2:**
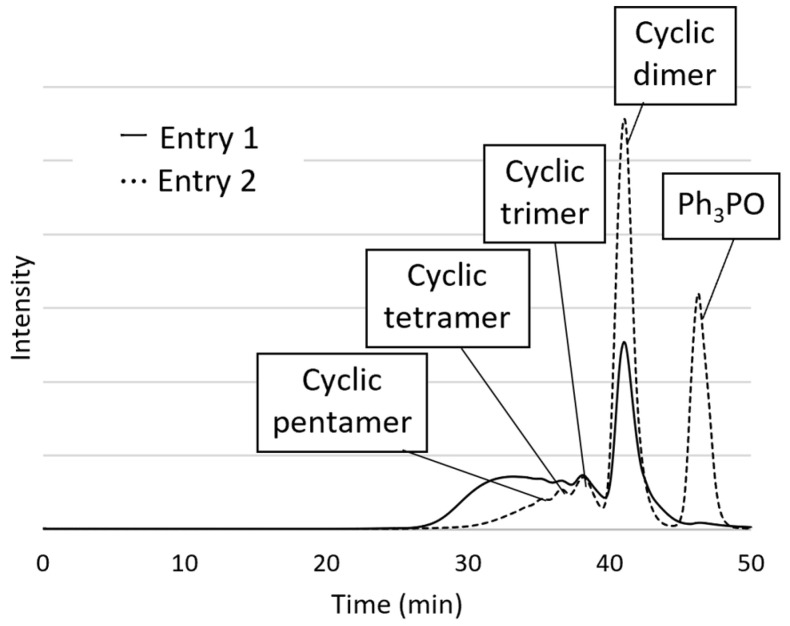
GPC profiles of the crude products obtained in the reactions: LiHMDS in THF (Entry 1, solid line) and Ph_3_PCl_2_ in (CHCl_2_)_2_ (Entry 2, dashed line).

Cyclization reactions (Scheme 2) of the monomers were carried out under two different reaction conditions (Table 1): (i) using lithium bis(trimethylsilyl)amide (LiHMDS) in THF with the ester **9** (Entry 1) [25,26] and (ii) using Ph_3_PCl_2_ in (CHCl_2_)_2_ with the carboxylic acid **10** (Entry 2). The distributions of the resulting cyclic oligomers were analyzed by gel permeation chromatography (GPC), and the GPC profiles of the crude products are shown in Figure 2.

Cyclic dimers were the most abundant products under both reaction conditions. Under the condition using LiHMDS (Entry 1), the total yield of cyclic products (n = 2–5, **11a**–**d**) was 54%. In the GPC profile, a broad peak appeared in the high molecular weight region above the pentamer. ^1^H NMR analysis of this fraction showed a signal at high field in the aromatic region (around 6.8 ppm), indicating the presence of chain oligomers. In contrast, under the condition using Ph_3_PCl_2_ (Entry 2), the total yield of cyclic products (n = 2–5, **11a**–**d**) was 90%, and only a small proportion (<10%) of further cyclized or chain oligomers was obtained. Comparing the two reactions, the reaction using LiHMDS was favorable for the formation of larger cyclic products beyond the trimer (**11b**–**d**), since it could be conducted at room temperature and produced few byproducts other than those derived from the starting material. In addition, the ester intermediate could be directly employed as the starting material. On the other hand, the reaction using Ph_3_PCl_2_ was more effective for obtaining cyclic dimer (**11a**), although it required high temperature and generated Ph_3_PO as a byproduct in large amounts to be removed.

### 2.2. X-Ray Crystal Structure Analysis

**Figure 3:**
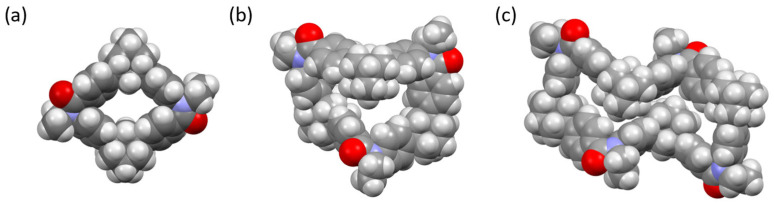
Space-filling representations of the crystal structure of cyclic oligoamide molecules in the unit cell: (**a**) cyclic dimer **11a** (one of the three independent molecules) (CCDC-2486609), (**b**) cyclic trimer **11b** (CCDC-2486610), and (**c**) cyclic tetramer **11c** (CCDC-2486611). Crystal structures are color-coded as follows: C (gray), O (red), N (blue), and H (white). For details of the crystal packing, see the Supporting Information.

Among the isolated cyclic oligoamides, single crystals of cyclic dimer (**11a**), cyclic trimer (**11b**), and cyclic tetramer (**11c**) suitable for X-ray structural analysis were obtained by recrystallization from CHCl_3_/ethyl acetate, CHCl_3_/methanol, and CHCl_3_/acetonitrile, respectively (Figure 3).

**Figure 4:**
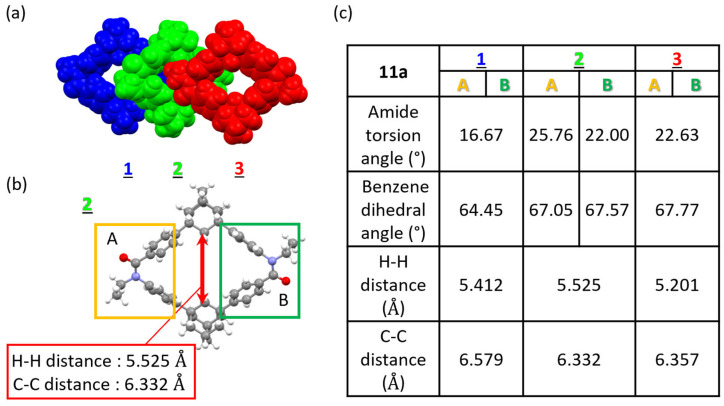
Crystal structure of cyclic dimer **11a**. (**a**) Each molecule was shown in different colors and numbered 1–3. (**b**) Structure of molecule 2, which is color-coded as follows: C (gray), O (red), N (blue), and H (white). (**c**) List of amide torsion angles, benzene ring dihedral angles, and intramolecular distances between hydrogen atoms and between carbon atoms in each molecule.

For the cyclic dimer (**11a**), the crystal contained four crystallographically independent amide units, each corresponding to half of a molecule in the unit cell, resulting in three different conformations (1–3 in Figure 4). Conformation 2 at the center had different torsion angles of the amide bonds and dihedral angles between benzene rings at both ends of the amide bonds, whereas conformations 1 and 3 had the same value for these angles due to the symmetry operations. The molecules in the crystal formed a cavity in the center of the ring structure. The distance between the adamantane carbon across the ring was 6.332 Å, and the hydrogen-to-hydrogen distance was 5.525 Å (Figure 4). The cavity was occupied by the terminal methyl group of the N-ethyl substituent of the adjacent molecule.

**Figure 5:**
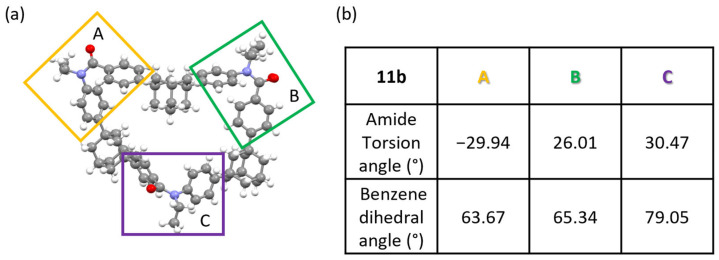
(**a**) Crystal structure of cyclic trimer **11b**. (**b**) List of amide torsion angles and benzene ring dihedral angles at each amide bond site. Crystal structures are color-coded as follows: C (gray), O (red), N (blue), and H (white).

In the crystal structure of the cyclic trimer (**11b**), all the amide groups adopted the *cis* form, and the ring exhibited a folded conformation (Figure 5). The central cavity of the ring structure was occupied by one of the adamantane moieties oriented inward due to molecular folding.

**Figure 6:**
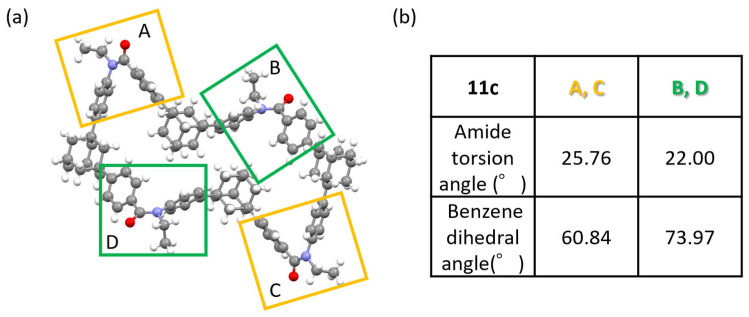
(**a**) Crystal structure of cyclic trimer **11c**. (**b**) List of amide torsion angles and benzene ring dihedral angles at each amide bond site. Crystal structure is color-coded as follows: C (gray), O (red), N (blue), and H (white).

In the crystal structure of the cyclic tetramer (**11c**), the amide groups also adopted *cis* form. The tetrameric ring structure had a center of symmetry, and the central cavity of the ring structure was filled by folding, giving the molecule a chair-shaped conformation (Figure 6).

### 2.3. Synthesis of Cyclic Oligomers Comprising Secondary Amide Moieties

**Scheme 3:**
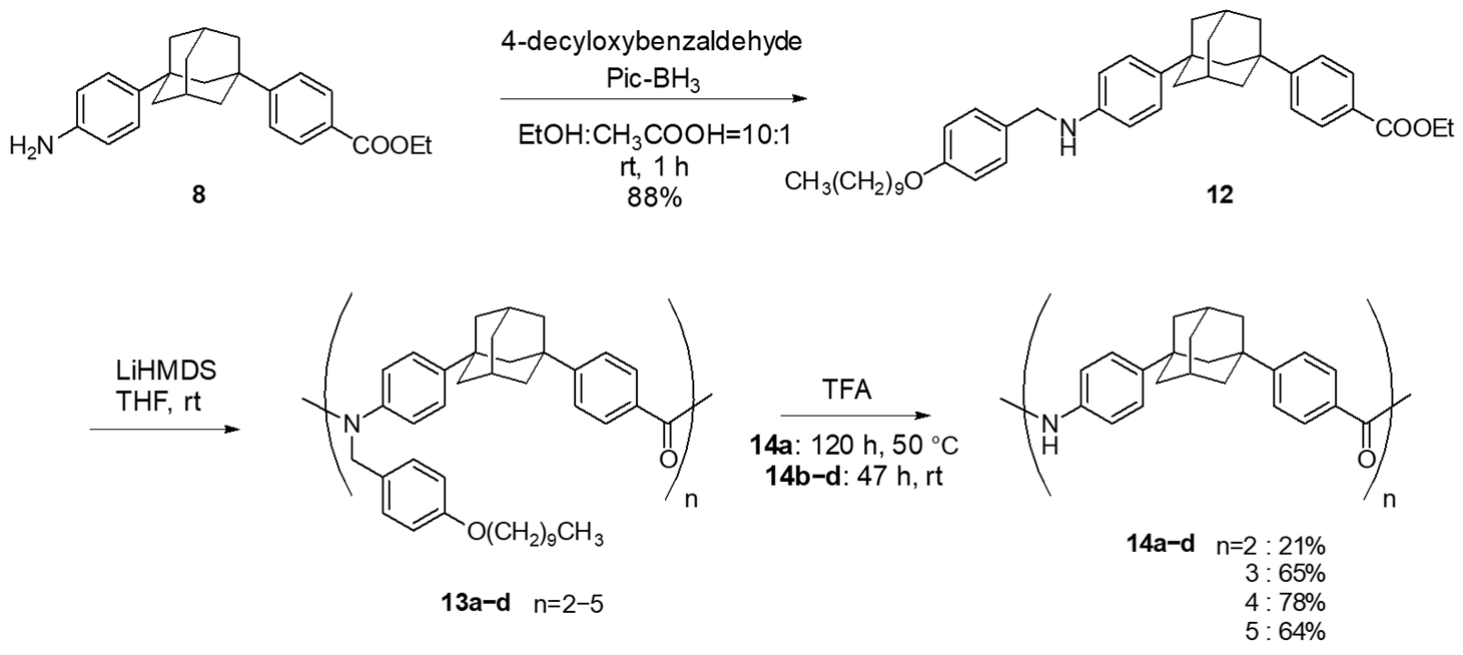
Synthesis of N-decyloxybenzyl derivatives and subsequent dealkylation of the N-decyloxybenzyl groups.

To investigate the structures of the NH analogs of macrocyclic compounds bearing diphenyladamantane moieties, we synthesized N-decyloxybenzyl analogs of **13a**–**13d**. The N-decyloxybenzyl group should be removed under acidic condition such as trifluoroacetic acid (TFA) [27]. The corresponding monomer was prepared from compound **8** (Scheme 3). Reductive amination of **8** with 4-decyloxybenzylaldehyde in the presence of picoline-borane complex as a reducing reagent to afforded the N-decyloxybenzylated compound **12** [28]. Compound **12** was then cyclized under the same conditions for the N-ethyl derivatives (LiHMDS in THF, rt). The cyclic dimer to pentamer (**13a**–**13d**) were obtained by GPC separation of the crude products (Table 2).

Finally, the N-decyloxybenzyl group of cyclized compounds **13a**–**13d** were removed with TFA, affording the corresponding secondary amide derivatives **14a**–**14d**.

### 2.4. Comparison of Preferential Conformations of the Macrocyclic Compounds in Solutions

**Figure 7:**
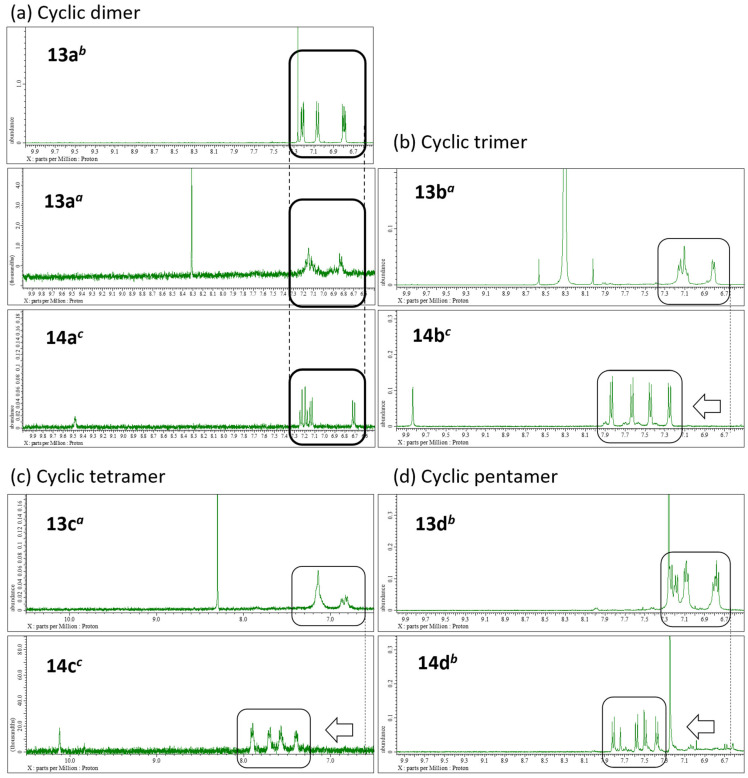
Comparison of ^1^H NMR spectra (the corresponding aromatic protons are indicated within the boxes.) of (**a**) cyclic dimer (**13a** vs. **14a**), (**b**) cyclic trimer (**13b** vs. **14b**), (**c**) cyclic tetramer (**13c** vs. **14c**), (**d**) cyclic pentamer (**13d** vs. **14d**). Solvents: *^a^* DMSO-*d_6_* with 3 drops of CDCl_3_; *^b^* CDCl_3_; *^c^* DMSO-*d_6_*.

^1^H NMR spectra of cyclic oligomers of N-decyloxybenzyl derivatives **13a**–**13d** and corresponding NH derivatives **14a**–**14d** were compared. (i.e., **13a** vs. **14a**, **13b** vs. **14b**, **13c** vs. **14c** and **13d** vs. **14d**). Except for the NH cyclic pentamer **14d**, the NH cyclic compounds **14a**–**14c** were insoluble in CDCl_3_; therefore, their ^1^H NMR spectra were recorded in DMSO-*d*_6_. In contrast, the N-decyloxybenzyl derivatives **13a**–**13c** were insoluble in DMSO-*d*_6_. Thus, for comparison of the chemical shift values with those of the NH compounds **14a**–**14c**, measurements were conducted in a mixed solvent of DMSO-*d*_6_ containing a small amount of CDCl_3_. As shown in Figure 7a, the chemical shift values of cyclic dimer **13a** measured in CDCl_3_ and in the mixed DMSO-*d*_6_/CDCl_3_ solvent were nearly identical. Therefore, the same method was applied to cyclic trimer **13b** and cyclic tetramer **13c**, and the resulting data obtained were used for comparison.

Comparison of the ^1^H NMR spectra of each ring size of cyclic oligomers showed significant differences in the chemical shifts between **13b** (N-decyloxybenzyl cyclic trimer) and **14b** (NH cyclic trimer) (Figure 7b), between **13c** and **14c** (Figure 7c), and between **13d** and **14d** (Figure 7d). In the aromatic region of NH cyclic trimer **14b**, the proton signals appeared at lower field relative to those of N-decyloxybenzyl cyclic trimer **13b**. This tendency was also observed for **14c** and **14d**. These results indicate that the aromatic protons of the cyclic tertiary amide compounds (**13b**–**13d**) are more shielded than those of the cyclic secondary amide compounds (**14b**–**14d**), consistent with the *cis* conformation of the tertiary amides. This suggests that the benzene rings of the cyclic tertiary amide compounds **13b**–**13d** are in close proximity to each other, giving folded conformations in solution. On the other hand, the downfield shifts in the aromatic signals in the cyclic secondary amide compounds **14b**–**14d** suggest that these compounds adopt open structures predominantly in solution.

In contrast, the signals of cyclic dimer in the aromatic region showed little difference in chemical shifts between **13a** and **14a** (Figure 7a), indicating that secondary and tertiary amide of cyclic dimer adopt *cis* conformation due to structural constraints.

### 2.5. Conformational Analysis of Cyclic Oligomers

The conformations observed in solution often differ from the calculated low-energy structures because of solvent and entropic effects. Nevertheless, with an appropriate conformational analysis, the stable conformations of a macrocycle can be predicted with reasonable accuracy [29]. To investigate the conformational preferences of the diphenyladamantane-containing macrocyclic amides, conformational analyses based on force field calculations were performed. As a first step, the conformational features of secondary and tertiary aromatic amides were examined using benzanilide and *N*-methylbenzanilide as model compounds. The calculations were performed using the Monte Carlo Multiple Minimum (MCMM) method with the OPLS4 force field in CHCl_3_, as the implicit solvent. The most stable conformers obtained from these calculations were compared with previously reported their crystal structures.

**Figure 8:**
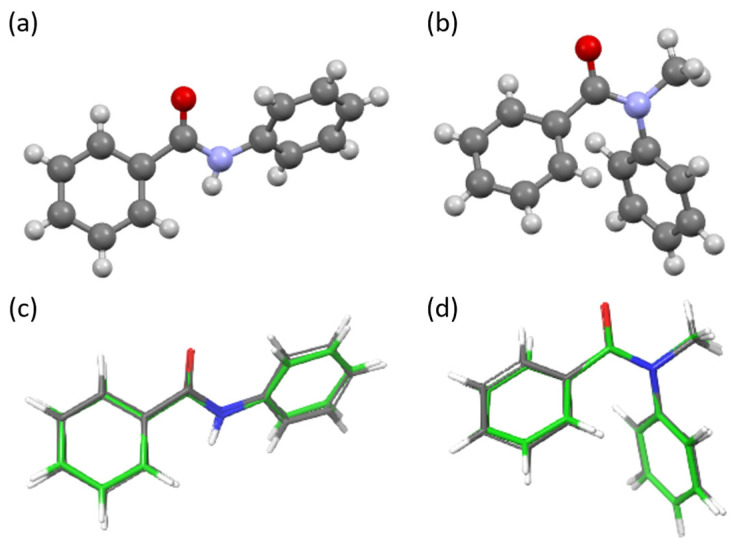
Crystal structures of (**a**) benzanilide (CSD refcode BZANIL02), and (**b**) *N*-methylbenzanilide (CSD refcode JAZJOJ10). (**c**,**d**) Overlay diagrams of the crystal structures and calculated molecular structures of benzanilide (**c**) and *N*-methylbenzanilide (**d**). Calculated structures are shown in green; atoms in the crystal structure are color-coded as follows: C (gray), O (red), N (blue), and H (white).

For benzanilide, the lowest-energy conformer identified was the *trans* isomer, in excellent agreement with the reported crystal structure (Figure 8a,c) [30]. The energy difference between the *trans* and *cis* conformers was 19.671 kcal/mol, consistent with the absence of the *cis* isomer in solution [31]. In contrast, for *N*-methylbenzanilide, the lowest-energy conformer was in the *cis* configuration, which also matched well with its reported crystal structure (Figure 8b,d). The *trans* conformer was higher in energy by 10.972 kcal/mol, in good agreement with the experimentally observed *cis*:*trans* ratio of 99:1 in CDCl_3_ solution. Notably, in the *cis*-form of *N*-methylbenzanilide, the spatial proximity of the two aromatic rings results in significant upfield shifts in the ^1^H NMR signals compared with the *trans*-form of benzanilide [31].

Based on these findings, we conducted conformational analysis of the NH cyclic trimer (**14b**), cyclic tetramer (**14c**), and cyclic pentamer (**14d**) under the same computational conditions. The resulting lowest-energy structures were compared with their corresponding crystal structures of the N-ethyl derivatives and those expected from ^1^H NMR spectral data in solution.

**Figure 9:**
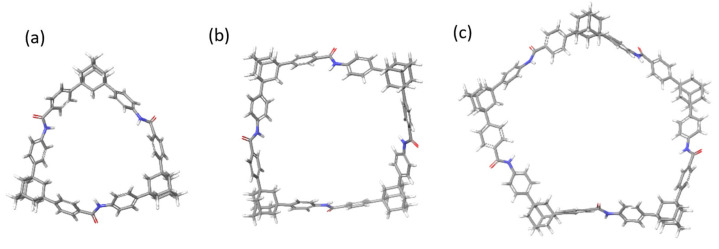
Calculated molecular structures of (**a**) **14b**, (**b**) **14c**, and (**c**) **14d**. Atoms are color-coded as follows: C (gray), O (red), N (blue), and H (white).

The NH cyclic trimer **14b**, composed of secondary amides, adopted an all-*trans* conformation in its lowest-energy structure (Figure 9a). In contrast, the N-ethyl cyclic trimer **11b**, composed of tertiary amides, exhibited an all-*cis* conformation in crystal structure (Figure 5a). These structural differences correlated well with the observed downfield shifts in the aromatic proton signals in the NH cyclic trimer relative to the N-ethyl analog. A similar trend was observed for the corresponding tetramers **11c** and **14c** (Figure 6a and Figure 9b).

For the pentamers (**11d** and **14d**), no crystal structure of the N-ethyl derivative **11d** was obtained. However, ^1^H NMR spectra suggested a predominant population of *cis*-amide conformations in solution (Figure 7d). In contrast, both conformational analysis and ^1^H NMR data for the NH cyclic pentamer **14d** indicated that it preferentially adopted an all-*trans* configuration (Figure 9c).

Taken together, the comparison of ^1^H NMR spectra, crystal structures, and computational conformational analyses revealed a consistent conformational behavior across the macrocyclic trimers to pentamers. Macrocyclic aromatic amides with tertiary amide linkages preferentially adopt a folded conformation with all *cis*-amide bonds in solution, whereas those with secondary amides preferentially adopt open conformations with all *trans*-amide bonds.

## 3. Materials and Methods

### 3.1. General

All starting materials and solvents were purchased from Aldrich (Tokyo, Japan), Wako (Osaka, Japan), and TCI Co., Ltd. (Tokyo, Japan). All commercially available reagents and solvents were used without further purification. FT-IR spectra were recorded on a JASCO (Tokyo, Japan) FT/IR-4100 using KBr tablets. ^1^H NMR spectra were recorded on a JEOL (Tokyo, Japan) JNM-ECS400 (400 MHz) spectrometer. Chemical shifts (δ) are given from TMS (0 ppm) in CDCl_3_, DMSO-*d*_6,_ and coupling constants are expressed in hertz (Hz). The following abbreviations are used: s = singlet, d = doublet, t = triplet, q = quartet, dd = double doublet, m = multiplet. ^13^C-NMR spectra were recorded on JEOL JNM-ECS400 (100 MHz) spectrometer. Chemical shifts (δ) are given from ^13^CDCl_3_ (77.0 ppm). Mass spectroscopy and high-resolution mass spectroscopy were measured on a JEOL JMS700 MStation (FAB), Thermo Fisher Scientific (Waltham, MA, USA) Exactive (APCI), and ultrafleX-treme Bruker (Billerica, MA, USA) (MALDI-TOF-MS). These samples were dissolved in CHCl_3_ at a concentration of 10 mg/mL. DCTB (3-(4-tert-Butylphenyl)-2-methyl-2-propenylidene malononitrile) or dithranol or DHB (2,5-dihydroxybenzoic acid) were chosen as sample matrices and dissolved in CHCl_3_ or CHCl_3_:THF = 1:1. Dissolved samples were mixed with matrices (1:10 (*v*/*v*)) and applied to the plate for analysis. Preparative recycling gel permeation chromatography (GPC) was performed with a Japan Analytical Industry (Tokyo, Japan) LC-9204 instrument equipped with JAIGEL-1H/JAIGEL-2H columns using chloroform as an eluent.

### 3.2. Synthesis

1,3-Diphenyladamantane (**2**) [22]

1-Bromoadamantane (**1**) (20 g, 93 mmol) was dissolved in benzene (100 mL). Then *tert*-BuBr (10 mL) and AlCl_3_ (0.42 g, 1.9 mmol) were added, and the mixture was stirred at 85 °C for 10 min. After cooling, the reaction mixture was poured into water. Benzene was added with stirring for 1 h, and the mixture was filtered to remove a white solid. The organic layer was dried over anhydrous MgSO_4_ and concentrated in vacuo to give a crude product. The residue was purified by flash column chromatography (silica gel, toluene:*n*-hexane = 1:10) to give **2** (8.9 g, 31 mmol, 33%) as a white solid. M.p.: 96–98 °C. ^1^H NMR (400 MHz, 298 K, CDCl_3_): δ 7.40 (d, *J* = 8.2 Hz, 4H), 7.32 (t, *J* = 7.5 Hz, 4H), 7.19 (t, *J* = 7.1 Hz, 2H), 2.32 (m, 2H), 2.05 (s, 2H), 1.96 (d, *J* =2.9 Hz, 8H), 1.79 (t, *J* = 2.9 Hz, 2H). ^13^C NMR (100 MHz, 298 K, CDCl_3_): δ 150.5, 128.1, 125.7, 124.8, 48.9, 42.2, 37.2, 35.8, 29.5. FT–IR (KBr, cm^−1^): 3058, 2904, 2850, 1600, 1495, 1445. MS (FAB): *m/z* 289 [M + H]^+^. Anal. Calcd. for C_22_H_24_: C, 91.61; H, 8.39; Found: C, 91.21; H, 8.08.

1-(4-Nitrophenyl)-3-phenyladamantane (**3**) [22]

Compound **2** (3.5 g, 12 mmol) was dissolved in dichloromethane (16 mL) and acetic acid (11 mL). Then, acetic anhydride (4.3 mL) and fuming nitric acid (9.2 mL) in acetic acid (11 mL) were added dropwise into the solution at 0 °C. The reaction mixture was refluxed at 50 °C under an argon atmosphere. After 30 min, ethyl acetate was added to the reaction mixture. The organic layer was washed with water, sat. NaHCO_3_ and brine, dried over MgSO_4_ and evaporated to give a crude product. The crude product was purified by silica gel column chromatography (chloroform:*n*-hexane = 1:4) to give **3** (1.8 g, 5.3 mmol, 43%) as a white solid. M.p.: 101–103 °C. ^1^H NMR (400 MHz, 298 K, CDCl_3_): δ 8.17 (d, *J* = 8.9 Hz, 2H), 7.54 (d, *J* = 9.2 Hz, 2H), 7.39 (d, *J* = 7.3 Hz, 2H), 7.34 (t, *J* = 7.3 Hz, 2H), 7.21 (t, *J* = 7.3 Hz, 1H), 2.39–2.33 (m, 2H), 2.04 (s, 2H), 1.98 (dd, *J* = 6.7, 3.3 Hz, 8H), 1.83–1.79 (m, 2H). ^13^C NMR (100 MHz, 398 K, CDCl_3_): δ 158.1, 149.9, 128.2, 125.9, 124.7, 123.4, 48.5, 41.9, 41.9, 38.0, 37.1, 35.5, 29.3. FT–IR (KBr, cm^−1^): 2901, 2852, 1598, 1514, 1446, 1355. MS (FAB): *m/z* 334 [M + H]^+^. Anal. Calcd. for C_22_H_23_NO_2_: C, 79.25; H, 6.95; N, 4.20, Found: C, 79.00; H, 6.96; N, 4.16.

1-(4-Iodophenyl)-3-(4-nitrophenyl)adamantane (**4**) [22]

Compound **3** was dissolved in chloroform (28 mL). Then [bis(trifluoroacetoxy)iodo]benzene (2.1 g, 5.0 mmol) and I_2_ (1.1 g, 4.3 mmol) were added to the solution. The mixture was stirred at 50 °C under an argon atmosphere. After 2 h, chloroform was added to the reaction mixture. The reaction mixture was quenched with 5% Na_2_S_2_O_3_, and washed with water and brine, dried over. Na_2_SO_4_ and evaporated to give a crude product. The crude product was purified by silica gel column chromatography (*n*-hexane) to give **4** (1.5 g, 3.4 mmol, 86%) as a colorless solid. M.p.: 150–152 °C. ^1^H NMR (400 MHz, 298 K, CDCl_3_): δ 8.17 (d, *J* = 8.9 Hz, 2H), 7.64 (d, *J* = 8.7 Hz, 2H), 7.53 (d, *J* = 8.9 Hz, 2H), 7.13 (d, *J* = 8.4 Hz, 2H), 2.39–2.33 (m, 2H), 1.99–1.91 (m, 10H), 1.81–1.79 (m, 2H). ^13^C NMR (100 MHz, 298 K, CDCl_3_): δ 157.8, 149.6, 137.3, 127.0, 125.9, 123.4, 91.2, 48.3, 41.8, 41.7, 38.0, 37.0, 35.4, 29.1. FT–IR (KBr, cm^−1^): 2923, 2852, 1595, 1507, 1338. Anal. Calcd. for C_22_H_22_INO_2_·0.3 H_2_O: C, 56.86; H, 4.90; N, 3.01, Found: C, 56.62; H, 4.78; N, 2.96. Satisfactory MS data could not be obtained for this compound.

1-(4-Cyanophenyl)-3-(4-nitrophenyl)adamantane (**5**) [22]

Compound **4** (0.60 g × 2, 13 mmol) was dissolved in pyridine (40 mL). Then Copper (I) cyanide (1.2 g, 13 mmol) was added. The mixture was stirred at 120 °C for 22 h under an argon atmosphere. After cooling, Et_2_O was added to the reaction mixture. The organic layer was washed with an aqueous solution of ammonia, 2 M HCl, and brine, then dried over Na_2_SO_4,_ and evaporated to give a crude product. The crude product was purified by silica gel column chromatography (toluene) to give **5** (0.71 g, 1.9 mmol, 85%) as a yellow solid. M.p.: 170–172 °C. ^1^H NMR (400 MHz, 298 K, CDCl_3_): δ 8.18 (d, *J* = 8.9 Hz, 2H), 7.63 (d, *J* = 8.5 Hz, 2H), 7.54 (d, *J* = 8.9 Hz, 2H), 7.49 (d, *J* = 8.5 Hz, 2H), 2.42–2.37 (m, 2H), 2.02–1.97 (m, 10H), 1.84–1.82 (m, 2H). ^13^C NMR (100 MHz, 298 K, CDCl_3_): δ 157.4, 155.2, 146.1, 132.1, 125.8, 125.7, 123.5, 118.9, 109.8, 48.0, 41.7, 41.5, 37.9, 35.3, 29.0. FT–IR (KBr, cm^−1^): 3075, 2917, 2848, 2224, 1594, 1516, 1343. MS (FAB): *m/z* 359 [M + H]^+^. Anal. Calcd. for C_23_H_22_N_2_O_2_·0.2 H_2_O: C, 76.30; H, 6.24; N, 7.74, Found: C, 76.18; H, 6.13; N, 7.65.

4-[3-(4-Nitrophenyl)adamantan-1-yl]benzoic acid (**6**)

Compound **5** (0.92 g, 2.5 mmol) was dissolved in EtOH (80 mL). Then 4 M NaOH aq. (80 mL) was added. The mixture was stirred at 80 °C under an argon atmosphere. After 18 h, the reaction mixture was evaporated to remove ethanol, and the residual aqueous mixture was neutralized with 6 M HCl with cooling and subsequently extracted with ethyl acetate. The organic layer was successively washed with brine, dried over MgSO_4,_ and evaporated to give a crude product. The crude product was purified by trituration using chloroform to give compound **6** (0.9 g, 2.3 mmol, 90%) as a white solid. M.p.: 262–263 °C. ^1^H NMR (400 MHz, 313 K, DMSO-*d*_6_): δ 8.17 (d, *J* = 8.8 Hz, 2H), 7.89 (d, *J* = 8.2 Hz, 2H), 7.74 (d, *J* = 8.9 Hz, 2H), 7.56 (d, *J* = 8.4 Hz, 2H), 2.31–2.26 (m, 2H), 2.04 (s, 2H), 1.98–1.90 (m, 8H), 1.78–1.76 (m, 2H). ^13^C NMR (100 MHz, 313 K, DMSO-*d*_6_): δ 167.2, 158.0, 155.0, 145.5, 129.2, 128.2, 126.5, 125.1, 123.2, 47.1, 41.1, 41.0, 39.3, 37.2, 34.8, 28.7. FT–IR (KBr, cm^−1^): 3852, 3749, 3734, 3647, 3628, 2358, 1683, 1520, 1346. MS (FAB): *m/z* 378 [M + H]^+^. Anal. Calcd. for C_23_H_23_NO_4_: C, 73.19; H, 6.14; N, 3.71, Found: C, 72.71; H, 6.11; N, 3.73.

Ethyl 4-[3-(4-nitrophenyl)adamantan-1-yl]benzoate (**7**)

Compound **6** (2.2 g, 5.9 mmol) was dissolved in EtOH (182 mL). Then conc. H_2_SO_4_ (3.0 mL) was added. The mixture was stirred at 110 °C under an argon atmosphere. After 2 h, the reaction mixture was evaporated to vaporize ethanol and neutralized with 4M NaOH aq. with cooling and subsequently extracted with chloroform. The organic layer was successively washed with brine, dried over MgSO_4,_ and evaporated to give a crude product. The crude product was purified by silica gel column chromatography (chloroform:*n*-hexane = 1:1) to give **7** (2.2 g, 5.3 mmol, 90%) as a white solid. M.p.: 170–171 °C. ^1^H NMR (400 MHz, 298 K, CDCl_3_): δ 8.18 (d, *J* = 8.7 Hz, 2H), 8.00 (d, *J* = 6.6 Hz, 2H), 7.55 (d, *J* = 8.7 Hz, 2H), 7.45 (d, *J* = 6.6 Hz, 2H), 4.37 (q, *J* = 7.1 Hz, 2H), 2.40–2.36 (m, 2H), 2.5 (s, 2H), 2.00–1.98 (m, 8H), 1.83–1.81 (m, 2H). ^13^C NMR (100 MHz, 298 K, CDCl_3_): δ 166.5, 157.7, 155.0, 146.0, 129.5, 128.2, 125.9, 124.8, 123.5, 60.8, 48.2, 41.8, 41.7, 38.0, 37.5, 35.4, 29.2. FT–IR (KBr, cm^−1^): 2980, 2931, 2847, 1718, 1653, 1559, 1451, 1411, 1387, 1368. MS (FAB): *m/z* 406 [M + H]^+^. Anal. Calcd. for C_25_H_27_NO_4_: C, 74.05; H, 6.71; N, 3.45, Found: C, 73.68; H, 6.50; N, 3.40.

Ethyl 4-(3-(4-aminophenyl)adamantan-1-yl)benzoate (**8**)

Compound **7** (2.0 g, 5.3 mmol) was dissolved in EtOH (300 mL). Then Pd/C (0.30 g) was added. The mixture was stirred at 50 °C under a hydrogen atmosphere. After 15 h, the reaction mixture was filtered to remove the catalyst. The organic layer was evaporated to give a crude product. The crude product was purified by silica gel column chromatography (ethyl acetate:toluene = 1:15) to give **8** (1.7 g, 4.6 mmol, 92%) as a white solid. M.p.: 97 °C. ^1^H NMR (400 MHz, 298 K, CDCl_3_): δ 7.99 (d, *J* = 8.7 Hz, 2H), 7.45 (d, *J* = 8.4 Hz, 2H), 7.18 (d, *J* = 8.4 Hz, 2H), 6.67 (d, *J* = 8.4 Hz, 2H), 4.36 (q, *J* = 7.1 Hz, 2H), 3.57 (s, 2H), 2.31–2.30 (m, 2H), 1.99 (s, 2H), 1.94–1.92 (m, 8H), 1.79–1.78 (m, 2H), 1.38 (t, *J* = 7.10 Hz, 3H). ^13^C NMR (100 MHz, 298 K, CDCl_3_): δ 166.6, 155.9, 144.1, 140.8, 129.4, 127.8, 125.6, 124.9, 115.0, 60.7, 48.9, 42.3, 42.1, 37.7, 36.4, 35.8, 29.4, 14.3. FT–IR (KBr, cm^−1^): 3441, 3358, 2906, 1707, 1624, 1517, 1281, 1110, 1018, 822, 778, 711. MS (FAB): *m/z* 358 [M + H]^+^. Anal. Calcd. for C_25_H_29_NO_2_: C, 79.96; H, 7.78; N, 3.73, Found: C, 80.28; H, 7.81; N, 3.68.

Ethyl 4-{3-[4-(ethylamino)phenyl]adamantan-1-yl}benzoate (**9**)

Compound **8** (0.8 g, 0.2 mmol) was dissolved in THF (1.0 mL). Then acetonitrile (50 mL) and 10% Pd/C (0.25 g) were added to the solution. The mixture was stirred at room temperature under a hydrogen atmosphere. After 36 h, the reaction mixture was added to acetonitrile (10 mL) and 10% Pd/C (0.25 g) and stirred for 25 h. The reaction mixture was filtrate using cerite under reduced pressure to give **9** (56 mg, 0.14 mmol, 69%) as a white solid. M.p.: 80–81 °C. ^1^H NMR (400 MHz, 298 K, CDCl_3_): δ 7.98 (d, *J* = 8.7 Hz, 2H), 7.45 (d, *J* = 8.7 Hz, 2H), 7.20 (d, *J* = 8.9 Hz, 2H), 6.59 (d, *J* = 8.7 Hz, 2H), 4.36 (dd, *J* = 7.1, 7.1 Hz, 2H), 7.33 (dd, *J* = 7.1, 7.1 Hz, 2H), 2.31–2.29 (m, 2H), 1.99 (s, 2H), 1.94–1.91 (m, 8H), 1.79–1.75 (m, 2H), 1.38 (t, *J* = 7.1 Hz, 3H), 1.24 (t, *J* = 7.1 Hz, 3H). ^13^C NMR (100 MHz, 298 K, CDCl_3_): δ 166.7, 156.1, 146.2, 139.4, 129.5, 127.9, 127.6, 125.0, 112.6, 60.8, 49.0, 42.4, 42.2, 38.7, 37.8, 36.4, 35.9, 29.6, 15.0, 14.4. FT–IR (KBr, cm^−1^): 3388, 2895, 1703, 1523, 1289, 771. MS (FAB): *m/z* 404 [M + H]^+^. Anal. Calcd. for C_27_H_33_NO_2_: C, 80.36; H, 8.24; N, 3.47, Found: C, 79.99; H, 7.98; N, 3.28.

4-(3-(4-(Ethylamino)phenyl)adamantan-1-yl)benzoic acid (**10**)

Compound **9** (1.0 g, 2.5 mmol) was dissolved in EtOH (80 mL). Then 4 M NaOH aq. (80 mL) was added. The mixture was heated at 80 °C for 6 h under an argon atmosphere. After removal of ethanol in vacuo, the aqueous solution was acidified by addition of 6 M HCl. The water layer was filtrate under reduced pressure to give compound **10** (0.71 g, 1.9 mmol, 78%) as a pink solid. M.p.: 255–256 °C. ^1^H NMR (400 MHz, 298 K, DMSO-*d*_6_): δ 7.88 (d, *J* = 8.5 Hz, 2H), 7.52 (d, *J* = 8.5 Hz, 2H), 7.11 (d, *J* = 8.7 Hz, 2H), 6.50 (d, *J* = 8.7 Hz, 2H), 2.99 (q, *J* = 7.3, 7.1 Hz, 2H), 2.26–2.19 (m, 2H), 1.93–1.67 (m, 12H), 1.12 (t, *J* = 7.1 Hz, 3H). ^13^C NMR (100 MHz, 298 K, DMSO-*d*_6_): δ167.3, 155.6, 146.7, 137.8, 129.3, 128.1, 125.1, 111.9, 48.6, 41.9, 41.5, 37.6, 37.4, 35.8, 35.3, 29.0, 14.5. FT–IR (KBr, cm^−1^): 3284, 2899, 2852, 1686, 1609, 1513, 1448. MS (FAB): *m/z* 376 [M + H]^+^. Anal. Calcd. for C_25_H_29_NO_2_: C, 79.96; H, 7.78; N, 3.73, Found: C, 79.68; H, 7.68; N, 3.67.

Cyclization reaction

Synthesis from **9**

To a solution of the compound **9** (0.081 g, 0.2 mmol) in dry THF (0.4 mL) under argon atmosphere was added 1.3 M LiHMDS solution in THF (0.15 mL, 0.21 mmol) and stirred at room temperature for 19 h. The organic layer was washed with water, 2 M HCl, and brine over Na_2_SO_4_, and concentrated in vacuo. The residue was purified by silica gel column chromatography (chloroform), followed by preparative GPC (chloroform as an eluent) to give products **11a**–**11d**.

Synthesis from **10**

To a solution of the compound **10** (0.075 g, 0.20 mmol) in (CHCl_2_)_2_ (4 mL) under an argon atmosphere was added Ph_3_PCl_2_ (0.24 g, 7.2 mmol), and the mixture was stirred at 120 °C for 7 h. After cooling, the reaction mixture was poured into water and extracted with chloroform. The organic layer was washed with 2 M HCl, sat. NaHCO_3_ and brine, dried over Na_2_SO_4_, and concentrated in vacuo. The residue was purified by silica gel column chromatography (chloroform), followed by preparative GPC (chloroform as an eluent) to give products **11a**–**11d**.

Compound **11a** (N-ethyl cyclic dimer)

Yield: 29 mg (0.041 mmol), 40%. Color, Habit: white, powder. M.p.: >300 °C. ^1^H NMR (400 MHz, 298 K, CDCl_3_): δ 7.19 (d, *J* = 8.4 Hz, 2H), 7.15 (d, *J* = 8.4 Hz, 2H), 7.07 (d, *J* = 8.4 Hz, 2H), 6.91 (d, *J* = 8.4 Hz, 2H), 3.95 (q, *J* = 6.8Hz, 2H), 2.29 (s, 2H), 2.05 (d, *J* = 8.0 Hz, 2H), 1.95 (d, *J* = 12 Hz, 2H), 1.77 (d, *J* = 11.6 Hz, 4H), 1.68 (s, 2H), 1.60 (s, 2H), 1.21 (t, *J* = 7.2 Hz, 3H) ^13^C NMR (100 MHz, 298 K, CDCl_3_): δ 169.9, 151.9, 148.9, 141.2, 133.6, 129.0, 127.3, 125.6, 124.1, 51.0, 45.6, 41.6, 41.3, 37.0, 35.6, 29.8, 29.4, 13.0. FT–IR (KBr, cm^−1^): 2926, 2902, 2243, 1626, 1606, 1513. MS (FAB): *m/z* 715 [M + H]^+^. Anal. Calcd. for C_50_H_54_N_2_O_2_·1.1CHCl_3_: C, 72.52; H, 6.56; N, 3.31, Found: C, 72.20; H, 6.95; N, 3.05.

Compound **11b** (N-ethyl cyclic trimer)

Yield: 5.0 mg (4.7 mmol), 6.9%. Color, Habit: white, powder. M.p.: 160–165 °C. ^1^H NMR (400 MHz, 298 K, CDCl_3_): δ 7.21 (d, *J* = 8.7 Hz, 6H), 7.17 (d, *J* = 8.7 Hz, 6H), 7.12 (d, *J* = 8.7 Hz, 6H), 6.92 (d, *J* = 8.7 Hz, 6H), 3.96 (q, *J* = 7.1 Hz, 6H), 2.21 (m, 6H), 1.90–1.82 (m, 18H), 1.74–1.69 (m, 18H), 1.22 (t, *J* = 7.0 Hz, 9H). ^13^C NMR (100 MHz, 298 K, CDCl_3_): δ 134.4, 129.4, 128.2, 126.0, 124.7, 47.8, 45.9, 43.2, 42.7, 37.8, 37.6, 36.4, 30.0, 13.6. FT–IR (KBr, cm^−1^): 2916, 1508, 820. HRMS (APCI): *m/z* [M + H]^+^ calcd for C_75_H_82_O_3_N_3_: 1072.6351; found: 1072.6356.

Compound **11c** (N-ethyl cyclic tetramer)

Yield: 3.5 mg (2.4 mmol), 4.8%. Color, Habit: yellow, prism crystals. M.p.: 158–160 °C. ^1^H NMR (400 MHz, 298 K, CDCl_3_): δ 7.22 (d, *J* = 8.4 Hz, 8H), 7.19 (d, *J* = 8.7 Hz, 8H), 7.12 (d, *J* = 8.4 Hz, 8H), 6.95 (d, *J* = 8.4 Hz, 8H), 3.94 (q, *J* = 6.8 Hz, 8H), 2.27–2.20 (m, 8H), 1.91–1.69 (m, 48H), 1.20 (t, *J* = 7.1 Hz, 3H). ^13^C NMR (100 MHz, 298 K, CDCl_3_): δ 151.8, 148.9, 133.8, 128.7, 127.5, 125.5, 124.2, 116.5, 48.4, 45.5, 42.1, 42.1, 37.3, 37.0, 35.7, 29.7, 29.4, 13.1. FT–IR (KBr, cm^−1^): 2919, 1645, 1509, 1278, 1015. HRMS (APCI): *m/z* [M + H]^+^ calcd for C_100_H_109_O_4_N_4_: 1429.8443; found: 1429.8449.

Compound **11d** (N-ethyl cyclic pentamer)

Yield: 1.4 mg (0.78 mmol), 1.9%. Color, Habit: yellow, prism crystals. M.p.: 152–153 °C. ^1^H NMR (400 MHz, 298 K, CDCl_3_): δ 7.25–7.11 (m, 30H), 7.00–6.94 (m,10H), 3.97–3.86 (m, 10H), 2.27–2.20 (m, 10H), 1.95–1.72 (m, 60H), 1.22–1.17 (m, 15H). ^13^C NMR (100 MHz, 298 K, CDCl_3_): δ 152.4, 149.5, 129.3, 128.1, 126.1, 124.7, 46.1, 42.7, 42.6, 37.9, 37.6, 36.3, 30.0, 13.7. FT–IR (KBr, cm^−1^):3645, 2903, 1644, 1512, 1385, 1117. HRMS (APCI): *m/z* [M + H]^+^ calcd for C_125_H_136_O_5_N_5_: 1787.0536; found: 1787.0498.

Ethyl 4-(3-(4-((4-(decyloxy)benzyl)amino)phenyl)adamantan-1-yl)benzoate (**12**)

Compound **8** (0.24 g, 0.64 mmol) was dissolved in ethanol:acetic acid (10:1). Then, 4-decyloxybenzylaldehyde and borane-2-methylpyridine were added to the solution. The mixture was stirred at room temperature under an argon atmosphere. After 1 h, 10% HCl (3.3 mL) was added to the reaction mixture, which was stirred for 30 min. The mixture was then neutralized with 0.25% Na_2_CO_3_ and extracted with ethyl acetate. The organic layer was dried over Na_2_SO_4_ and concentrated in vacuo to give the crude product. The crude product was purified by silica gel column chromatography (toluene) to give **12** (0.38 g, 0.61 mmol, 89%) as a white solid. M.p.: 100 °C, ^1^H NMR (400 MHz, 298 K, CDCl_3_): δ 7.98 (d, *J* = 1.8 Hz, 2H), 7.45 (d, *J* = 1.8 Hz, 2H), 7.20 (d, *J* = 1.8 Hz, 2H), 6.86 (d, *J* = 1.8 Hz, 2H), 6.61–6.64 (m, 2H), 4.34–4.39 (m, 2H), 4.23 (s, 2H), 3.94 (t, *J* = 6.6 Hz, 2H), 3.87 (s, 1H), 2.30 (s, 2H), 2.00 (s, 2H), 1.92–1.94 (m, 8H), 1.73–1.80 (m, 4H), 1.22–1.48 (m, 18H), 0.86–0.90 (m, 3H). ^13^C NMR (100 MHz, 298 K, CDCl_3_): δ 166.6, 158.4, 155.9, 146.2, 139.6, 131.3, 129.4, 128.8, 127.8, 125.6, 124.9, 114.5, 112.6, 68.0, 60.7, 48.9, 48.0, 42.3, 42.1, 37.7, 36.3, 35.8, 31.8, 29.5, 29.5, 29.3, 29.3, 29.2, 26.0, 22.6, 14.3, 14.1. FT–IR (KBr, cm^−1^): 3851, 2357, 1698, 1514, 1471, 1278, 1103, 1015, 823. MS (FAB): *m/z* 620 [M + H]^+^. Anal. Calcd. for C_42_H_55_NO_3_: C, 81.12; H, 8.91; N, 2.25, Found: C, 80.89; H, 8.87; N, 2.25.

Cyclization reaction

To a solution of the compound **12** (0.12 g, 0.19 mmol) in dry THF (0.4 mL) under argon atmosphere was added 1.3 M LiHMDS solution in THF (0.31 mL, 0.21 mmol) and stirred at room temperature for 2 h. The organic layer was washed with water, 2 M HCl, and brine over Na_2_SO_4_, and concentrated in vacuo. The residue was purified by silica gel column chromatography (chloroform), followed by preparative GPC (chloroform as an eluent) to give products **13a**–**13d**.

Compound **13a** (N-decyloxybenzyl cyclic dimer)

Yield: 47 mg (0.041 mmol), 41%. Color, Habit: white, powder. M.p.: 110–121 °C. ^1^H NMR (400 MHz, 298 K, CDCl_3_): δ 7.22 (d, *J* = 2.2 Hz, 8H), 7.06 (d, *J* = 1.3 Hz, 8H), 6.78–6.82 (m, 8H), 5.01 (s, 4H), 3.91 (t, *J* = 6.6 Hz, 4H), 2.28 (s, 4H), 2.01 (d, *J* = 11.9 Hz, 4H), 1.92 (d, *J* = 11.9 Hz, 4H), 1.72–1.78 (m, 12H), 1.67 (s, 4H), 1.53 (s, 4H), 1.40–1.47 (m, 4H), 1.27–1.35 (m, 24H), 0.86–0.90 (m, 6H). ^13^C NMR (100 MHz, 298 K, CDCl_3_): δ 158.3, 151.9, 148.7, 141.5, 133.2, 129.9, 129.6, 128.9, 127.0, 125.3, 124.0, 114.2, 67.9, 41.4, 41.1, 37.2, 36.8, 31.8, 29.5, 29.5, 29.4, 29.2, 29.2, 26.0, 22.6, 14.1. FT–IR (KBr, cm^−1^): 3850, 3707, 3646, 2355, 667, 652, 643, 611. HRMS (APCI): *m/z* [M + H]^+^ calcd for C_80_H_99_O_4_N_2_: 1151.7502; found: 1151.7603.

Compound **13b** (N-decyloxybenzyl cyclic trimer)

Yield: 8.4 mg (4.9 mmol), 7.2%. Color, Habit: white, powder. M.p.: 92–105 °C. ^1^H NMR (400 MHz, 298 K, CDCl_3_): δ 7.23–7.18 (m, 12H), 7.11–7.06 (m, 12H), 6.83–6.76 (m, 12H), 5.01 (s, 6H), 3.92 (t, *J* = 6.6 Hz, 6H), 2.29–2.18 (m, 8H), 1.93–1.65 (m, 46H), 1.45–1.40 (m, 8H), 1.35–1.27 (m, 42H), 0.90–0.86 (m, 9H). ^13^C NMR (100 MHz, 298 K, CDCl_3_): δ 170.3, 158.3, 151.8, 148.8, 141.3, 133.5, 129.8, 129.7, 129.6, 128.8, 127.4, 125.4, 125.2, 124.0, 114.2, 70.5, 67.9, 54.4, 53.1, 47.1, 42.5, 42.0, 37.1, 36.9, 35.7, 31.8, 29.7, 29.5, 29.5, 29.4, 29.3, 29.3, 26.1, 22.6, 14.1. FT–IR (KBr, cm^−1^): 3910, 3849, 3590, 3048, 2958, 2927, 2846, 2800, 2355, 1727, 1642, 1515, 1375, 1255, 1096, 1015. HRMS (APCI): *m/z* [M + H]^+^ calcd for C_120_H_148_O_6_N_3_: 1727.1253; found: 1727.1402.

Compound **13c** (N-decyloxybenzyl cyclic tetramer)

Yield: 4.9 mg (2.2 mmol), 4.3%. Color, Habit: white, powder. M.p.: 75–87 °C. ^1^H NMR (400 MHz, 298 K, CDCl_3_): δ 7.25–7.00 (m, 32H), 6.88–6.71 (m, 16H), 5.00 (t, *J* = 6.8 Hz, 8H), 3.94–3.83 (m, 8H), 2.33–2.10 (m, 8H), 1.96–1.62 (m, 58H), 1.49–1.15 (m, 58H), 0.87 (t, *J* =6.4 Hz, 12H). ^13^C NMR (100 MHz, 298 K, CDCl_3_): δ 158.5, 129.8, 128.9, 127.4, 125.5, 124.2, 124.1, 114.4, 68.0, 42.1, 37.3, 37.0, 32.0, 29.7, 29.7, 29.5, 29.4, 26.2, 22.8, 14.2. FT–IR (KBr, cm^−1^): 2930, 1651, 1508, 1245, 974. HRMS (MALDI-TOF): *m/z* [M + Na]^+^ calcd for C_160_H_196_O_8_N_4_Na: 2325.498; found: 2324.545.

Compound **13d** (N-decyloxybenzyl cyclic pentamer)

Yield: 4.8 mg (1.7 mmol), 4.2%. Color, Habit: white, powder. M.p.: 82–94 °C. ^1^H NMR (400 MHz, 298 K, CDCl_3_): δ 7.25–7.00 (m, 40H), 6.89–6.70 (m, 20H), 4.98 (s, 10H), 3.89 (q, *J* = 6.6, 4.5 Hz, 10H), 2.38–2.11 (m, 10H), 2.03–1.64 (m, 70H), 1.46–1.16 (m, 70H), 1.46–1.16 (m, 70H), 0.87 (t, *J* = 7.1 Hz,15H). ^13^C NMR (100 MHz, 298 K, CDCl_3_): δ 158.3, 129.8, 129.5, 128.8, 127.2, 125.3, 124.1, 114.2, 70.5, 67.9, 42.1, 41.9, 37.2, 37.1, 36.9, 31.8, 29.6, 29.5, 29.5, 29.4, 29.3, 26.1, 22.6, 14.1. FT–IR (KBr, cm^−1^): 3973, 3915, 3857, 3748, 3586, 2927, 2854, 2362, 2328, 1696, 1642, 1507, 1452, 1248. HRMS (MALDI-TOF): *m/z* [M + Na]^+^ calcd for C_200_H_245_O_10_N_5_Na: 2900.871; found: 2899.935.

Compound **14a** (NH cyclic dimer)

Compound **13a** (10 mg, 8.7 μmol) was dissolved in TFA (3.0 mL), and the resulting solution was stirred at 50 °C for 5 d. The reaction mixture was concentrated under reduced pressure to remove TFA. The residue was purified by centrifugation and resuspension in chloroform:toluene (1:15), yielding compound **14a** (1.2 mg, 1.8 mmol, 21%) as a white solid. M.p.: 300 °C. ^1^H NMR (400 MHz, 298 K, DMSO-*d*_6_): δ 9.47 (s, 2H), 7.27–7.09 (m, 12H), 6.71 (d, *J* = 8.4 Hz, 4H), 2.25 (s, 4H), 2.05–1.92 (m, 10H), 1.81–1.63 (m, 14H). ^13^C NMR (100 MHz, 298 K, DMSO-*d_6_*): δ 128.9, 128.2, 125.3, 124.0, 28.9, 28.7, 22.1, 21.0, 14.0. FT–IR (KBr, cm^−1^): 3852, 3748, 3674, 3647, 2908, 1540, 1517, 1376, 823, 693. MS (FAB): *m/z* 659 [M + H]^+^. Anal. Calcd. for C_46_H_46_N_2_O_2_·0.35 TFA: C, 80.27; H, 6.69; N, 4.01, Found: C, 80.21; H, 6.74; N, 4.40.

Compound **14b** (NH cyclic trimer)

Compound **13b** (4.5 mg, 2.1 µmol) was dissolved in TFA (2.6 mL), and the resulting solution was stirred at room temperature for 47 h. The reaction mixture was concentrated under reduced pressure to remove TFA. The residue was purified by centrifugation and resuspension in chloroform:toluene (1:15), yielding compound **14b** (1.7 mg, 1.7 mmol, 65%) as a white solid. M.p.: 300 °C. ^1^H NMR (400 MHz, 298 K, DMSO-*d*_6_): δ 9.84 (s, 4H), 7.84 (d, *J* = 8.7 Hz, 6H), 7.63 (d, *J* = 8.7 Hz, 6H), 7.45 (d, *J* = 8.4 Hz, 6H), 7.25 (d, *J* = 8.9 Hz, 6H), 2.35 (s, 6H), 2.21–2.16 (m, 12H), 1.85–1.75 (m, 18H), 1.34 (s, 6H). ^13^C NMR (100 MHz, 298 K, CDCl_3_ + 3 drops of TFA-*d*): δ134.3, 131.4, 129.5, 127.9, 126.8, 56.1, 43.4, 42.0, 39.6, 38.9, 36.7. FT–IR (KBr, cm^−1^): 3735, 2922, 2849, 1653, 1608, 1559, 1457, 1405, 1328, 1261, 1013. HRMS (FAB): *m/z* [M + H]^+^ calcd for C_69_H_70_O_3_N_3_: 988.5322; found: 988.5418.

Compound **14c** (NH cyclic tetramer)

Compound **13c** (3.6 mg, 1.6 μmol) was dissolved in TFA (1.9 mL), and the resulting solution was stirred at room temperature for 47 h. The reaction mixture was concentrated under reduced pressure to remove TFA. The residue was purified by centrifugation and resuspension in chloroform:toluene (1:15), yielding compound **14c** (1.6 mg, 1.2 mmol, 77%) as a white solid. M.p.: 300 °C. ^1^H NMR (400 MHz, 298 K, DMSO-*d*_6_): δ 7.94 (d, *J* = 7.3 Hz, 8H), 7.74 (d, *J* = 8.2 Hz, 8H), 7.61 (d, *J* = 9.8 Hz, 8H), 7.53 (d, *J* = 7.7 Hz, 8H), 2.46 (s, 8H), 2.12 (s, 42H), 1.95 (s, 14H). ^13^C NMR (100 MHz, 298 K, TFA-*d* +CDCl_3_): δ131.1, 129.2, 127.6, 126.6, 126.5, 125.1, 55.9, 49.9, 42.9, 39.4, 38.7, 36.5, 31.0. FT–IR (KBr, cm^−1^): 3854, 3736, 2905, 2848, 1654, 1519, 1407, 1014, 826, 635. HRMS (MALDI-TOF): *m/z* [M + Na]^+^ calcd for C_92_H_92_O_4_N_4_Na: 1339.6996; found: 1340.734.

Compound **14d** (NH cyclic pentamer)

Compound **13d** (1.9 mg, 0.66 μmol) was dissolved in TFA (1.1 mL), and the resulting solution was stirred at room temperature for 47 h. The reaction mixture was concentrated under reduced pressure to remove TFA. The residue was purified by centrifugation and resuspension in chloroform:toluene (1:15), yielding compound **14d** (0.72 mg, 0.44 mmol, 64%) as a white solid. M.p.: 103–105 °C. ^1^H NMR (400 MHz, 298 K, CDCl_3_): δ 7.84–7.81 (m, 10H), 7.60–7.58 (m, 10H), 7.53–7.50 (m, 10H), 7.41–7.38 (m, 10H), 2.35 (s, 14H), 2.04–1.98 (m, 50H), 1.81 (s, 14H). ^13^C NMR (100 MHz, 298 K, CDCl_3_): δ 130.2, 127.6, 126.2, 126.1, 120.8, 49.3, 42.8, 42.7, 38.3, 37.6, 36.4, 30.1. FT–IR (KBr, cm^−1^): 3421, 2761, 748, 700, 445. HRMS (MALDI-TOF): *m/z* [M + Na]^+^ calcd for C_115_H_115_O_5_N_5_Na: 1668.879; found: 1668.918.

## 4. Conclusions

We synthesized cyclic aromatic amides composed of repeating units incorporating a diphenyladamantane skeleton. Single crystals of the ethyl-substituted derivatives (cyclic dimers, trimers, and tetramers) were obtained, and single-crystal X-ray analysis confirmed that all tertiary amide bonds in these macrocyclic compounds adopt the *cis* configuration. Furthermore, the cyclic trimer and tetramer were shown to adopt folded conformations both in the crystal and in solutions.

Cyclic compounds bearing an N-decyloxybenzyl group were then synthesized by a similar route, and the corresponding cyclic aromatic secondary amides were obtained by dealkylation. Structural analysis based on ^1^H NMR spectra combined with force field calculations suggested that these secondary amides adopt the *trans* configuration. Thus, macrocyclic aromatic amides with tertiary amide bonds preferentially adopt folded ring conformations, whereas those with secondary amide bonds favor open ring conformations.

In summary, we have successfully synthesized large-ring compounds with spacious internal cavities and demonstrated that their conformations are governed by the presence or absence of N-alkyl substituents on the amide nitrogens. These macrocyclic structures provide promising scaffolds for further functionalization toward the development of molecular recognition systems capable of accommodating guest molecules of various sizes.

## Figures and Tables

**Table 1:** Product distributions of cyclic oligomers obtained from cyclization reactions using LiHMDS with the corresponding ester (Entry 1) and Ph_3_PCl_2_ with the corresponding carboxylic acid (Entry 2).

Entry	Monomer (R=)	Reagent	Equiv.	Solvent	Temp.	Time (h)	Yield (%) ^a^				
							n = 2 11a	3 11b	4 11c	5 11d	Total
1	**9** (Et)	LiHMDS	2.0 + 0.5 ^b^	THF	rt	24	35	13	5	1	54
2	**10** (H)	Ph_3_PCl_2_	3.6	(CHCl_2_)_2_	120 °C	7	76	11	3	<1	90

^a^ Including small amounts of impurities. ^b^ After 7 h, 0.5 equiv. of LiHMDS was added.

**Table 2:** Distributions of cyclic oligomers obtained from cyclization reactions of N-decyloxybenzyl monomer **12**.

	Reagent	Equiv.	Solvent	Temp. (°C)	Time (h)	Yield (%) ^a^			
						n = 2 13a	3 13b	4 13c	5 13d
Entry 1	LiHMDS	2.0	THF	rt	2	47	8	5	5

^a^ Including small amounts of impurities.

## Data Availability

Data will be made available on request.

## Supplementary Materials

The following supporting information can be downloaded at: https://www.mdpi.com/article/10.3390/molecules30214185/s1, Section S1—X-ray crystallographic analysis, Section S2—^1^H and ^13^C NMR spectra, Section S3—References; Figure S1—ORTEP diagram of molecules A, B, and C in a crystal of **11a**, Figure S2—ORTEP diagram of **11b**, Figure S3—ORTEP diagram of **11c**. Refs. [32,33,34] are cited in the Supplementary Materials.
