# Supplementary material for: Macrocyclic Aromatic Oligoamides with Diphenyladamantane Units: Conformational Change from Folded to Open by N-Alkyl Removal
Source: Molecules. 2025 Oct 26;30(21):4185. doi: 10.3390/molecules30214185 (PMC12608538; doi:10.3390/molecules30214185)
Supplement: Supplementary file 1 [file molecules-30-04185-s001.zip › molecules-3916471-supplementary.pdf]

Supporting Information

Macrocyclic Aromatic Oligoamides with Diphenyladamantane Units: Conformational Change from Folded to Open by N-Alkyl Removal

Sae Maeda[1], Ryota Usami[1], Kei Takamatsu[1], Moemi Takato[1], Shoko Kikkawa[1], Hidemasa Hikawa[1], Isao Azumaya[1]*

[1]Faculty of Pharmaceutical Sciences, Toho University, Miyama 2-2-1, Funabashi, Chiba 274-8510, Japan

*** Corresponding Author:** isao.azumaya@phar.toho-u.ac.jp

List of Contents

S1. X-ray crystallographic analysis 2
S2. ^{1}H NMR, ^{13}C NMR spectra 5
S3. References 27

S1. X-ray crystallographic analysis

X-ray data were collected on a Rigaku XtaLAB P200 diffractometer with multi-layer mirror monochromated Cu*K*α (λ = 1.54187Å) and a hybrid photon counting detector (PILATUS 200K). The crystal structure was solved by direct methods (SHELXT Version 2014/5) [32] and refined by full-matrix least-squares SHELXL-2018/3 [33]. All non-hydrogen atoms were refined anisotropically. All hydrogen atoms were generated theoretically and allowed to ride on their respective parent atoms. These data can be obtained free of charge from The Cambridge Crystallographic Data Centre via https://www.ccdc.cam.ac.uk/structures/.

Crystallographic data for **11a**: $C_{50}H_{54}N_2O_2$, M_r = 714.99, 0.160 × 0.090 × 0.070 mm, monoclinic, $P2_1/n$ (no. 14), a = 22.437(7), b = 12.853(4), c = 27.579(9) Å, β = 90.2692(10) °, V = 7953(4) Å^3, Z = 8, Z' = 2, $D_{calcd.}$ = 1.194 gcm^{-3}, θ_{max} = 68.579, T = 93 *K*, 103001 reflections measured, 14529 unique (R_{int} = 0.1074), μ = 0.552 mm^{-1}, T_{max} =0.962, T_{min} = 0.784. The final R_1 and wR_2 were 0.0861 and 0.2560 (all data) for 990 parameters and 0 restraints. The residual electron densities (peak and hole) were 0.76 and -0.31 eÅ^{-3}. CCDC-2486609 contains the supplementary crystallographic data for this paper. The crystal contained three different conformations (Z' = 0.5 for conformation 1 + 1 for conformation 2 + 0.5 for conformation 3). conformation 1 was related by an inversion center at the molecular center, and conformation 3 was also related in the same manner. In the crystal of **11a**, no particularly strong intermolecular interaction was observed. Due to the efficient packing of the molecules, small voids were formed that are too small to accommodate any guest molecules.

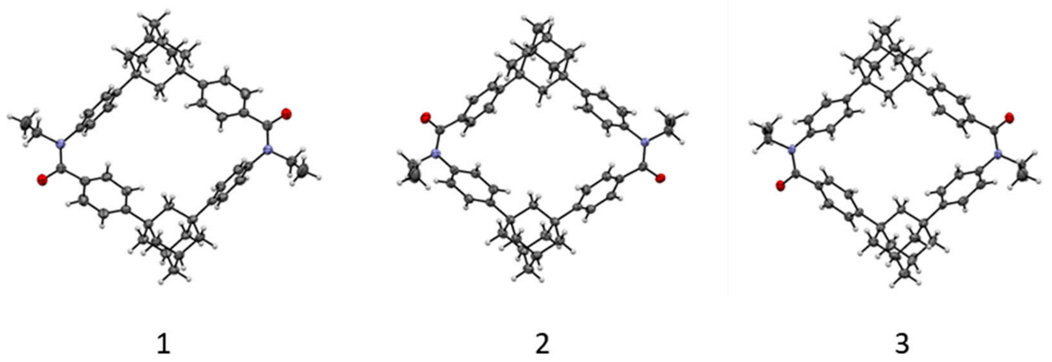

Figure S1. ORTEP diagram of conformation 1, 2 and 3 in a crystal of **11a**. The asymmetric unit contains independent single molecule of conformation 2, and half of a molecule each of conformations 1 and 3. The ellipsoids of non-hydrogen atoms are drawn at the 50% probability level. Colors of atoms: C, gray spheres; O, red spheres; N,

violet spheres; H, light gray spheres.

Crystallographic data for **11b**: $C_{75}H_{81}N_3O_3$, M_r = 1072.48, 0.230 × 0.180 × 0.040 mm, monoclinic, $P2_1/n$ (no. 14), a = 7.3963(3), b = 26.8096(11), c = 31.6104(10) Å, β = 94.535(3) °, V = 6248.5(4) Å^3, Z = 4, Z' = 1, $D_{calcd.}$ = 1.140 gcm^{-3}, θ_{max} = 68.241, T = 93 K, 81397 reflections measured, 11365 unique (R_{int} = 0.1227), μ = 0.527 mm^{-1}, T_{max} = 0. 979, T_{min} = 0. 867. The final R_1 and wR_2 were 0.0849 and 0.2734 (all data) for 761 parameters and 0 restraints. The residual electron densities (peak and hole) were 0.67 and -0.38 eÅ^{-3}. CCDC-2486610 contains the supplementary crystallographic data for this paper. Attempts to refine peaks of residual electron density as guest solvents (chloroform or methanol) were unsuccessful. The data were corrected for disordered electron density through use of the SQUEEZE procedure as implemented in PLATON [34]. A total solvent-accessible void volume of 710 Å^3 with a total electron count of 205 was found in the unit cell. In the crystal of **11b**, no particularly strong intermolecular interaction was observed. The low-symmetry structure of **11b** leads to a relatively loose packing in the crystal lattice, resulting in large voids.

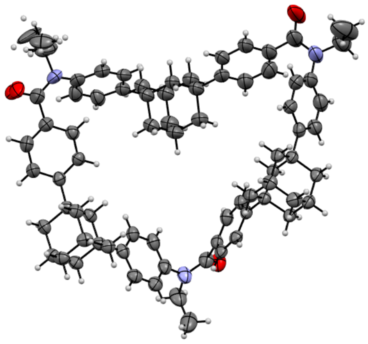

Figure S2. ORTEP diagram of the asymmetric unit in the structure for compound **11b** with displacement ellipsoids at the 50% probability level. Colors of atoms: C, gray spheres; O, red spheres; N, violet spheres; H, light gray spheres.

Crystallographic data for **11c**: $C_{100}H_{108}N_4O_4$, M_r = 1429.98, 0.240 × 0.210 × 0.100 mm, monoclinic, $P2_1/c$ (no. 14), a = 13.63036(19), b = 10.24062(14), c = 28.2361(5) Å, β = 101.3165(15) °, V = 3864.66(10) Å^3, Z = 2, Z' = 0.5, $D_{calcd.}$ = 1.229 gcm^{-3}, θ_{max} = 68.248, T = 93 K, 48824 reflections measured, 7074 unique (R_{int} = 0.0498), μ = 0.568 mm^{-1}, T_{max} = 0. 945, T_{min} = 0. 507. The final R_1 and wR_2 were 0.0731 and 0.2314 (all data) for 489 parameters and 0 restraints. The residual electron densities (peak and hole) were 0.53 and -0.28 eÅ^{-3}. CCDC-2486611 contains the supplementary crystallographic data for this paper. Two symmetrically formed intermolecular interactions, H40···O2* and H85 ···O2^{*1}

(symmetry operations *1: 1−x, 1−y, 1−z), act as the driving force for oligomer formation along the *a*-axis. Furthermore, the O1···H46B*[2] (symmetry operations *2: 1−x, 2−y, 1−z) formed along the <110> surface, leading to three-dimensional packing.

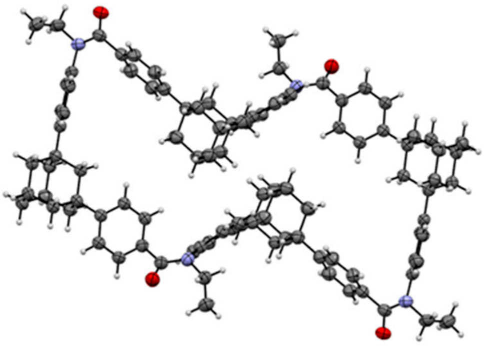

Figure S3. ORTEP diagram of the asymmetric unit in the structure for compound **11c** with displacement ellipsoids at the 50% probability level. Colors of atoms: C, gray spheres; O, red spheres; N, violet spheres; H, light gray spheres.

S2. ^{1}H NMR, ^{13}C NMR spectra

2

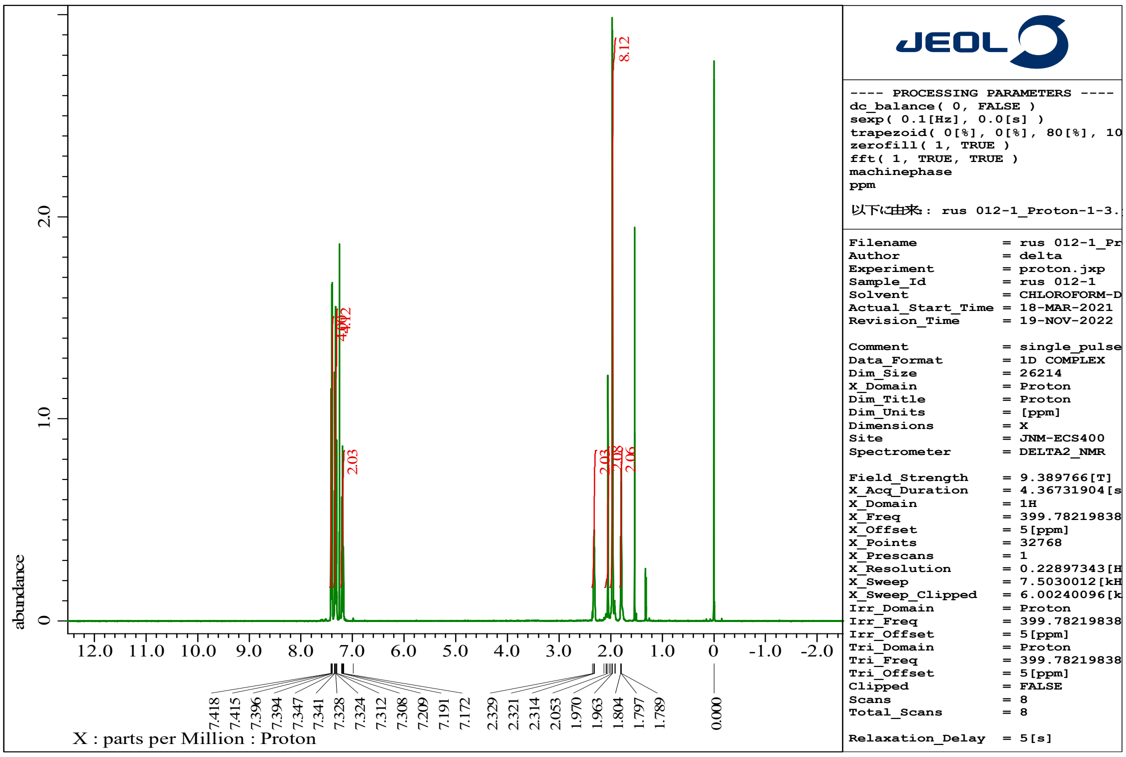

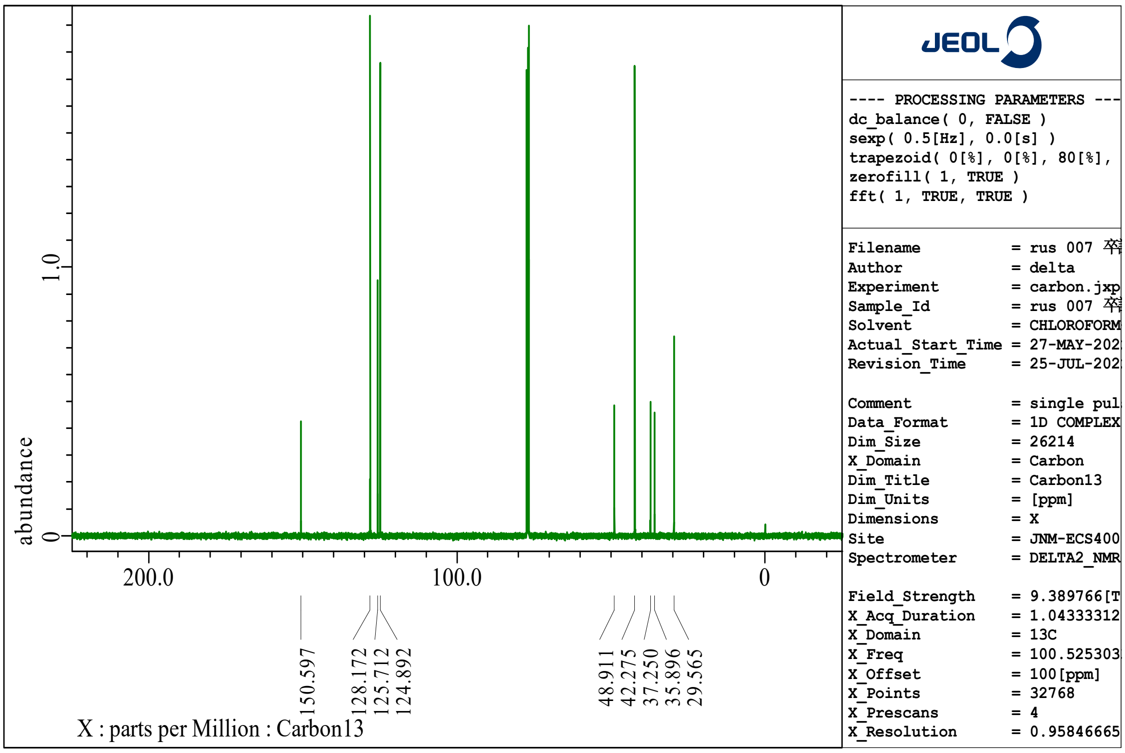

3

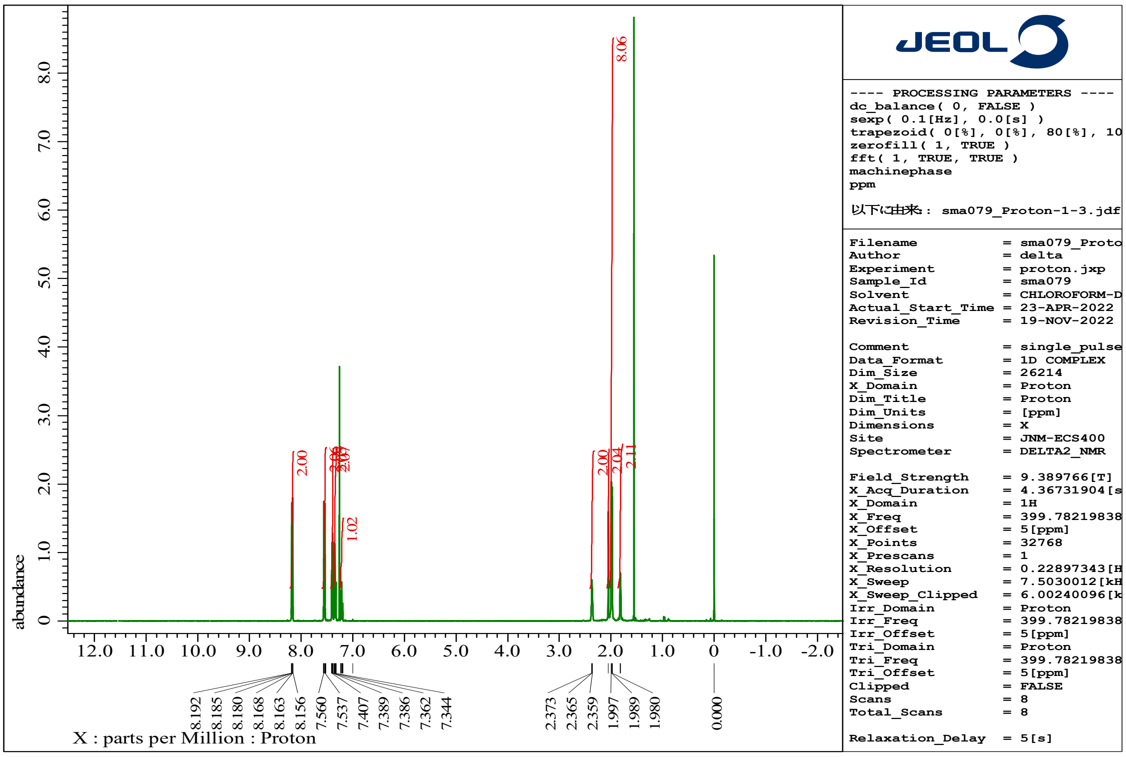

JEOL
---- PROCESSING PARAMETERS ----
dc_balance(0, FALSE)
sexp(0.1[Hz], 0.0[s])
trapezoid(0[%], 0[%], 80[%], 10
zerofill(1, TRUE)
fft(1, TRUE, TRUE)
machinephase
ppm
以下に由来: sma079_Proton-1-3.jdf
Filename = sma079_Proto
Author = delta
Experiment = proton.jxp
Sample_Id = sma079
Solvent = CHLOROFORM-D
Actual_Start_Time = 23-APR-2022
Revision_Time = 19-NOV-2022
Comment = single_pulse
Data_Format = 1D COMPLEX
Dim_Size = 26214
X_Domain = Proton
Dim_Title = Proton
Dim_Units = [ppm]
Dimensions = X
Site = JNM-ECS400
Spectrometer = DELTA2_NMR
Field_Strength = 9.389766[T]
X_Acq_Duration = 4.36731904[s
X_Domain = 1H
X_Freq = 399.78219838
X_Offset = 5[ppm]
X_Points = 32768
X_Prescans = 1
X_Resolution = 0.22897343[H
X_Sweep = 7.5030012[kH
X_Sweep_Clipped = 6.00240096[k
Irr_Domain = Proton
Irr_Freq = 399.78219838
Irr_Offset = 5[ppm]
Tri_Domain = Proton
Tri_Freq = 399.78219838
Tri_Offset = 5[ppm]
Clipped = FALSE
Scans = 8
Total_Scans = 8
Relaxation_Delay = 5[s]
abundance
X : parts per Million : Proton
8.192 8.185 8.180 8.168 8.163 8.156 7.560 7.537 7.407 7.389 7.386 7.362 7.344
2.373 2.365 2.359 1.997 1.989 1.980 0.000
2.00 1.02 2.00 2.04 2.11 8.06

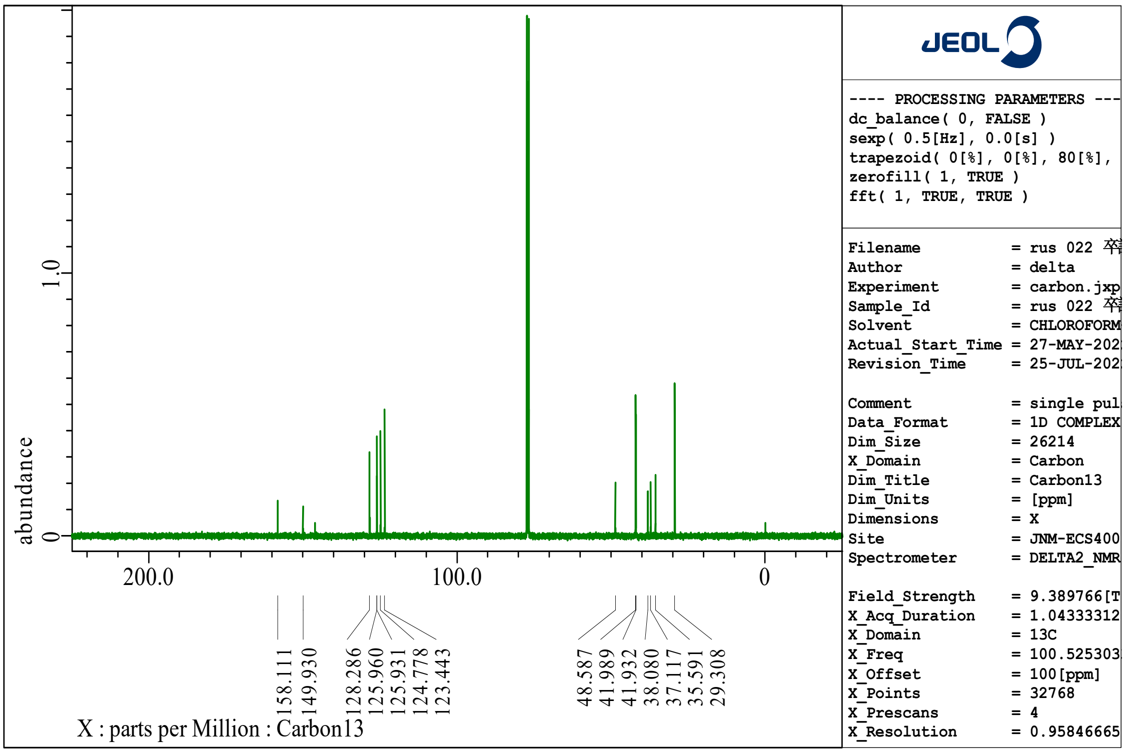

JEOL
---- PROCESSING PARAMETERS ---
dc_balance(0, FALSE)
sexp(0.5[Hz], 0.0[s])
trapezoid(0[%], 0[%], 80[%],
zerofill(1, TRUE)
fft(1, TRUE, TRUE)
Filename = rus 022
Author = delta
Experiment = carbon.jxp
Sample_Id = rus 022
Solvent = CHLOROFORM
Comment = single pul
Data_Format = 1D COMPLEX
Dim_Size = 26214
X_Domain = Carbon
Dim_Title = Carbon13
Dim_Units = [ppm]
Dimensions = X
Site = JNM-ECS400
Spectrometer = DELTA2_NMR
X_Domain = 13C
X_Offset = 100[ppm]
X_Points = 32768
X_Prescans = 4
X_Resolution = 0.95846665
abundance
X : parts per Million : Carbon13
158.111 149.930 128.286 125.960 125.931 124.778 123.443
48.587 41.989 41.932 38.080 37.117 35.591 29.308

4

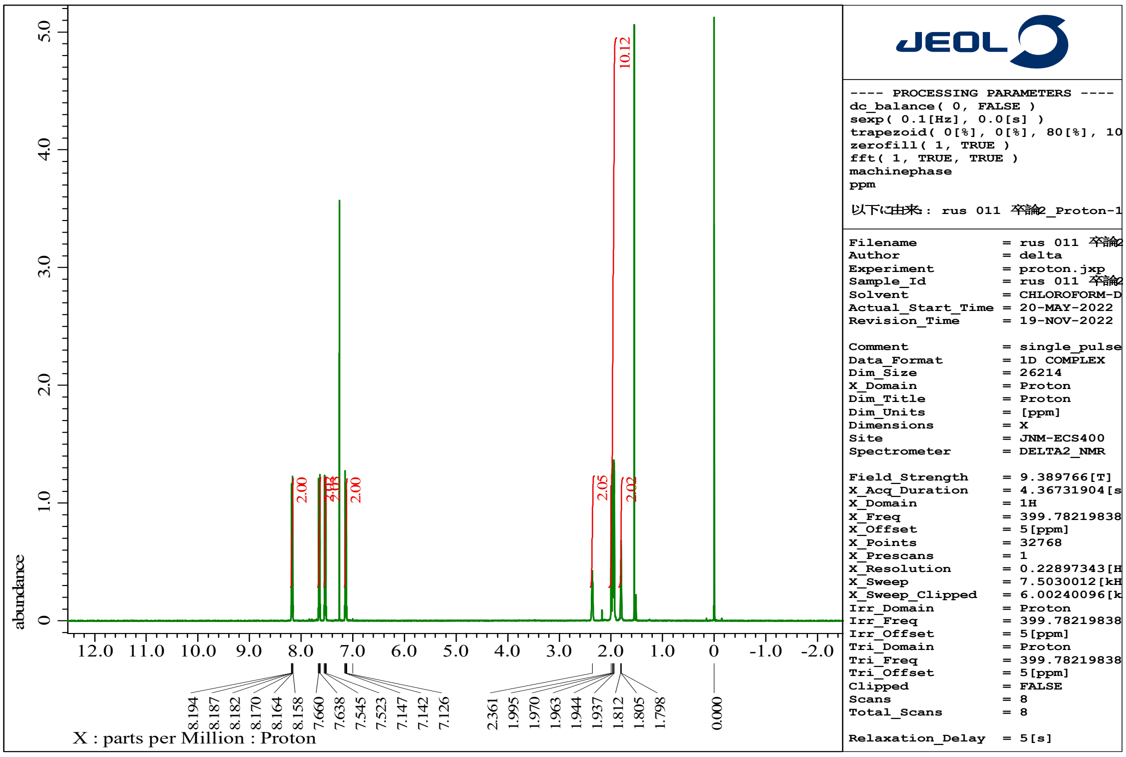

abundance
5.0
4.0
3.0
2.0
1.0
0
2.00
2.00
10.12
2.05
2.02
12.0 11.0 10.0 9.0 8.0 7.0 6.0 5.0 4.0 3.0 2.0 1.0 0 -1.0 -2.0
8.194
8.187
8.182
8.170
8.164
8.158
7.660
7.638
7.545
7.523
7.147
7.142
7.126
2.361
1.995
1.970
1.963
1.944
1.937
1.812
1.805
1.798
0.000
X : parts per Million : Proton
JEOL
---- PROCESSING PARAMETERS ----
dc_balance(0, FALSE)
sexp(0.1[Hz], 0.0[s])
zerofill(1, TRUE)
fft(1, TRUE, TRUE)
machinephase
ppm
Filename = rus 011
Author = delta
Experiment = proton.jxp
Sample_Id = rus 011
Actual_Start_Time = 20-MAY-2022
Revision_Time = 19-NOV-2022
Comment = single_pulse
Data_Format = 1D COMPLEX
Dim_Size = 26214
X_Domain = Proton
Dim_Title = Proton
Dim_Units = [ppm]
Dimensions = X
Site = JNM-ECS400
Spectrometer = DELTA2_NMR
Field_Strength = 9.389766[T]
X_Domain = 1H
X_Offset = 5[ppm]
X_Points = 32768
X_Prescans = 1
Irr_Domain = Proton
Irr_Offset = 5[ppm]
Tri_Domain = Proton
Tri_Offset = 5[ppm]
Clipped = FALSE
Scans = 8
Total_Scans = 8
Relaxation_Delay = 5[s]

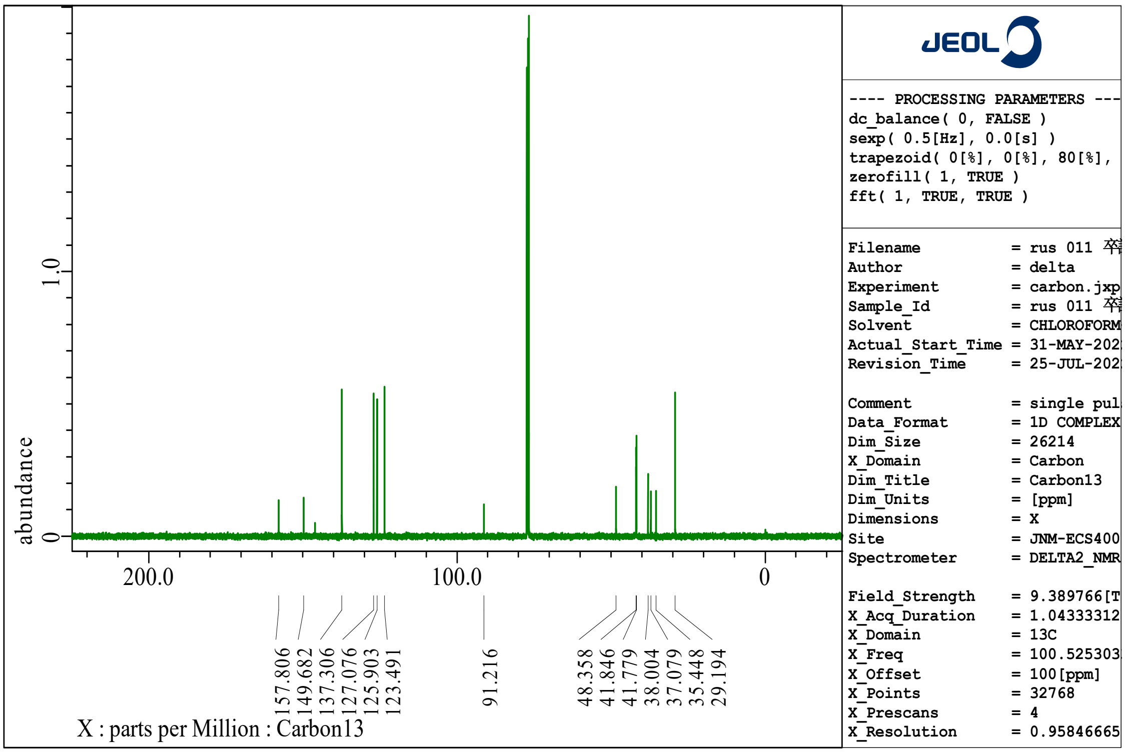

abundance
1.0
0
200.0
100.0
0
157.806
149.682
137.306
127.076
125.903
123.491
91.216
48.358
41.846
41.779
38.004
37.079
35.448
29.194
X : parts per Million : Carbon13
JEOL
---- PROCESSING PARAMETERS ---
dc_balance(0, FALSE)
sexp(0.5[Hz], 0.0[s])
trapezoid(0[%], 0[%], 80[%],
zerofill(1, TRUE)
fft(1, TRUE, TRUE)
Filename = rus 011
Author = delta
Experiment = carbon.jxp
Sample_Id = rus 011
Comment = single pul
Data_Format = 1D COMPLEX
Dim_Size = 26214
X_Domain = Carbon
Dim_Title = Carbon13
Dim_Units = [ppm]
Dimensions = X
Site = JNM-ECS400
Spectrometer = DELTA2_NMR
X_Domain = 13C
X_Offset = 100[ppm]
X_Points = 32768
X_Prescans = 4

5

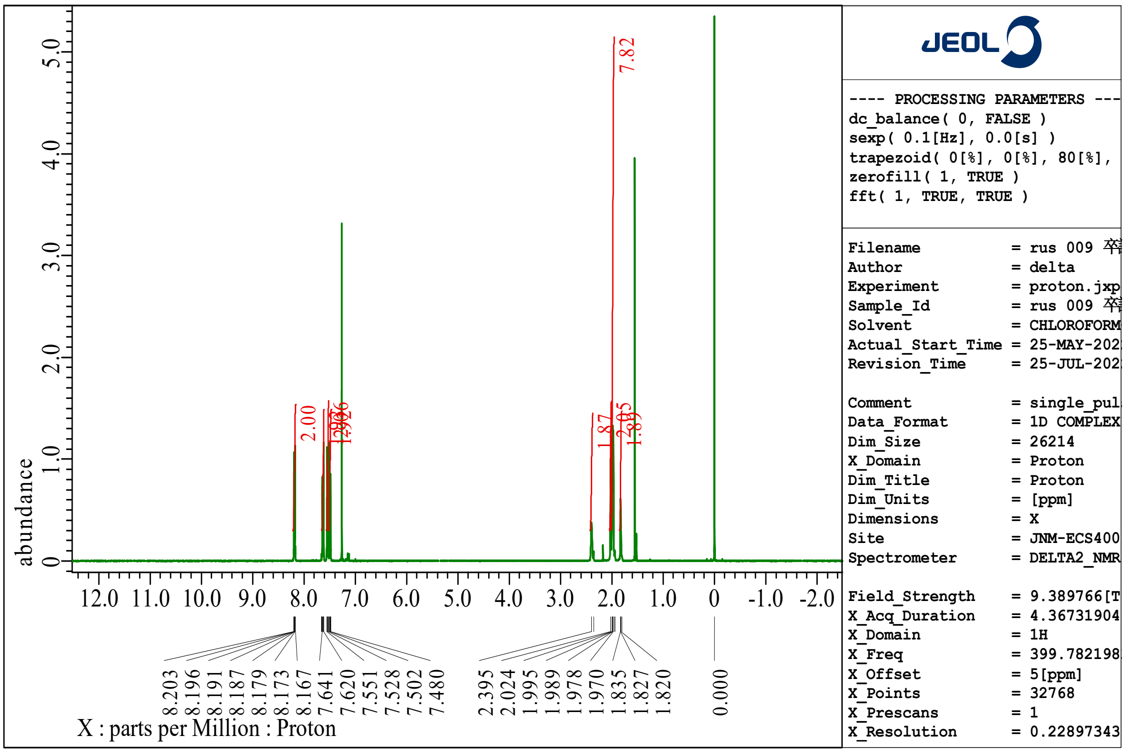

abundance
5.0
4.0
3.0
2.0
1.0
0
7.82
2.00
1.87
2.05
12.0 11.0 10.0 9.0 8.0 7.0 6.0 5.0 4.0 3.0 2.0 1.0 0 -1.0 -2.0
8.203 8.196 8.191 8.187 8.179 8.173 8.167 7.641 7.620 7.551 7.528 7.502 7.480
2.395 2.024 1.995 1.989 1.978 1.970 1.835 1.827 1.820
0.000
X : parts per Million : Proton
JEOL
---- PROCESSING PARAMETERS ---
dc_balance(0, FALSE)
sexp(0.1[Hz], 0.0[s])
trapezoid(0[%], 0[%], 80[%],
zerofill(1, TRUE)
fft(1, TRUE, TRUE)
Filename = rus 009
Author = delta
Experiment = proton.jxp
Sample_Id = rus 009
Solvent = CHLOROFORM
Actual_Start_Time = 25-MAY-202
Revision_Time = 25-JUL-202
Comment = single_pul
Data_Format = 1D COMPLEX
Dim_Size = 26214
X_Domain = Proton
Dim_Title = Proton
Dim_Units = [ppm]
Dimensions = X
Site = JNM-ECS400
Spectrometer = DELTA2_NMR
Field_Strength = 9.389766[T
X_Acq_Duration = 4.36731904
X_Domain = 1H
X_Freq = 399.782198
X_Offset = 5[ppm]
X_Points = 32768
X_Prescans = 1
X_Resolution = 0.22897343

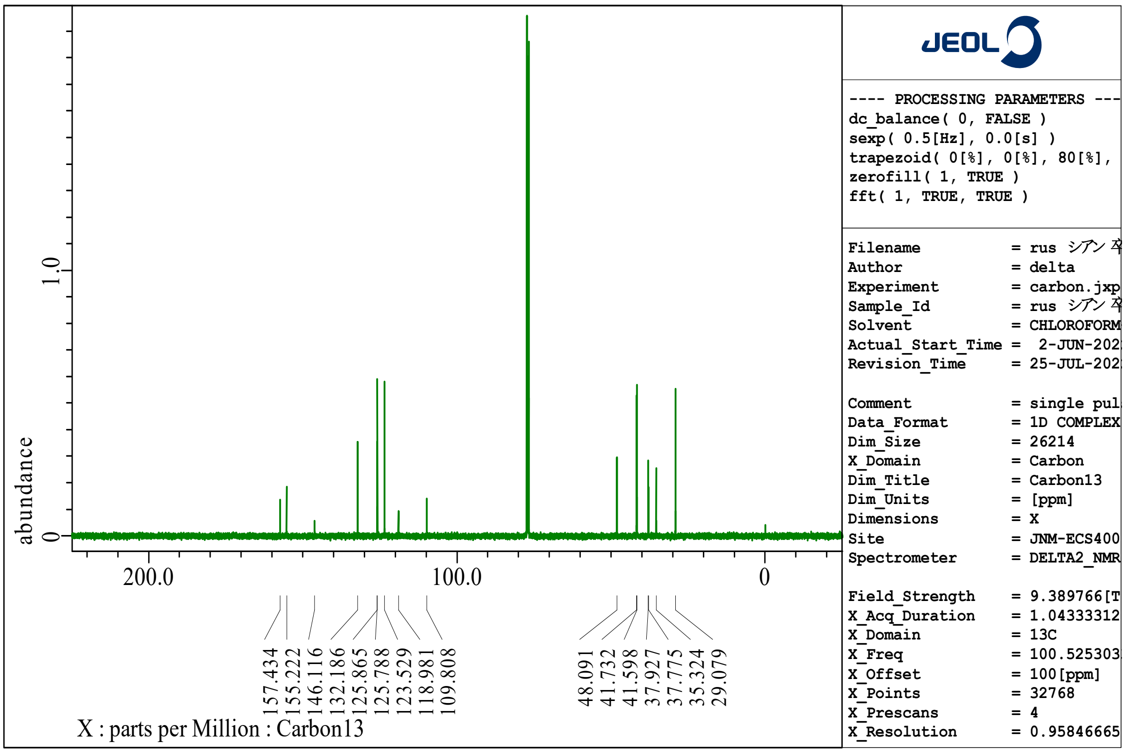

abundance
1.0
0
200.0 100.0 0
157.434 155.222 146.116 132.186 125.865 125.788 123.529 118.981 109.808
48.091 41.732 41.598 37.927 37.775 35.324 29.079
X : parts per Million : Carbon13
JEOL
---- PROCESSING PARAMETERS ---
dc_balance(0, FALSE)
sexp(0.5[Hz], 0.0[s])
trapezoid(0[%], 0[%], 80[%],
zerofill(1, TRUE)
fft(1, TRUE, TRUE)
Filename = rus シアン
Author = delta
Experiment = carbon.jxp
Sample_Id = rus シアン
Solvent = CHLOROFORM
Actual_Start_Time = 2-JUN-202
Revision_Time = 25-JUL-202
Comment = single pul
Data_Format = 1D COMPLEX
Dim_Size = 26214
X_Domain = Carbon
Dim_Title = Carbon13
Dim_Units = [ppm]
Dimensions = X
Site = JNM-ECS400
Spectrometer = DELTA2_NMR
Field_Strength = 9.389766[T
X_Acq_Duration = 1.04333312
X_Domain = 13C
X_Freq = 100.525303
X_Offset = 100[ppm]
X_Points = 32768
X_Prescans = 4
X_Resolution = 0.95846665

6

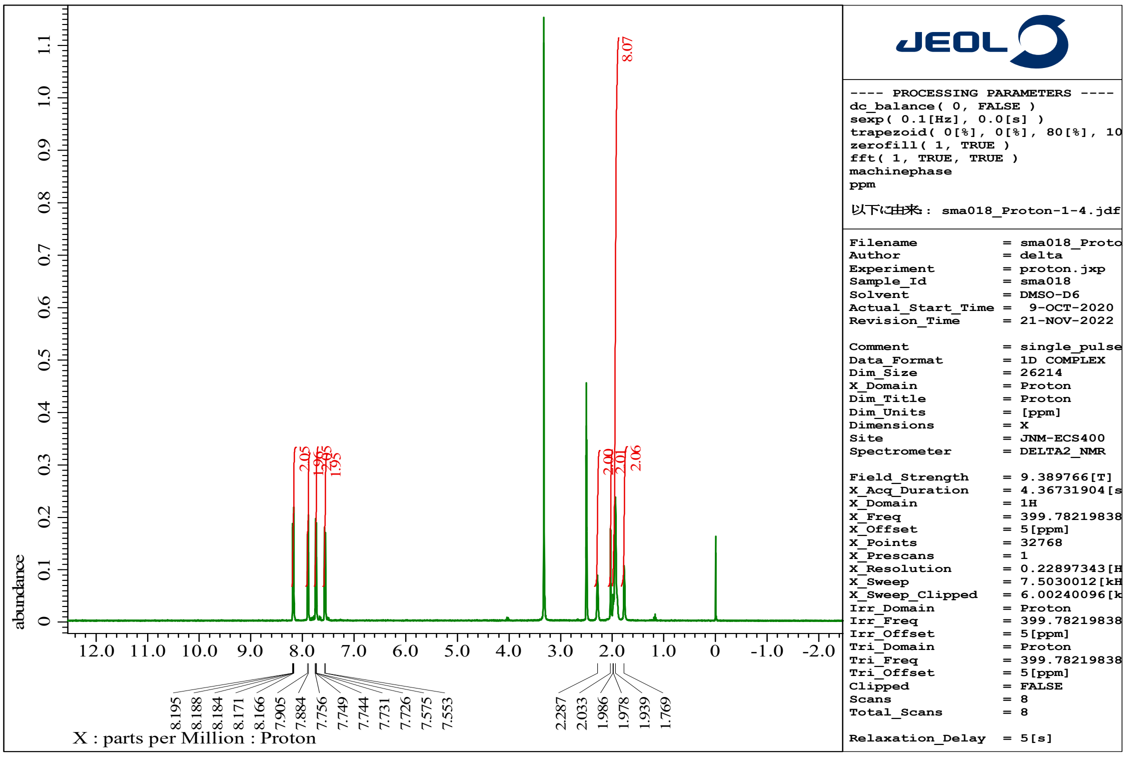

JEOL
---- PROCESSING PARAMETERS ----
dc_balance(0, FALSE)
sexp(0.1[Hz], 0.0[s])
trapezoid(0[%], 0[%], 80[%], 10
zerofill(1, TRUE)
fft(1, TRUE, TRUE)
machinephase
ppm
以下に由来: sma018_Proton-1-4.jdf
Filename = sma018_Proto
Author = delta
Experiment = proton.jxp
Sample_Id = sma018
Solvent = DMSO-D6
Actual_Start_Time = 9-OCT-2020
Revision_Time = 21-NOV-2022
Comment = single_pulse
Data_Format = 1D COMPLEX
Dim_Size = 26214
X_Domain = Proton
Dim_Title = Proton
Dim_Units = [ppm]
Dimensions = X
Site = JNM-ECS400
Spectrometer = DELTA2_NMR
Field_Strength = 9.389766[T]
X_Acq_Duration = 4.36731904[s
X_Domain = 1H
X_Freq = 399.78219838
X_Offset = 5[ppm]
X_Points = 32768
X_Prescans = 1
X_Resolution = 0.22897343[H
X_Sweep = 7.5030012[kH
X_Sweep_Clipped = 6.00240096[k
Irr_Domain = Proton
Irr_Freq = 399.78219838
Irr_Offset = 5[ppm]
Tri_Domain = Proton
Tri_Freq = 399.78219838
Tri_Offset = 5[ppm]
Clipped = FALSE
Scans = 8
Total_Scans = 8
Relaxation_Delay = 5[s]
abundance
X : parts per Million : Proton
8.195 8.188 8.184 8.171 8.166 7.905 7.884 7.756 7.749 7.744 7.731 7.726 7.575 7.553
2.287 2.033 1.986 1.978 1.939 1.769
2.05 1.96 2.05 1.95 2.00 2.01 8.07 2.06

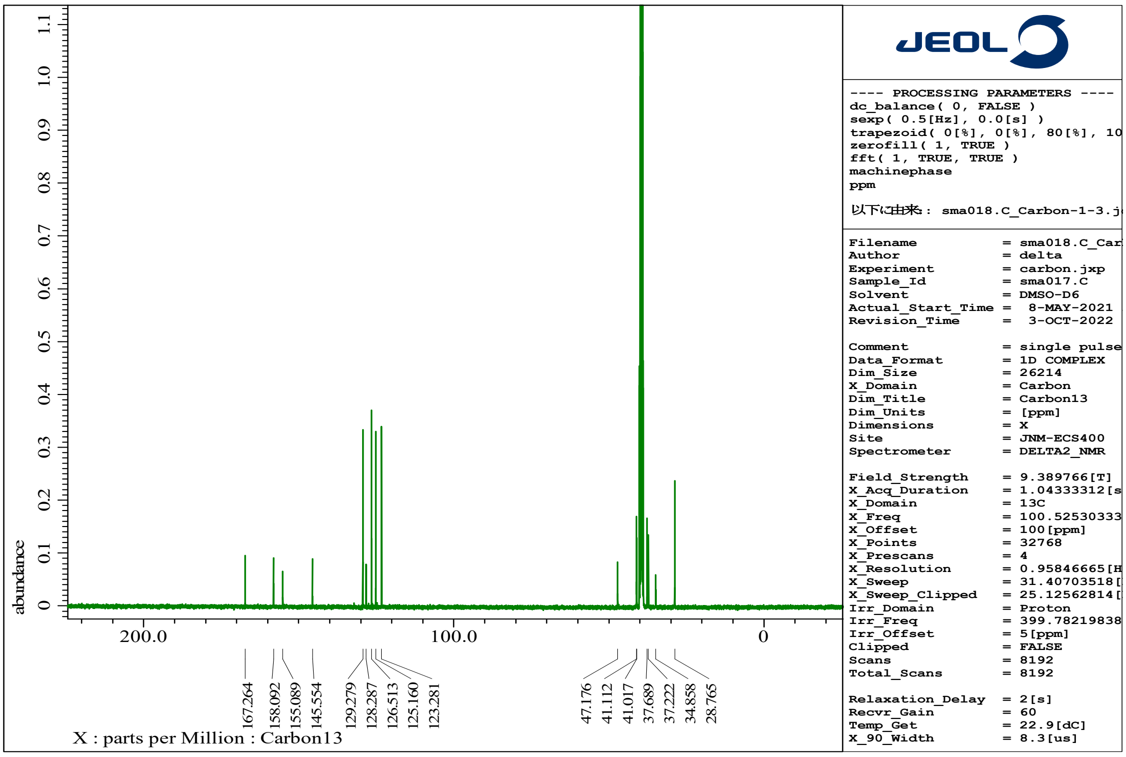

JEOL
---- PROCESSING PARAMETERS ----
dc_balance(0, FALSE)
sexp(0.5[Hz], 0.0[s])
trapezoid(0[%], 0[%], 80[%], 10
zerofill(1, TRUE)
fft(1, TRUE, TRUE)
machinephase
ppm
以下に由来: sma018.C_Carbon-1-3.j
Filename = sma018.C_Car
Author = delta
Experiment = carbon.jxp
Sample_Id = sma017.C
Solvent = DMSO-D6
Actual_Start_Time = 8-MAY-2021
Revision_Time = 3-OCT-2022
Comment = single pulse
Data_Format = 1D COMPLEX
Dim_Size = 26214
X_Domain = Carbon
Dim_Title = Carbon13
Dim_Units = [ppm]
Dimensions = X
Site = JNM-ECS400
Spectrometer = DELTA2_NMR
Field_Strength = 9.389766[T]
X_Acq_Duration = 1.04333312[s
X_Domain = 13C
X_Freq = 100.52530333
X_Offset = 100[ppm]
X_Points = 32768
X_Prescans = 4
X_Resolution = 0.95846665[H
X_Sweep = 31.40703518[
X_Sweep_Clipped = 25.12562814[
Irr_Domain = Proton
Irr_Freq = 399.78219838
Irr_Offset = 5[ppm]
Clipped = FALSE
Scans = 8192
Total_Scans = 8192
Relaxation_Delay = 2[s]
Recvr_Gain = 60
Temp_Get = 22.9[dC]
X_90_Width = 8.3[us]
abundance
X : parts per Million : Carbon13
167.264 158.092 155.089 145.554 129.279 128.287 126.513 125.160 123.281
47.176 41.112 41.017 37.689 37.222 34.858 28.765

7

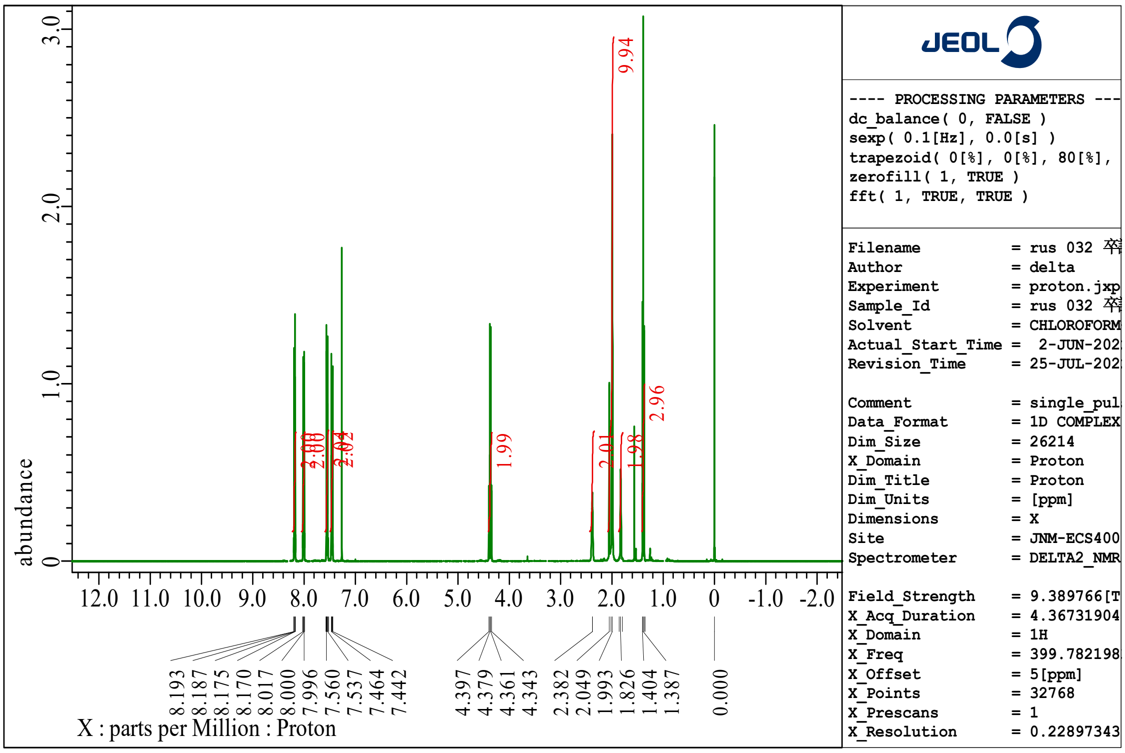
abundance
3.0
2.0
1.0
0
12.0 11.0 10.0 9.0 8.0 7.0 6.0 5.0 4.0 3.0 2.0 1.0 0 -1.0 -2.0
9.94
2.96
2.00
2.00
2.04
2.02
1.99
2.01
1.98
8.193
8.187
8.175
8.170
8.017
8.000
7.996
7.560
7.537
7.464
7.442
4.397
4.379
4.361
4.343
2.382
2.049
1.993
1.826
1.404
1.387
0.000
X : parts per Million : Proton
JEOL
---- PROCESSING PARAMETERS ---
dc_balance(0, FALSE)
sexp(0.1[Hz], 0.0[s])
trapezoid(0[%], 0[%], 80[%],
zerofill(1, TRUE)
fft(1, TRUE, TRUE)
Filename = rus 032
Author = delta
Experiment = proton.jxp
Sample_Id = rus 032
Solvent = CHLOROFORM
Actual_Start_Time = 2-JUN-202
Revision_Time = 25-JUL-202
Comment = single_pul
Data_Format = 1D COMPLEX
Dim_Size = 26214
X_Domain = Proton
Dim_Title = Proton
Dim_Units = [ppm]
Dimensions = X
Site = JNM-ECS400
Spectrometer = DELTA2_NMR
Field_Strength = 9.389766[T
X_Acq_Duration = 4.36731904
X_Domain = 1H
X_Freq = 399.782198
X_Offset = 5[ppm]
X_Points = 32768
X_Prescans = 1
X_Resolution = 0.22897343

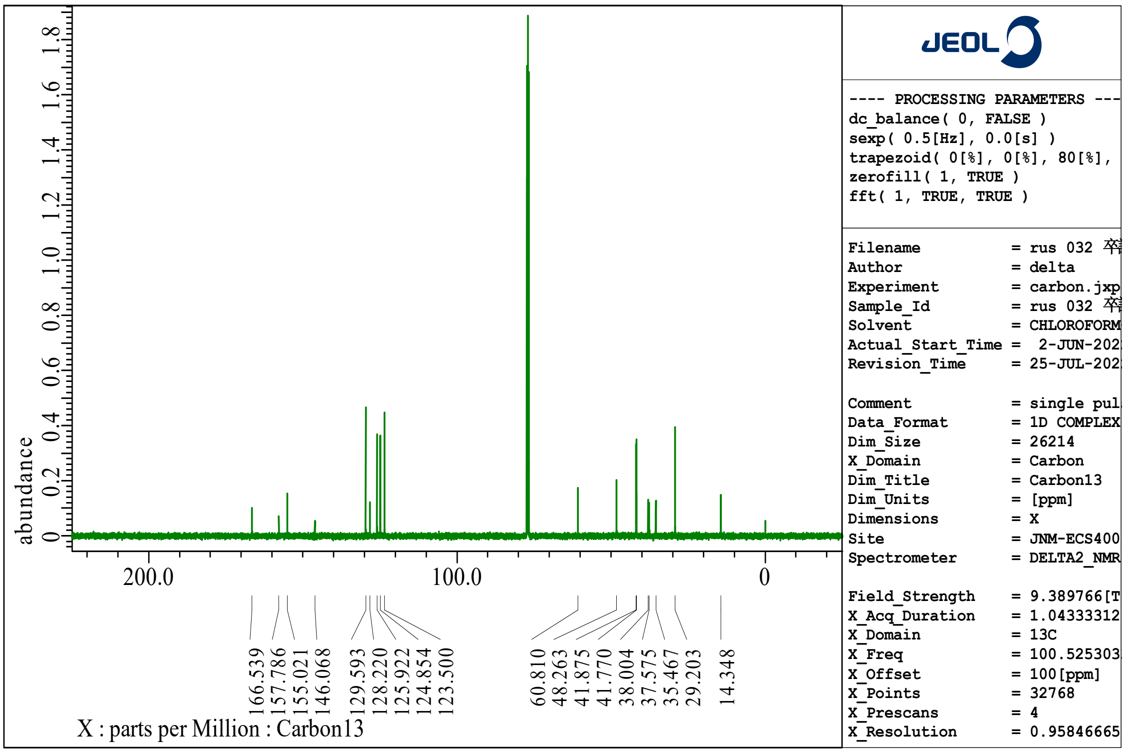
abundance
1.8
1.6
1.4
1.2
1.0
0.8
0.6
0.4
0.2
0
200.0
100.0
0
166.539
157.786
155.021
146.068
129.593
128.220
125.922
124.854
123.500
60.810
48.263
41.875
41.770
38.004
37.575
35.467
29.203
14.348
X : parts per Million : Carbon13
JEOL
---- PROCESSING PARAMETERS ---
dc_balance(0, FALSE)
sexp(0.5[Hz], 0.0[s])
trapezoid(0[%], 0[%], 80[%],
zerofill(1, TRUE)
fft(1, TRUE, TRUE)
Filename = rus 032
Author = delta
Experiment = carbon.jxp
Sample_Id = rus 032
Solvent = CHLOROFORM
Actual_Start_Time = 2-JUN-202
Revision_Time = 25-JUL-202
Comment = single pul
Data_Format = 1D COMPLEX
Dim_Size = 26214
X_Domain = Carbon
Dim_Title = Carbon13
Dim_Units = [ppm]
Dimensions = X
Site = JNM-ECS400
Spectrometer = DELTA2_NMR
Field_Strength = 9.389766[T
X_Acq_Duration = 1.04333312
X_Domain = 13C
X_Freq = 100.525303
X_Offset = 100[ppm]
X_Points = 32768
X_Prescans = 4
X_Resolution = 0.95846665

8

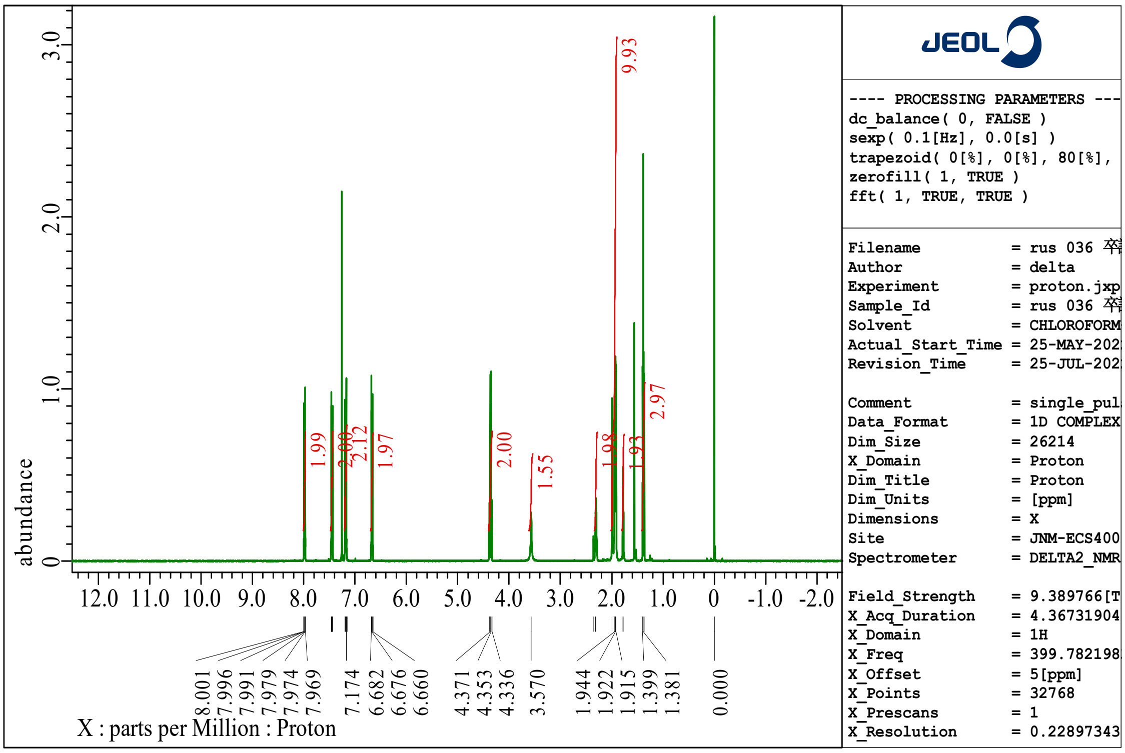

abundance
3.0
2.0
1.0
0
1.99
2.08
2.12
1.97
2.00
1.55
1.98
9.93
1.93
2.97
12.0 11.0 10.0 9.0 8.0 7.0 6.0 5.0 4.0 3.0 2.0 1.0 0 -1.0 -2.0
8.001
7.996
7.991
7.979
7.974
7.969
7.174
6.682
6.676
6.660
4.371
4.353
4.336
3.570
1.944
1.922
1.915
1.399
1.381
0.000
X : parts per Million : Proton
JEOL
---- PROCESSING PARAMETERS ---
dc_balance(0, FALSE)
sexp(0.1[Hz], 0.0[s])
trapezoid(0[%], 0[%], 80[%],
zerofill(1, TRUE)
fft(1, TRUE, TRUE)
Filename = rus 036
Author = delta
Experiment = proton.jxp
Sample_Id = rus 036
Solvent = CHLOROFORM
Actual_Start_Time = 25-MAY-202
Revision_Time = 25-JUL-202
Comment = single_pul
Data_Format = 1D COMPLEX
Dim_Size = 26214
X_Domain = Proton
Dim_Title = Proton
Dim_Units = [ppm]
Dimensions = X
Site = JNM-ECS400
Spectrometer = DELTA2_NMR
Field_Strength = 9.389766[T
X_Acq_Duration = 4.36731904
X_Domain = 1H
X_Freq = 399.782198
X_Offset = 5[ppm]
X_Points = 32768
X_Prescans = 1
X_Resolution = 0.22897343

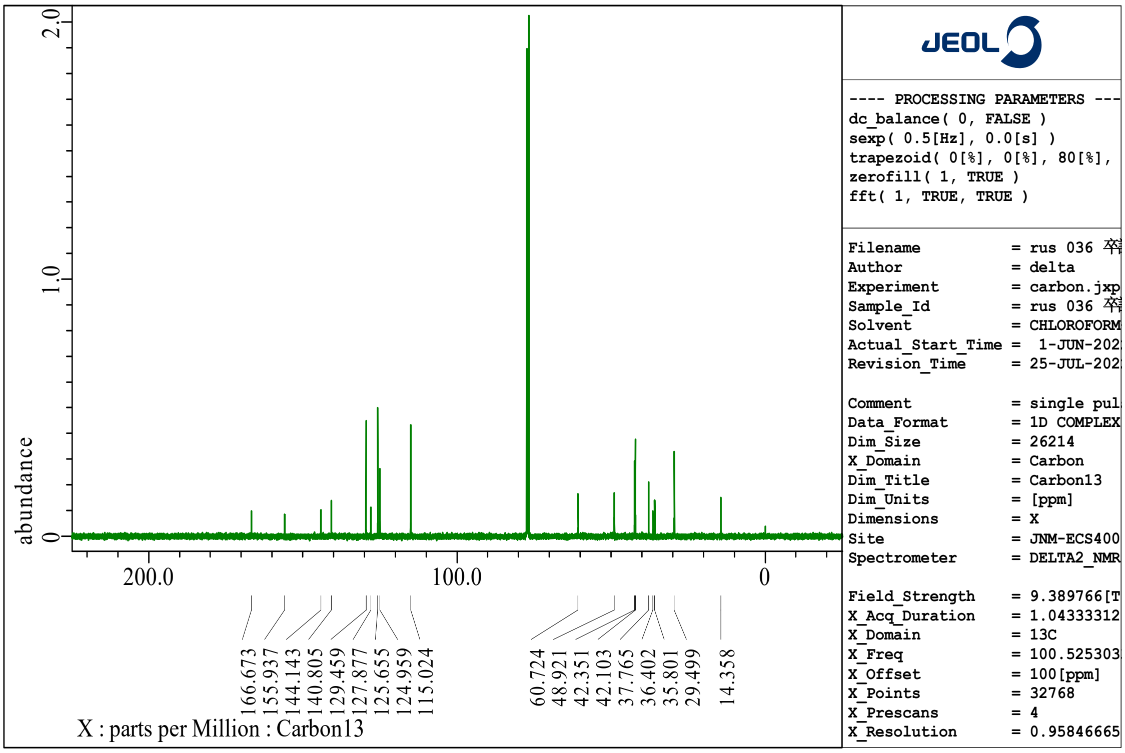

abundance
2.0
1.0
0
200.0
100.0
0
166.673
155.937
144.143
140.805
129.459
127.877
125.655
124.959
115.024
60.724
48.921
42.351
42.103
37.765
36.402
35.801
29.499
14.358
X : parts per Million : Carbon13
JEOL
---- PROCESSING PARAMETERS ---
dc_balance(0, FALSE)
sexp(0.5[Hz], 0.0[s])
trapezoid(0[%], 0[%], 80[%],
zerofill(1, TRUE)
fft(1, TRUE, TRUE)
Filename = rus 036
Author = delta
Experiment = carbon.jxp
Sample_Id = rus 036
Solvent = CHLOROFORM
Actual_Start_Time = 1-JUN-202
Revision_Time = 25-JUL-202
Comment = single pul
Data_Format = 1D COMPLEX
Dim_Size = 26214
X_Domain = Carbon
Dim_Title = Carbon13
Dim_Units = [ppm]
Dimensions = X
Site = JNM-ECS400
Spectrometer = DELTA2_NMR
Field_Strength = 9.389766[T
X_Acq_Duration = 1.04333312
X_Domain = 13C
X_Freq = 100.525303
X_Offset = 100[ppm]
X_Points = 32768
X_Prescans = 4
X_Resolution = 0.95846665

9

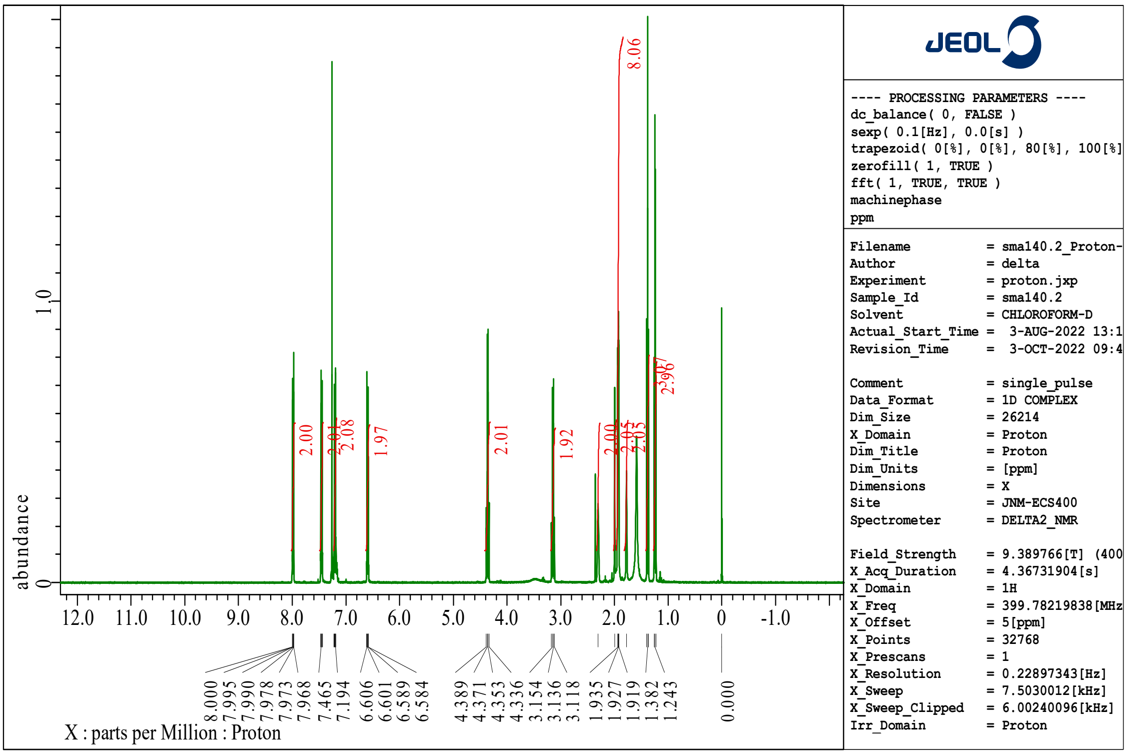
JEOL
---- PROCESSING PARAMETERS ----
dc_balance(0, FALSE)
sexp(0.1[Hz], 0.0[s])
trapezoid(0[%], 0[%], 80[%], 100[%]
zerofill(1, TRUE)
fft(1, TRUE, TRUE)
machinephase
ppm
Filename = sma140.2_Proton-
Author = delta
Experiment = proton.jxp
Sample_Id = sma140.2
Solvent = CHLOROFORM-D
Actual_Start_Time = 3-AUG-2022 13:1
Revision_Time = 3-OCT-2022 09:4
Comment = single_pulse
Data_Format = 1D COMPLEX
Dim_Size = 26214
X_Domain = Proton
Dim_Title = Proton
Dim_Units = [ppm]
Dimensions = X
Site = JNM-ECS400
Spectrometer = DELTA2_NMR
Field_Strength = 9.389766[T] (400
X_Acq_Duration = 4.36731904[s]
X_Domain = 1H
X_Freq = 399.78219838[MHz
X_Offset = 5[ppm]
X_Points = 32768
X_Prescans = 1
X_Resolution = 0.22897343[Hz]
X_Sweep = 7.5030012[kHz]
X_Sweep_Clipped = 6.00240096[kHz]
Irr_Domain = Proton
abundance
X : parts per Million : Proton
8.000 7.995 7.990 7.978 7.973 7.968 7.465 7.194 6.606 6.601 6.589 6.584 4.389 4.371 4.353 4.336 3.154 3.136 3.118 1.935 1.927 1.919 1.382 1.243 0.000
2.00 2.01 2.08 1.97 2.01 1.92 2.00 2.05 2.05 8.06 2.96

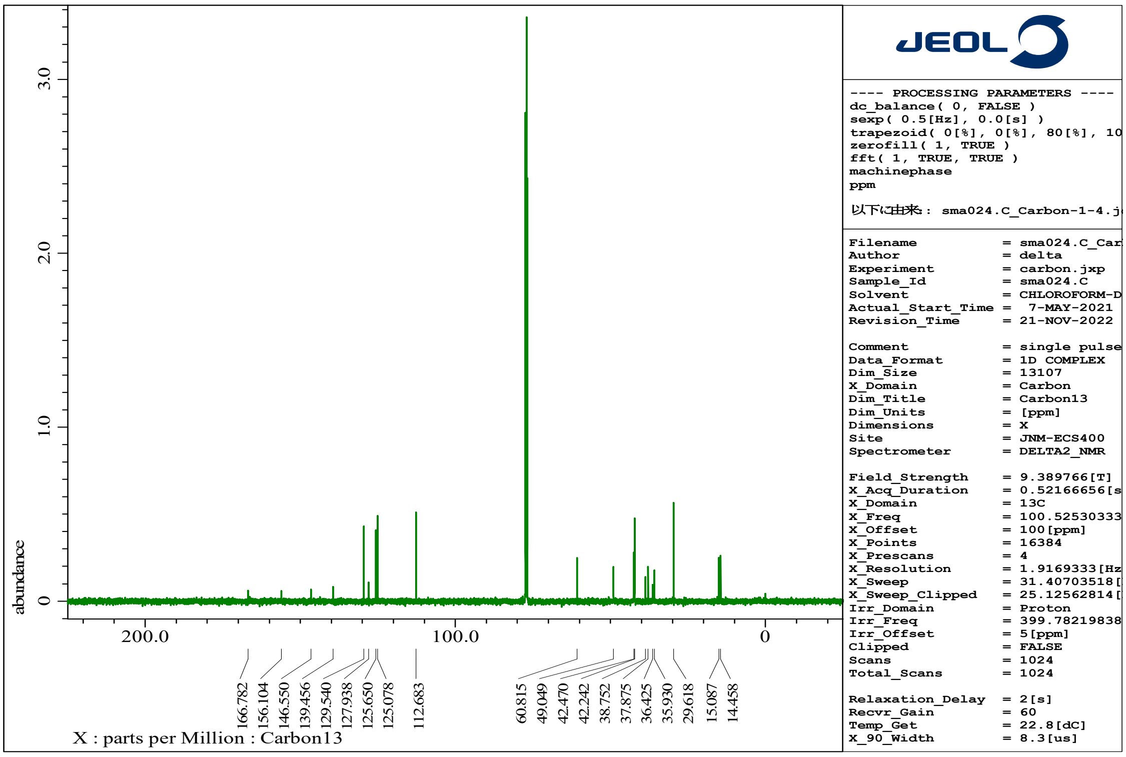
JEOL
---- PROCESSING PARAMETERS ----
dc_balance(0, FALSE)
sexp(0.5[Hz], 0.0[s])
trapezoid(0[%], 0[%], 80[%], 10
zerofill(1, TRUE)
fft(1, TRUE, TRUE)
machinephase
ppm
以下に由来: sma024.C_Carbon-1-4.j
Filename = sma024.C_Car
Author = delta
Experiment = carbon.jxp
Sample_Id = sma024.C
Solvent = CHLOROFORM-D
Actual_Start_Time = 7-MAY-2021
Revision_Time = 21-NOV-2022
Comment = single pulse
Data_Format = 1D COMPLEX
Dim_Size = 13107
X_Domain = Carbon
Dim_Title = Carbon13
Dim_Units = [ppm]
Dimensions = X
Site = JNM-ECS400
Spectrometer = DELTA2_NMR
Field_Strength = 9.389766[T]
X_Acq_Duration = 0.52166656[s
X_Domain = 13C
X_Freq = 100.52530333
X_Offset = 100[ppm]
X_Points = 16384
X_Prescans = 4
X_Resolution = 1.9169333[Hz
X_Sweep = 31.40703518[
X_Sweep_Clipped = 25.12562814[
Irr_Domain = Proton
Irr_Freq = 399.78219838
Irr_Offset = 5[ppm]
Clipped = FALSE
Scans = 1024
Total_Scans = 1024
Relaxation_Delay = 2[s]
Recvr_Gain = 60
Temp_Get = 22.8[dC]
X_90_Width = 8.3[us]
abundance
X : parts per Million : Carbon13
166.782 156.104 146.550 139.456 129.540 127.938 125.650 125.078 112.683 60.815 49.049 42.470 42.242 38.752 37.875 36.425 35.930 29.618 15.087 14.458

10

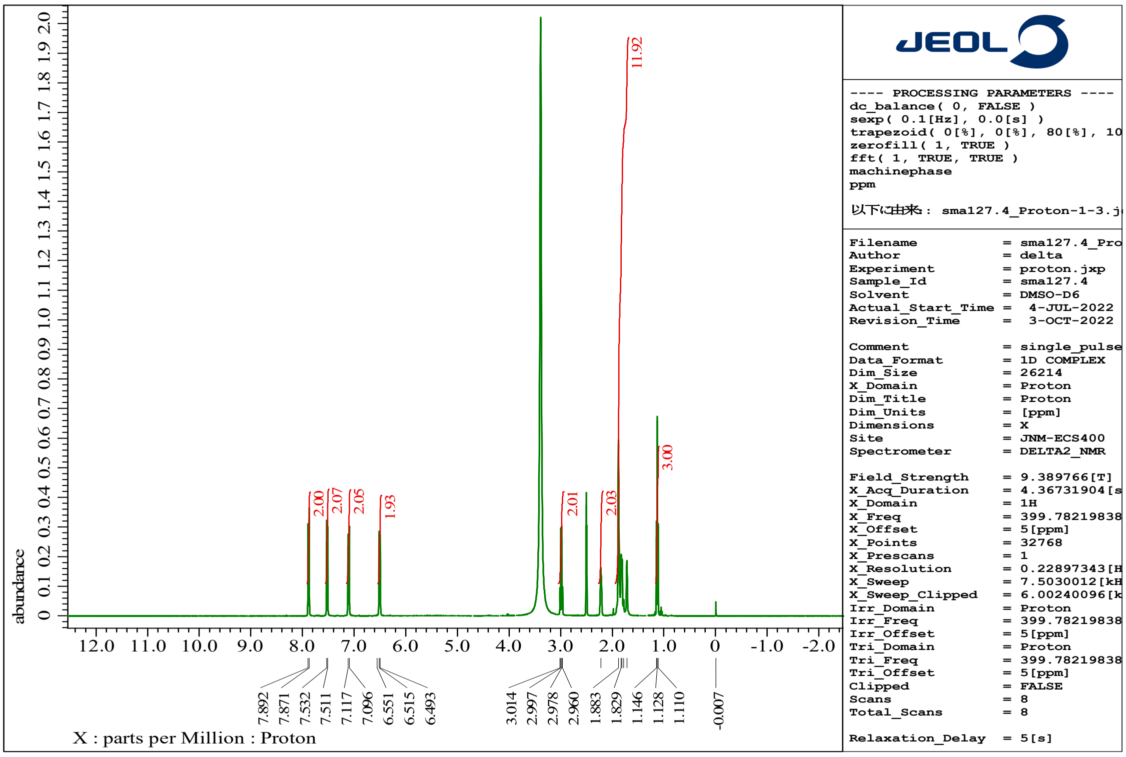

abundance
0 0.1 0.2 0.3 0.4 0.5 0.6 0.7 0.8 0.9 1.0 1.1 1.2 1.3 1.4 1.5 1.6 1.7 1.8 1.9 2.0
2.00
2.07
2.05
1.93
2.01
2.03
11.92
3.00
12.0 11.0 10.0 9.0 8.0 7.0 6.0 5.0 4.0 3.0 2.0 1.0 0 -1.0 -2.0
7.892
7.871
7.532
7.511
7.117
7.096
6.551
6.515
6.493
3.014
2.997
2.978
2.960
1.883
1.829
1.146
1.128
1.110
-0.007
X : parts per Million : Proton
JEOL
---- PROCESSING PARAMETERS ----
dc_balance(0, FALSE)
sexp(0.1[Hz], 0.0[s])
trapezoid(0[%], 0[%], 80[%], 10
zerofill(1, TRUE)
fft(1, TRUE, TRUE)
machinephase
ppm
以下に由来: sma127.4_Proton-1-3.j
Filename = sma127.4_Pro
Author = delta
Experiment = proton.jxp
Sample_Id = sma127.4
Solvent = DMSO-D6
Actual_Start_Time = 4-JUL-2022
Revision_Time = 3-OCT-2022
Comment = single_pulse
Data_Format = 1D COMPLEX
Dim_Size = 26214
X_Domain = Proton
Dim_Title = Proton
Dim_Units = [ppm]
Dimensions = X
Site = JNM-ECS400
Spectrometer = DELTA2_NMR
Field_Strength = 9.389766[T]
X_Acq_Duration = 4.36731904[s
X_Domain = 1H
X_Freq = 399.78219838
X_Offset = 5[ppm]
X_Points = 32768
X_Prescans = 1
X_Resolution = 0.22897343[H
X_Sweep = 7.5030012[kH
X_Sweep_Clipped = 6.00240096[k
Irr_Domain = Proton
Irr_Freq = 399.78219838
Irr_Offset = 5[ppm]
Tri_Domain = Proton
Tri_Freq = 399.78219838
Tri_Offset = 5[ppm]
Clipped = FALSE
Scans = 8
Total_Scans = 8
Relaxation_Delay = 5[s]

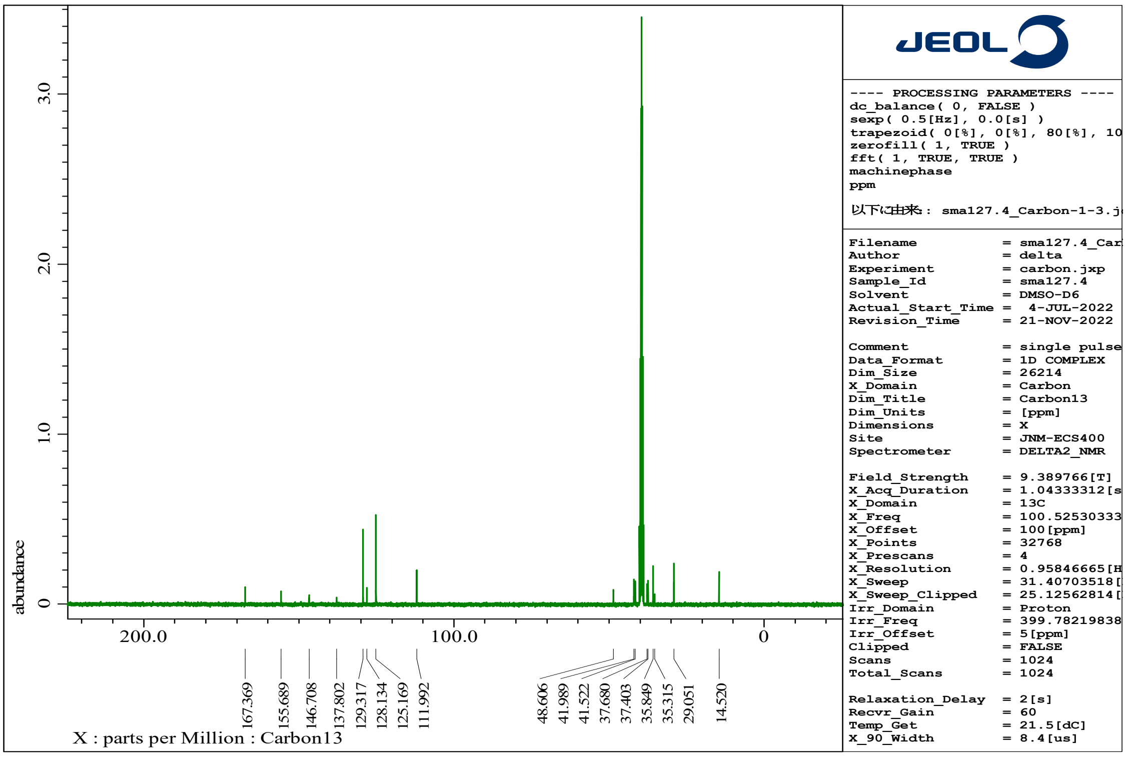

abundance
0 1.0 2.0 3.0
200.0 100.0 0
167.369
155.689
146.708
137.802
129.317
128.134
125.169
111.992
48.606
41.989
41.522
37.680
37.403
35.849
35.315
29.051
14.520
X : parts per Million : Carbon13
JEOL
---- PROCESSING PARAMETERS ----
dc_balance(0, FALSE)
sexp(0.5[Hz], 0.0[s])
trapezoid(0[%], 0[%], 80[%], 10
zerofill(1, TRUE)
fft(1, TRUE, TRUE)
machinephase
ppm
以下に由来: sma127.4_Carbon-1-3.j
Filename = sma127.4_Car
Author = delta
Experiment = carbon.jxp
Sample_Id = sma127.4
Solvent = DMSO-D6
Actual_Start_Time = 4-JUL-2022
Revision_Time = 21-NOV-2022
Comment = single pulse
Data_Format = 1D COMPLEX
Dim_Size = 26214
X_Domain = Carbon
Dim_Title = Carbon13
Dim_Units = [ppm]
Dimensions = X
Site = JNM-ECS400
Spectrometer = DELTA2_NMR
Field_Strength = 9.389766[T]
X_Acq_Duration = 1.04333312[s
X_Domain = 13C
X_Freq = 100.52530333
X_Offset = 100[ppm]
X_Points = 32768
X_Prescans = 4
X_Resolution = 0.95846665[H
X_Sweep = 31.40703518[
X_Sweep_Clipped = 25.12562814[
Irr_Domain = Proton
Irr_Freq = 399.78219838
Irr_Offset = 5[ppm]
Clipped = FALSE
Scans = 1024
Total_Scans = 1024
Relaxation_Delay = 2[s]
Recvr_Gain = 60
Temp_Get = 21.5[dC]
X_90_Width = 8.4[us]

11a

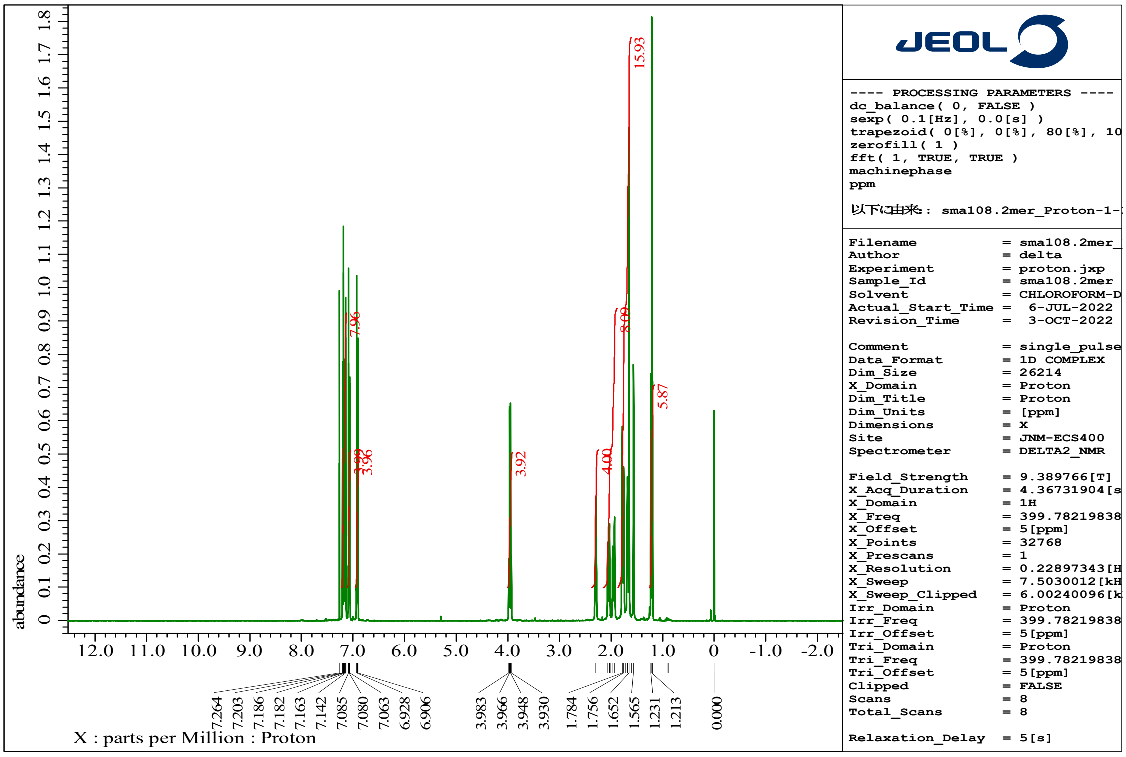

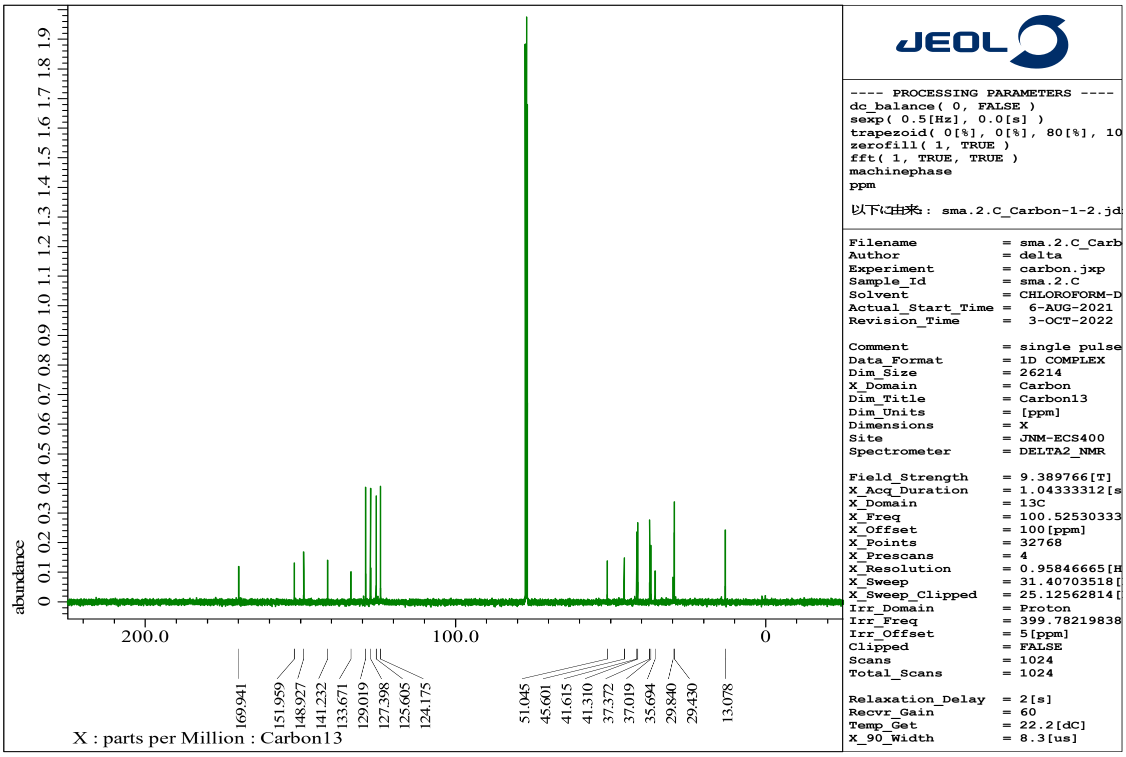

11b

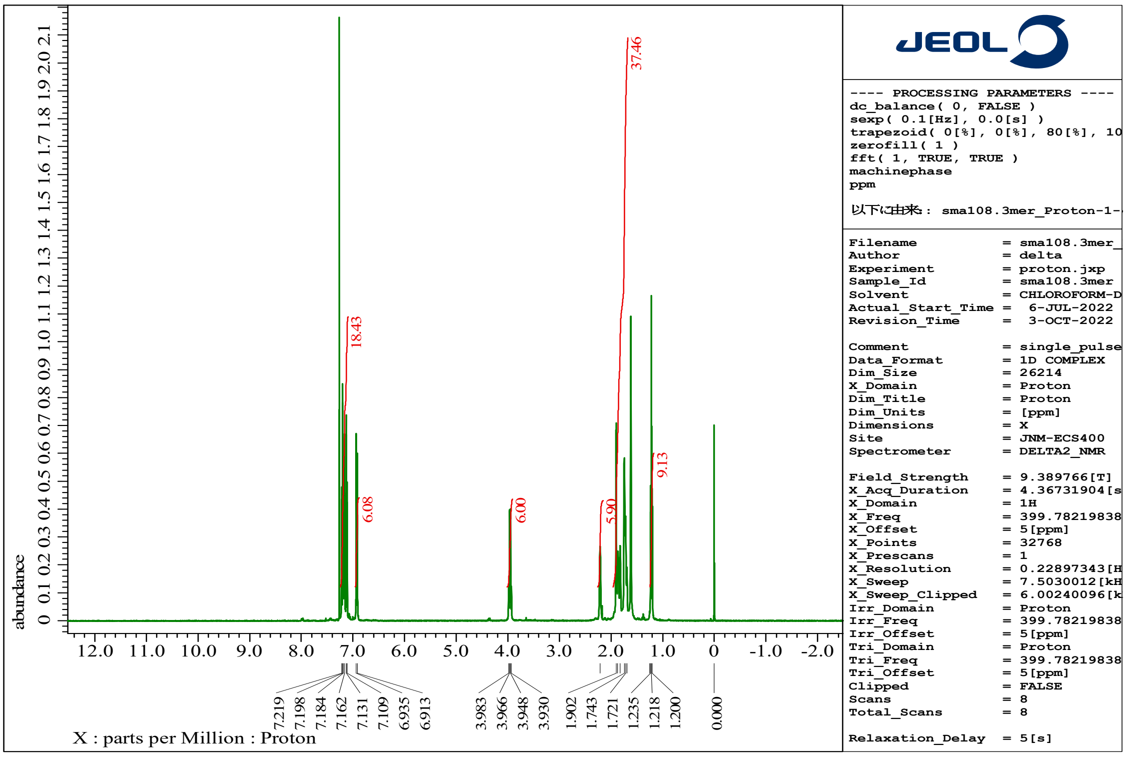
JEOL
---- PROCESSING PARAMETERS ----
dc_balance(0, FALSE)
sexp(0.1[Hz], 0.0[s])
trapezoid(0[%], 0[%], 80[%], 10
zerofill(1)
fft(1, TRUE, TRUE)
machinephase
ppm
以下に由来： sma108.3mer_Proton-1-
Filename = sma108.3mer_
Author = delta
Experiment = proton.jxp
Sample_Id = sma108.3mer
Solvent = CHLOROFORM-D
Actual_Start_Time = 6-JUL-2022
Revision_Time = 3-OCT-2022
Comment = single_pulse
Data_Format = 1D COMPLEX
Dim_Size = 26214
X_Domain = Proton
Dim_Title = Proton
Dim_Units = [ppm]
Dimensions = X
Site = JNM-ECS400
Spectrometer = DELTA2_NMR
Field_Strength = 9.389766[T]
X_Acq_Duration = 4.36731904[s
X_Domain = 1H
X_Freq = 399.78219838
X_Offset = 5[ppm]
X_Points = 32768
X_Prescans = 1
X_Resolution = 0.22897343[H
X_Sweep = 7.5030012[kH
X_Sweep_Clipped = 6.00240096[k
Irr_Domain = Proton
Irr_Freq = 399.78219838
Irr_Offset = 5[ppm]
Tri_Domain = Proton
Tri_Freq = 399.78219838
Tri_Offset = 5[ppm]
Clipped = FALSE
Scans = 8
Total_Scans = 8
Relaxation_Delay = 5[s]
abundance
0 0.1 0.2 0.3 0.4 0.5 0.6 0.7 0.8 0.9 1.0 1.1 1.2 1.3 1.4 1.5 1.6 1.7 1.8 1.9 2.0 2.1
12.0 11.0 10.0 9.0 8.0 7.0 6.0 5.0 4.0 3.0 2.0 1.0 0 -1.0 -2.0
18.43
6.08
6.00
5.90
37.46
9.13
7.219
7.198
7.184
7.162
7.131
7.109
6.935
6.913
3.983
3.966
3.948
3.930
1.902
1.743
1.721
1.235
1.218
1.200
0.000
X : parts per Million : Proton

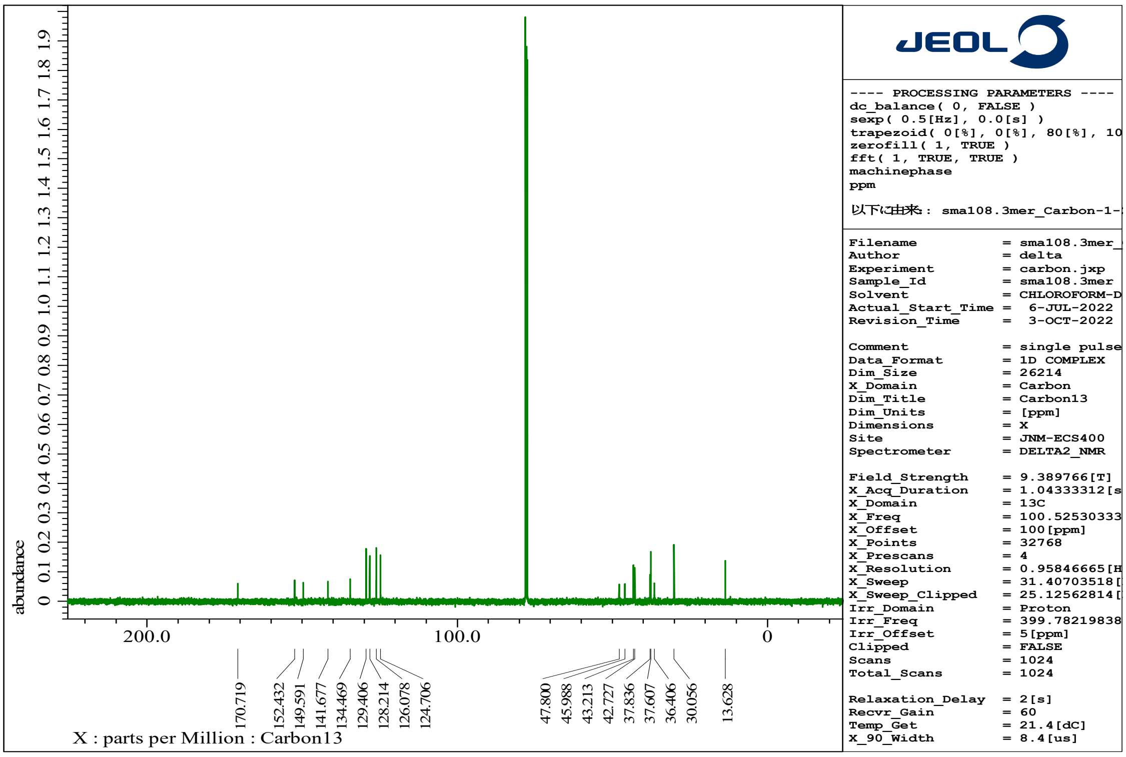
JEOL
---- PROCESSING PARAMETERS ----
dc_balance(0, FALSE)
sexp(0.5[Hz], 0.0[s])
trapezoid(0[%], 0[%], 80[%], 10
zerofill(1, TRUE)
fft(1, TRUE, TRUE)
machinephase
ppm
以下に由来： sma108.3mer_Carbon-1-
Filename = sma108.3mer_
Author = delta
Experiment = carbon.jxp
Sample_Id = sma108.3mer
Solvent = CHLOROFORM-D
Actual_Start_Time = 6-JUL-2022
Revision_Time = 3-OCT-2022
Comment = single pulse
Data_Format = 1D COMPLEX
Dim_Size = 26214
X_Domain = Carbon
Dim_Title = Carbon13
Dim_Units = [ppm]
Dimensions = X
Site = JNM-ECS400
Spectrometer = DELTA2_NMR
Field_Strength = 9.389766[T]
X_Acq_Duration = 1.04333312[s
X_Domain = 13C
X_Freq = 100.52530333
X_Offset = 100[ppm]
X_Points = 32768
X_Prescans = 4
X_Resolution = 0.95846665[H
X_Sweep = 31.40703518[
X_Sweep_Clipped = 25.12562814[
Irr_Domain = Proton
Irr_Freq = 399.78219838
Irr_Offset = 5[ppm]
Clipped = FALSE
Scans = 1024
Total_Scans = 1024
Relaxation_Delay = 2[s]
Recvr_Gain = 60
Temp_Get = 21.4[dC]
X_90_Width = 8.4[us]
abundance
0 0.1 0.2 0.3 0.4 0.5 0.6 0.7 0.8 0.9 1.0 1.1 1.2 1.3 1.4 1.5 1.6 1.7 1.8 1.9
200.0 100.0 0
170.719
152.432
149.591
141.677
134.469
129.406
128.214
126.078
124.706
47.800
45.988
43.213
42.727
37.836
37.607
36.406
30.056
13.628
X : parts per Million : Carbon13

11c

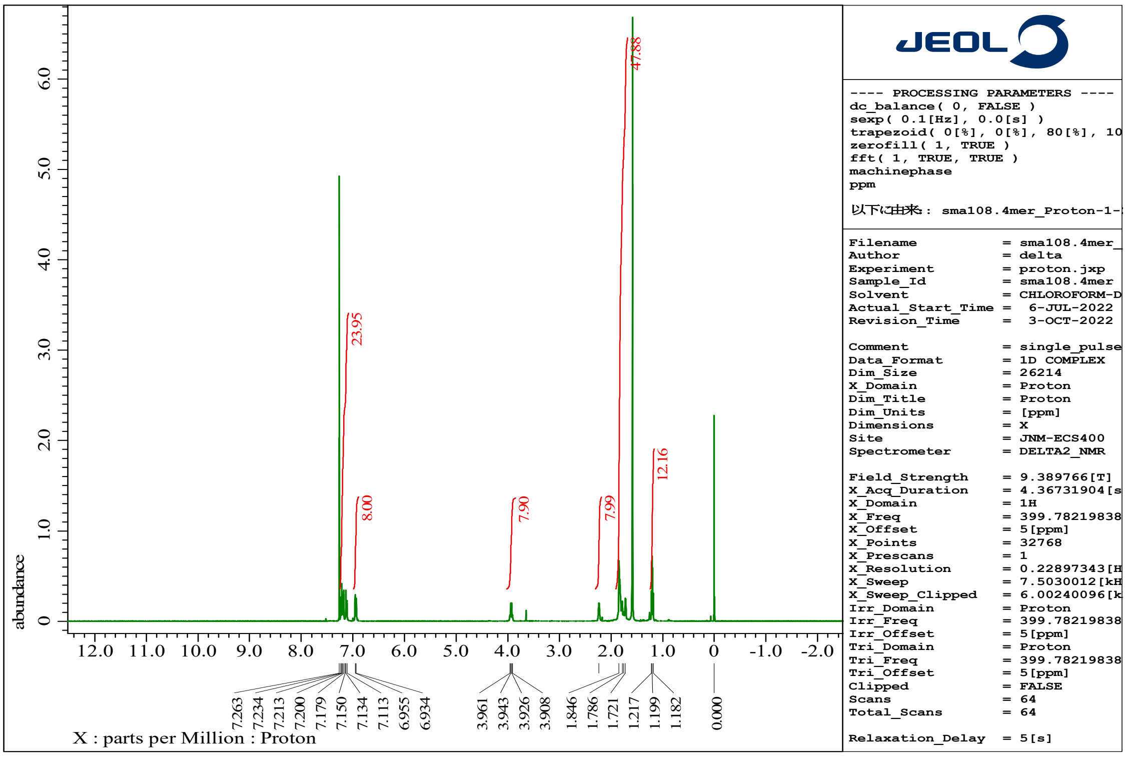

---- PROCESSING PARAMETERS ----
dc_balance(0, FALSE)
sexp(0.1[Hz], 0.0[s])
zerofill(1, TRUE)
fft(1, TRUE, TRUE)
machinephase
ppm
Filename = sma108.4mer_
Author = delta
Experiment = proton.jxp
Sample_Id = sma108.4mer
Solvent = CHLOROFORM-D
Actual_Start_Time = 6-JUL-2022
Revision_Time = 3-OCT-2022
Comment = single_pulse
Data_Format = 1D COMPLEX
Dim_Size = 26214
X_Domain = Proton
Dim_Title = Proton
Dim_Units = [ppm]
Dimensions = X
Site = JNM-ECS400
Spectrometer = DELTA2_NMR
Field_Strength = 9.389766[T]
X_Domain = 1H
X_Offset = 5[ppm]
X_Points = 32768
X_Prescans = 1
Irr_Domain = Proton
Irr_Offset = 5[ppm]
Tri_Domain = Proton
Tri_Offset = 5[ppm]
Clipped = FALSE
Scans = 64
Total_Scans = 64
Relaxation_Delay = 5[s]
23.95
8.00
7.90
7.99
47.88
12.16
7.263 7.234 7.213 7.200 7.179 7.150 7.134 7.113 6.955 6.934
3.961 3.943 3.926 3.908
1.846 1.786 1.721 1.217 1.199 1.182
0.000
abundance
X : parts per Million : Proton

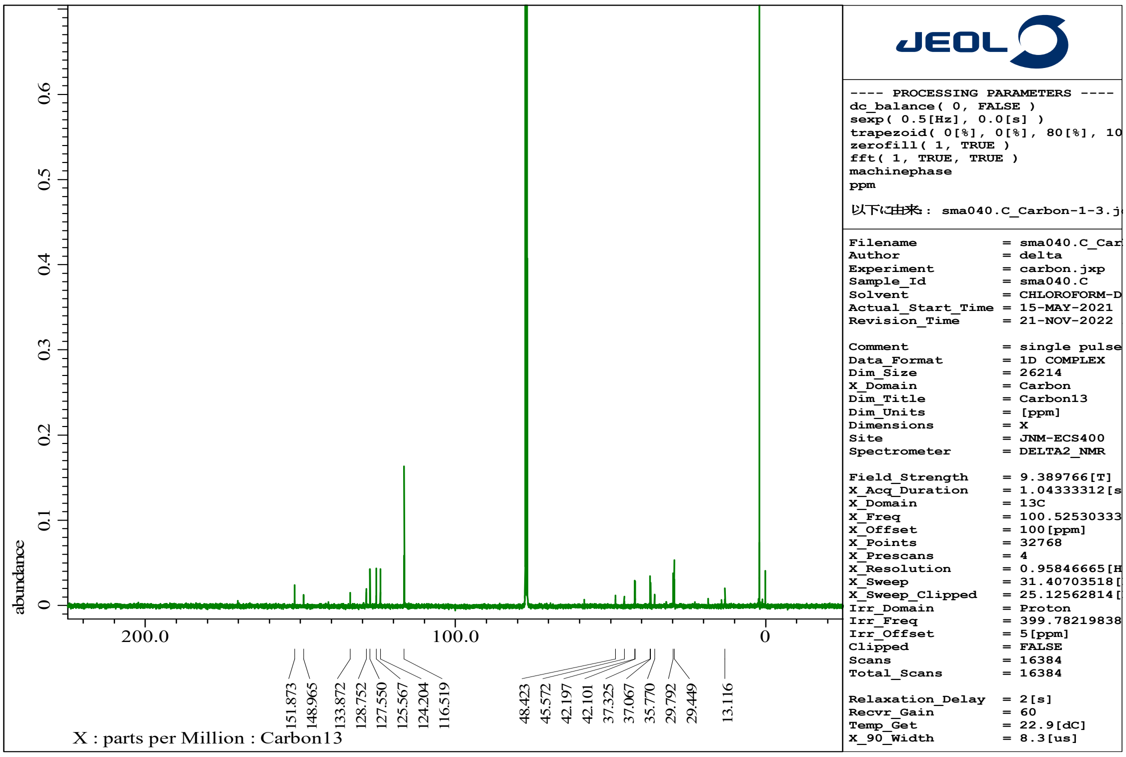

---- PROCESSING PARAMETERS ----
dc_balance(0, FALSE)
sexp(0.5[Hz], 0.0[s])
zerofill(1, TRUE)
fft(1, TRUE, TRUE)
machinephase
ppm
Author = delta
Experiment = carbon.jxp
Sample_Id = sma040.C
Solvent = CHLOROFORM-D
Actual_Start_Time = 15-MAY-2021
Revision_Time = 21-NOV-2022
Comment = single pulse
Data_Format = 1D COMPLEX
Dim_Size = 26214
X_Domain = Carbon
Dim_Title = Carbon13
Dim_Units = [ppm]
Dimensions = X
Site = JNM-ECS400
Spectrometer = DELTA2_NMR
Field_Strength = 9.389766[T]
X_Domain = 13C
X_Offset = 100[ppm]
X_Points = 32768
X_Prescans = 4
Irr_Domain = Proton
Irr_Offset = 5[ppm]
Clipped = FALSE
Scans = 16384
Total_Scans = 16384
Relaxation_Delay = 2[s]
Recvr_Gain = 60
Temp_Get = 22.9[dC]
X_90_Width = 8.3[us]
151.873 148.965 133.872 128.752 127.550 125.567 124.204 116.519
48.423 45.572 42.197 42.101 37.325 37.067 35.770 29.792 29.449 13.116
abundance
X : parts per Million : Carbon13

11d

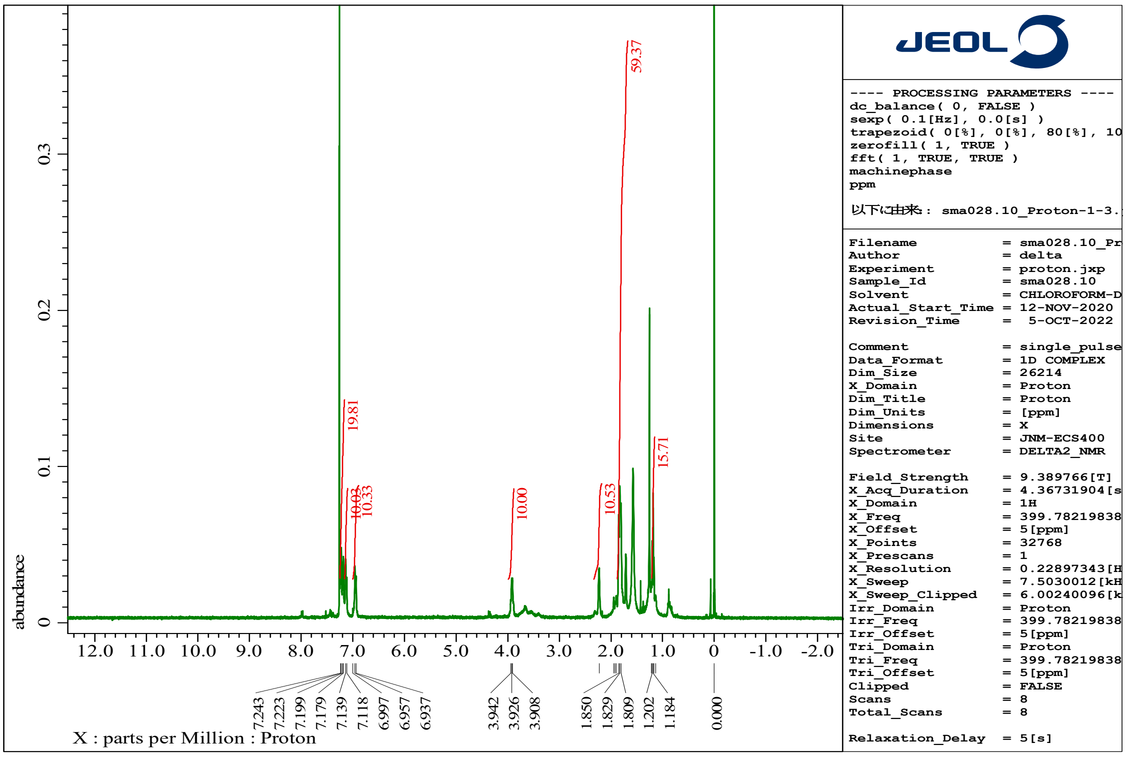

JEOL
---- PROCESSING PARAMETERS ----
dc_balance(0, FALSE)
sexp(0.1[Hz], 0.0[s])
trapezoid(0[%], 0[%], 80[%], 10
zerofill(1, TRUE)
fft(1, TRUE, TRUE)
machinephase
ppm
以下に由来： sma028.10_Proton-1-3.
Filename = sma028.10_Pr
Author = delta
Experiment = proton.jxp
Sample_Id = sma028.10
Solvent = CHLOROFORM-D
Actual_Start_Time = 12-NOV-2020
Revision_Time = 5-OCT-2022
Comment = single_pulse
Data_Format = 1D COMPLEX
Dim_Size = 26214
X_Domain = Proton
Dim_Title = Proton
Dim_Units = [ppm]
Dimensions = X
Site = JNM-ECS400
Spectrometer = DELTA2_NMR
Field_Strength = 9.389766[T]
X_Acq_Duration = 4.36731904[s
X_Domain = 1H
X_Freq = 399.78219838
X_Offset = 5[ppm]
X_Points = 32768
X_Prescans = 1
X_Resolution = 0.22897343[H
X_Sweep = 7.5030012[kH
X_Sweep_Clipped = 6.00240096[k
Irr_Domain = Proton
Irr_Freq = 399.78219838
Irr_Offset = 5[ppm]
Tri_Domain = Proton
Tri_Freq = 399.78219838
Tri_Offset = 5[ppm]
Clipped = FALSE
Scans = 8
Total_Scans = 8
Relaxation_Delay = 5[s]
abundance
0
0.1
0.2
0.3
19.81
10.03
10.33
10.00
10.53
59.37
15.71
12.0 11.0 10.0 9.0 8.0 7.0 6.0 5.0 4.0 3.0 2.0 1.0 0 -1.0 -2.0
7.243
7.223
7.199
7.179
7.139
7.118
6.997
6.957
6.937
3.942
3.926
3.908
1.850
1.829
1.809
1.202
1.184
0.000
X : parts per Million : Proton

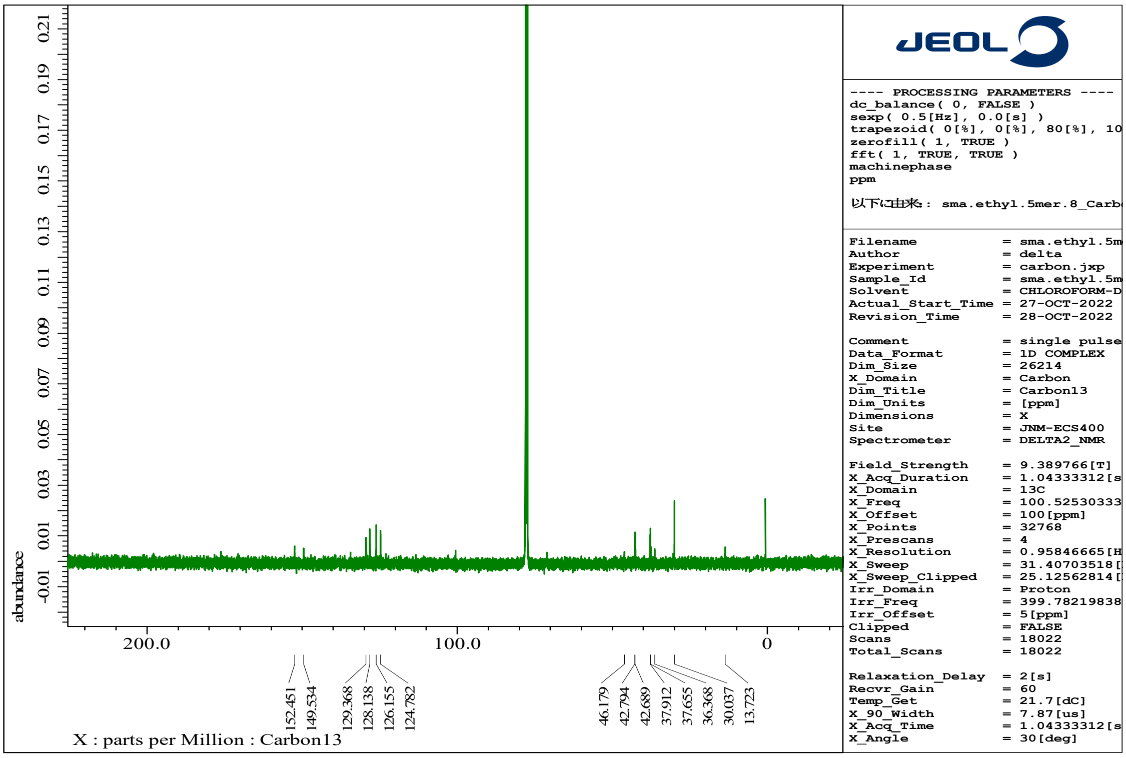

JEOL
---- PROCESSING PARAMETERS ----
dc_balance(0, FALSE)
sexp(0.5[Hz], 0.0[s])
trapezoid(0[%], 0[%], 80[%], 10
zerofill(1, TRUE)
fft(1, TRUE, TRUE)
machinephase
ppm
以下に由来： sma.ethyl.5mer.8_Carb
Filename = sma.ethyl.5m
Author = delta
Experiment = carbon.jxp
Sample_Id = sma.ethyl.5m
Solvent = CHLOROFORM-D
Actual_Start_Time = 27-OCT-2022
Revision_Time = 28-OCT-2022
Comment = single pulse
Data_Format = 1D COMPLEX
Dim_Size = 26214
X_Domain = Carbon
Dim_Title = Carbon13
Dim_Units = [ppm]
Dimensions = X
Site = JNM-ECS400
Spectrometer = DELTA2_NMR
Field_Strength = 9.389766[T]
X_Acq_Duration = 1.04333312[s
X_Domain = 13C
X_Freq = 100.52530333
X_Offset = 100[ppm]
X_Points = 32768
X_Prescans = 4
X_Resolution = 0.95846665[H
X_Sweep = 31.40703518[
X_Sweep_Clipped = 25.12562814[
Irr_Domain = Proton
Irr_Freq = 399.78219838
Irr_Offset = 5[ppm]
Clipped = FALSE
Scans = 18022
Total_Scans = 18022
Relaxation_Delay = 2[s]
Recvr_Gain = 60
Temp_Get = 21.7[dC]
X_90_Width = 7.87[us]
X_Acq_Time = 1.04333312[s
X_Angle = 30[deg]
abundance
-0.01
0.01
0.03
0.05
0.07
0.09
0.11
0.13
0.15
0.17
0.19
0.21
200.0
100.0
0
152.451
149.534
129.368
128.138
126.155
124.782
46.179
42.794
42.689
37.912
37.655
36.368
30.037
13.723
X : parts per Million : Carbon13

12

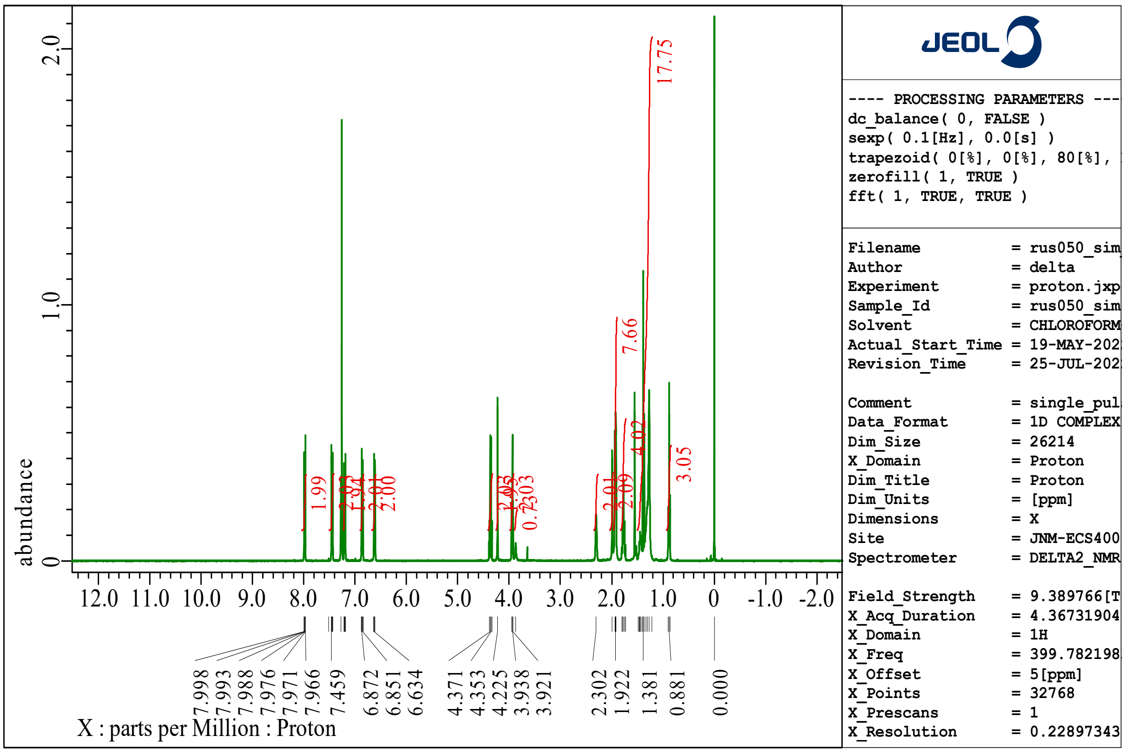
JEOL
---- PROCESSING PARAMETERS ---
dc_balance(0, FALSE)
sexp(0.1[Hz], 0.0[s])
trapezoid(0[%], 0[%], 80[%],
zerofill(1, TRUE)
fft(1, TRUE, TRUE)
Filename = rus050_sim
Author = delta
Experiment = proton.jxp
Sample_Id = rus050_sim
Solvent = CHLOROFORM
Actual_Start_Time = 19-MAY-202
Revision_Time = 25-JUL-202
Comment = single_pul
Data_Format = 1D COMPLEX
Dim_Size = 26214
X_Domain = Proton
Dim_Title = Proton
Dim_Units = [ppm]
Dimensions = X
Site = JNM-ECS400
Spectrometer = DELTA2_NMR
Field_Strength = 9.389766[T
X_Acq_Duration = 4.36731904
X_Domain = 1H
X_Freq = 399.782198
X_Offset = 5[ppm]
X_Points = 32768
X_Prescans = 1
X_Resolution = 0.22897343
abundance
X : parts per Million : Proton
7.998
7.993
7.988
7.976
7.971
7.966
7.459
6.872
6.851
6.634
4.371
4.353
4.225
3.938
3.921
2.302
1.922
1.381
0.881
0.000
1.99
2.01
2.00
17.75
7.66
3.05

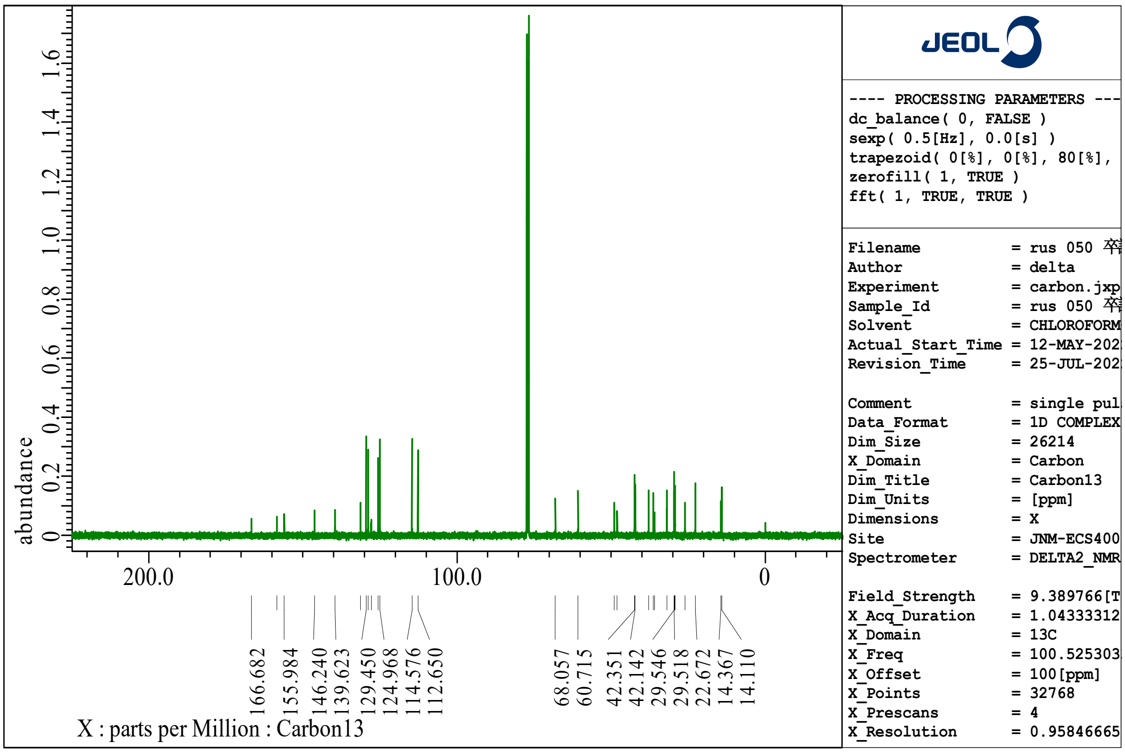
JEOL
---- PROCESSING PARAMETERS ---
dc_balance(0, FALSE)
sexp(0.5[Hz], 0.0[s])
trapezoid(0[%], 0[%], 80[%],
zerofill(1, TRUE)
fft(1, TRUE, TRUE)
Filename = rus 050
Author = delta
Experiment = carbon.jxp
Sample_Id = rus 050
Solvent = CHLOROFORM
Actual_Start_Time = 12-MAY-202
Revision_Time = 25-JUL-202
Comment = single pul
Data_Format = 1D COMPLEX
Dim_Size = 26214
X_Domain = Carbon
Dim_Title = Carbon13
Dim_Units = [ppm]
Dimensions = X
Site = JNM-ECS400
Spectrometer = DELTA2_NMR
Field_Strength = 9.389766[T
X_Acq_Duration = 1.04333312
X_Domain = 13C
X_Freq = 100.525303
X_Offset = 100[ppm]
X_Points = 32768
X_Prescans = 4
X_Resolution = 0.95846665
abundance
X : parts per Million : Carbon13
166.682
155.984
146.240
139.623
129.450
124.968
114.576
112.650
68.057
60.715
42.351
42.142
29.546
29.518
22.672
14.367
14.110

13a

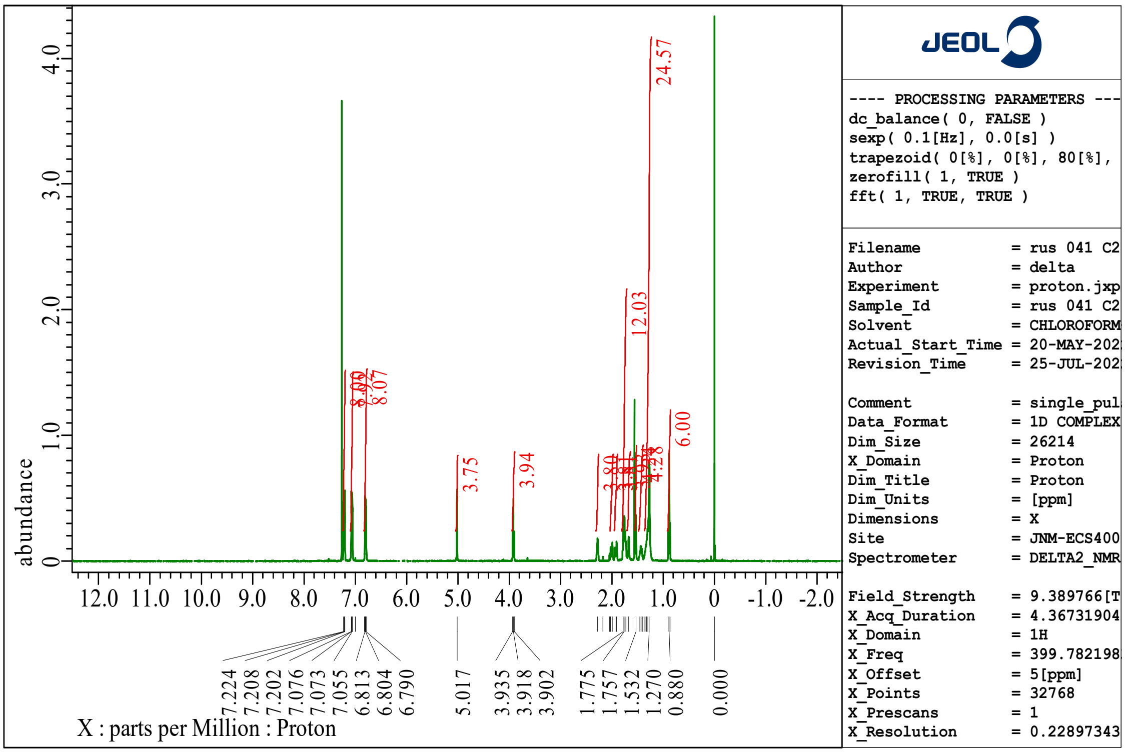
abundance
0
1.0
2.0
3.0
4.0
24.57
12.03
6.00
3.94
3.75
8.07
12.0 11.0 10.0 9.0 8.0 7.0 6.0 5.0 4.0 3.0 2.0 1.0 0 -1.0 -2.0
7.224
7.208
7.202
7.076
7.073
7.055
6.813
6.804
6.790
5.017
3.935
3.918
3.902
1.775
1.757
1.532
1.270
0.880
0.000
X : parts per Million : Proton
JEOL
---- PROCESSING PARAMETERS ---
dc_balance(0, FALSE)
sexp(0.1[Hz], 0.0[s])
trapezoid(0[%], 0[%], 80[%],
zerofill(1, TRUE)
fft(1, TRUE, TRUE)
Filename = rus 041 C2
Author = delta
Experiment = proton.jxp
Sample_Id = rus 041 C2
Solvent = CHLOROFORM
Actual_Start_Time = 20-MAY-202
Revision_Time = 25-JUL-202
Comment = single_pul
Data_Format = 1D COMPLEX
Dim_Size = 26214
X_Domain = Proton
Dim_Title = Proton
Dim_Units = [ppm]
Dimensions = X
Site = JNM-ECS400
Spectrometer = DELTA2_NMR
Field_Strength = 9.389766[T
X_Acq_Duration = 4.36731904
X_Domain = 1H
X_Freq = 399.782198
X_Offset = 5[ppm]
X_Points = 32768
X_Prescans = 1
X_Resolution = 0.22897343

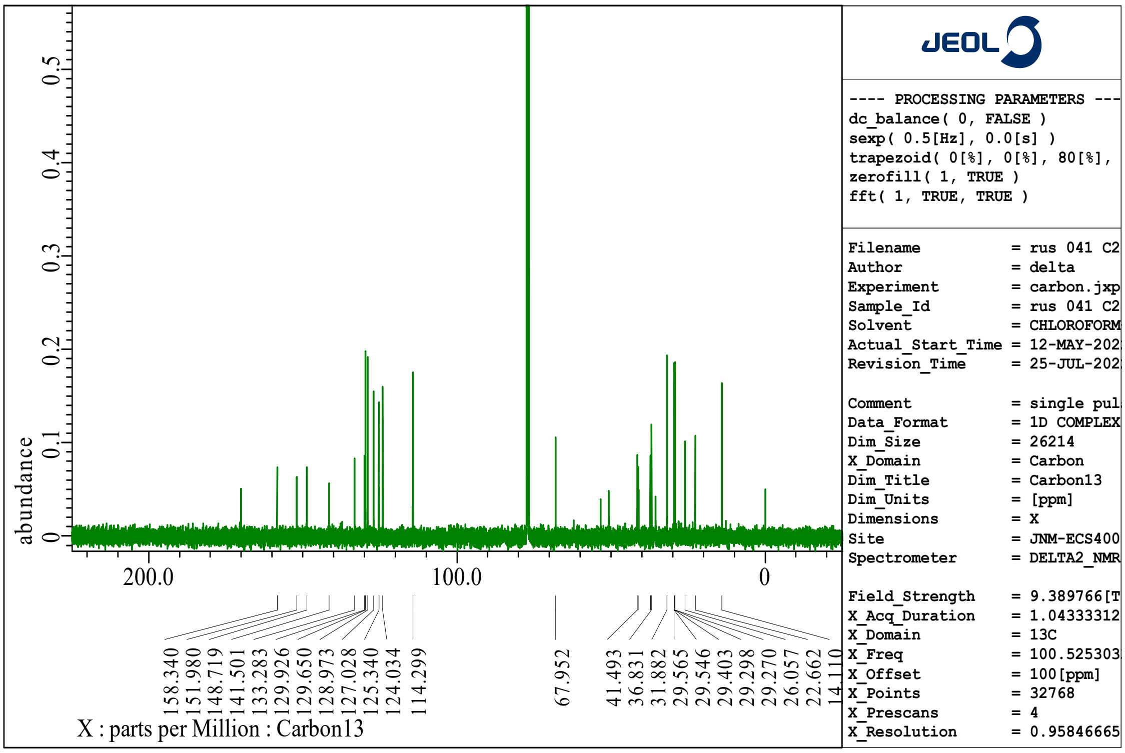
abundance
0
0.1
0.2
0.3
0.4
0.5
200.0
100.0
0
158.340
151.980
148.719
141.501
133.283
129.926
129.650
128.973
127.028
125.340
124.034
114.299
67.952
41.493
36.831
31.882
29.565
29.546
29.403
29.298
29.270
26.057
22.662
14.110
X : parts per Million : Carbon13
JEOL
---- PROCESSING PARAMETERS ---
dc_balance(0, FALSE)
sexp(0.5[Hz], 0.0[s])
trapezoid(0[%], 0[%], 80[%],
zerofill(1, TRUE)
fft(1, TRUE, TRUE)
Filename = rus 041 C2
Author = delta
Experiment = carbon.jxp
Sample_Id = rus 041 C2
Solvent = CHLOROFORM
Actual_Start_Time = 12-MAY-202
Revision_Time = 25-JUL-202
Comment = single pul
Data_Format = 1D COMPLEX
Dim_Size = 26214
X_Domain = Carbon
Dim_Title = Carbon13
Dim_Units = [ppm]
Dimensions = X
Site = JNM-ECS400
Spectrometer = DELTA2_NMR
Field_Strength = 9.389766[T
X_Acq_Duration = 1.04333312
X_Domain = 13C
X_Freq = 100.525303
X_Offset = 100[ppm]
X_Points = 32768
X_Prescans = 4
X_Resolution = 0.95846665

13b

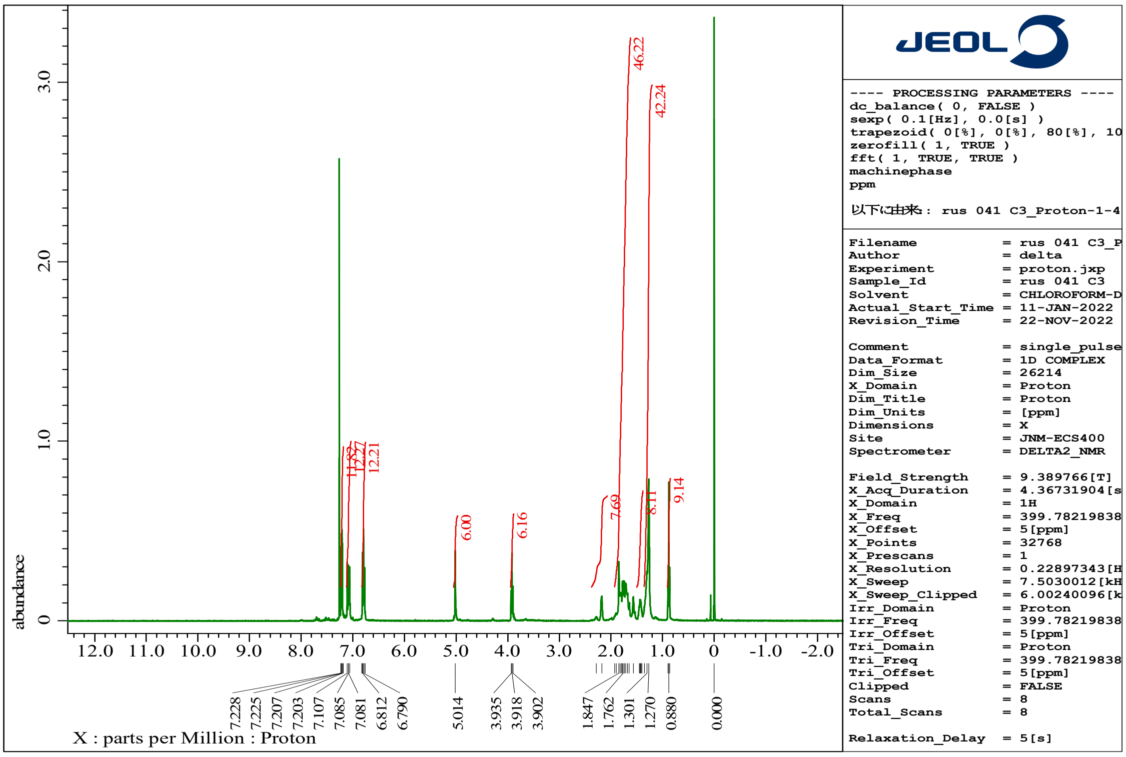

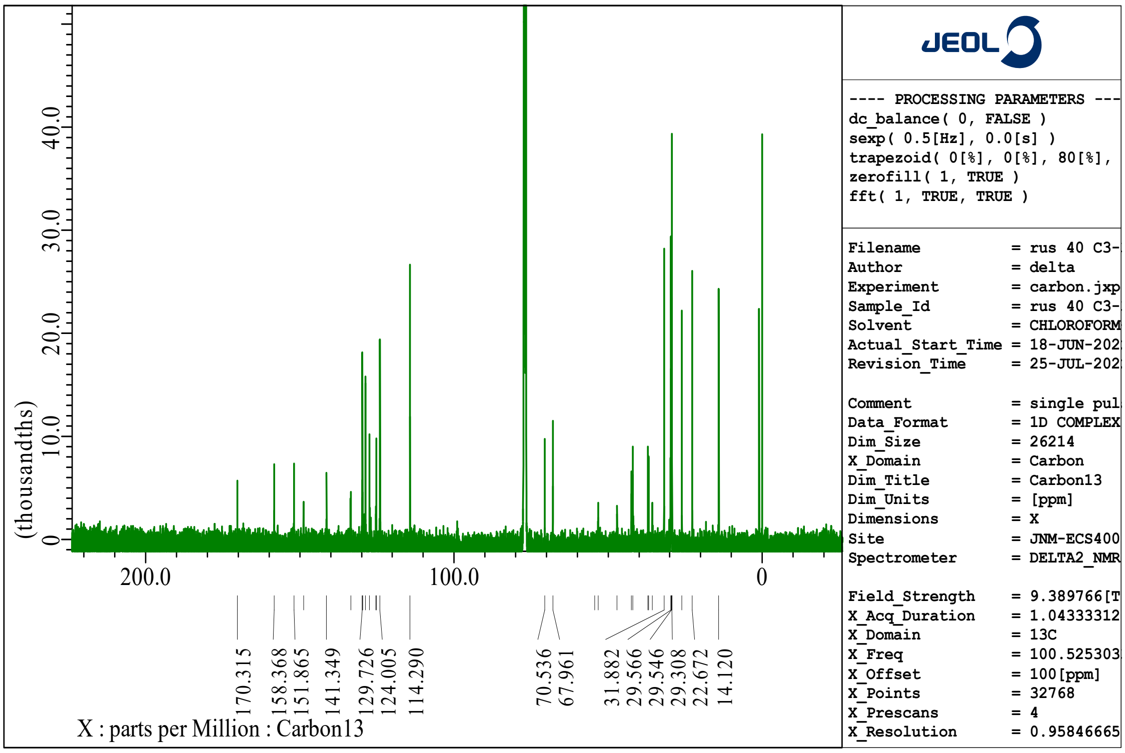

13c

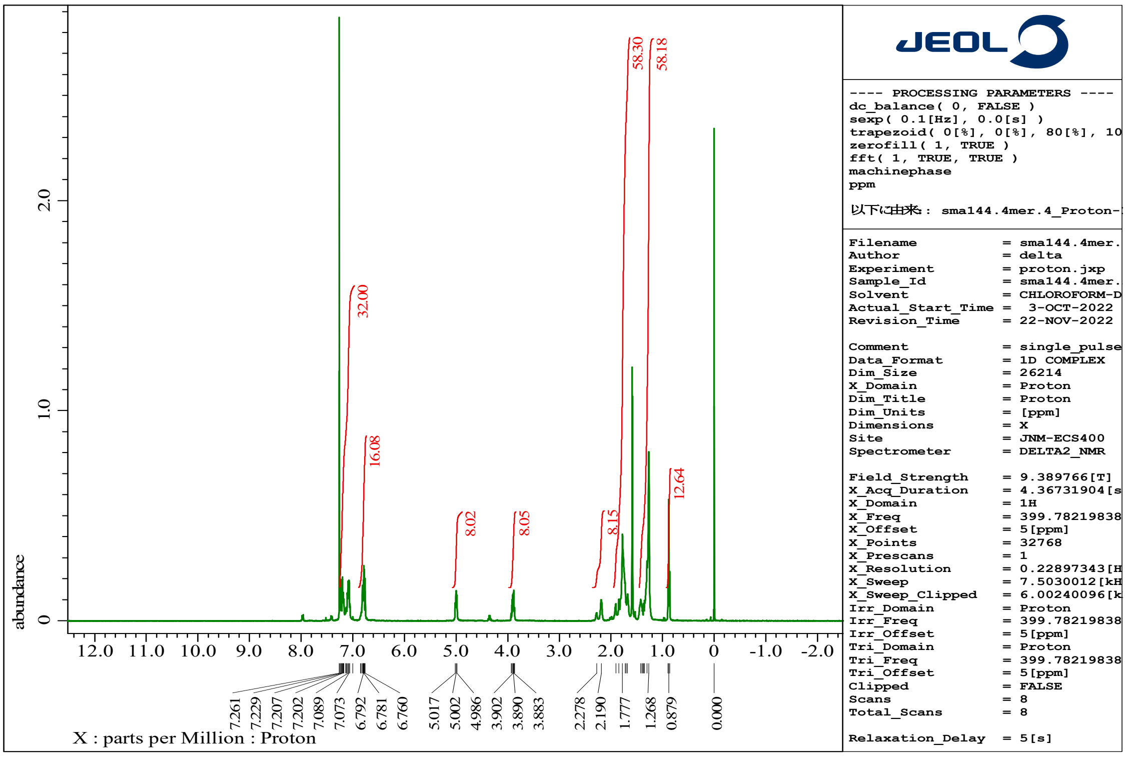
JEOL
---- PROCESSING PARAMETERS ----
dc_balance(0, FALSE)
sexp(0.1[Hz], 0.0[s])
trapezoid(0[%], 0[%], 80[%], 10
zerofill(1, TRUE)
fft(1, TRUE, TRUE)
machinephase
ppm
以下に由来: sma144.4mer.4_Proton-
Filename = sma144.4mer.
Author = delta
Experiment = proton.jxp
Sample_Id = sma144.4mer.
Solvent = CHLOROFORM-D
Actual_Start_Time = 3-OCT-2022
Revision_Time = 22-NOV-2022
Comment = single_pulse
Data_Format = 1D COMPLEX
Dim_Size = 26214
X_Domain = Proton
Dim_Title = Proton
Dim_Units = [ppm]
Dimensions = X
Site = JNM-ECS400
Spectrometer = DELTA2_NMR
Field_Strength = 9.389766[T]
X_Acq_Duration = 4.36731904[s
X_Domain = 1H
X_Freq = 399.78219838
X_Offset = 5[ppm]
X_Points = 32768
X_Prescans = 1
X_Resolution = 0.22897343[H
X_Sweep = 7.5030012[kH
X_Sweep_Clipped = 6.00240096[k
Irr_Domain = Proton
Irr_Freq = 399.78219838
Irr_Offset = 5[ppm]
Tri_Domain = Proton
Tri_Freq = 399.78219838
Tri_Offset = 5[ppm]
Clipped = FALSE
Scans = 8
Total_Scans = 8
Relaxation_Delay = 5[s]
abundance
0
1.0
2.0
32.00
16.08
8.02
8.05
8.15
58.30
58.18
12.64
12.0 11.0 10.0 9.0 8.0 7.0 6.0 5.0 4.0 3.0 2.0 1.0 0 -1.0 -2.0
7.261
7.229
7.207
7.202
7.089
7.073
6.792
6.781
6.760
5.017
5.002
4.986
3.902
3.890
3.883
2.278
2.190
1.777
1.268
0.879
0.000
X : parts per Million : Proton

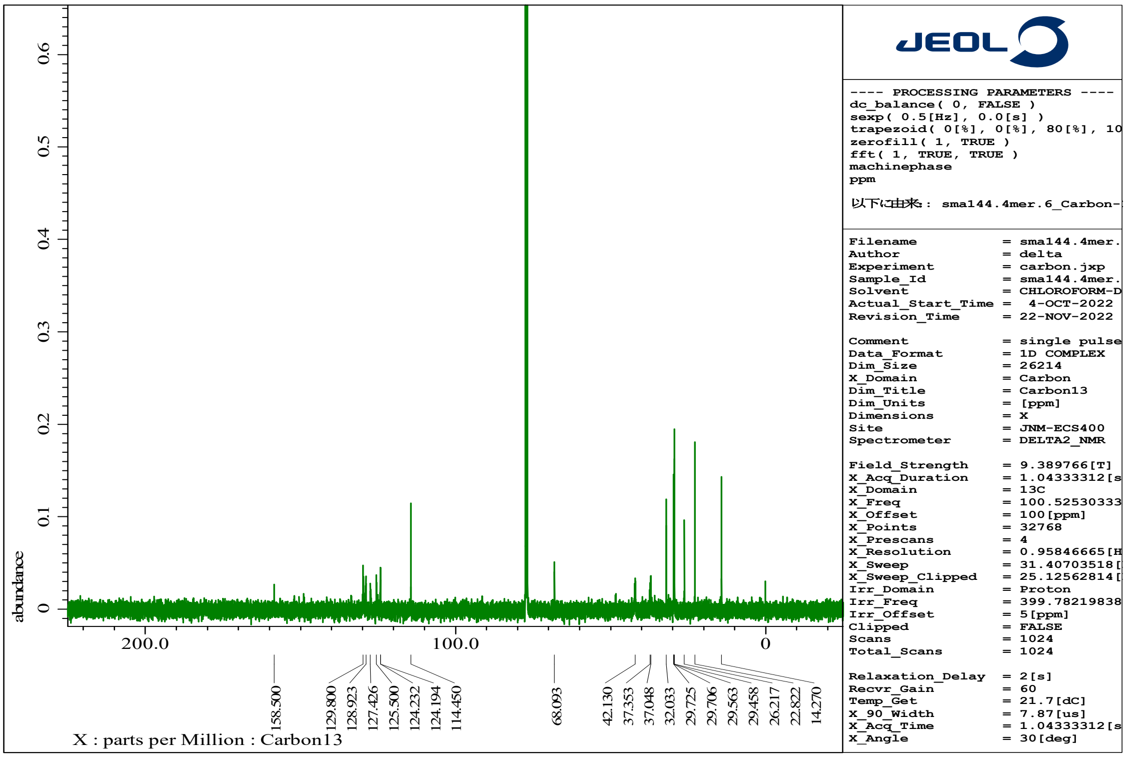
JEOL
---- PROCESSING PARAMETERS ----
dc_balance(0, FALSE)
sexp(0.5[Hz], 0.0[s])
trapezoid(0[%], 0[%], 80[%], 10
zerofill(1, TRUE)
fft(1, TRUE, TRUE)
machinephase
ppm
以下に由来: sma144.4mer.6_Carbon-
Filename = sma144.4mer.
Author = delta
Experiment = carbon.jxp
Sample_Id = sma144.4mer.
Solvent = CHLOROFORM-D
Actual_Start_Time = 4-OCT-2022
Revision_Time = 22-NOV-2022
Comment = single_pulse
Data_Format = 1D COMPLEX
Dim_Size = 26214
X_Domain = Carbon
Dim_Title = Carbon13
Dim_Units = [ppm]
Dimensions = X
Site = JNM-ECS400
Spectrometer = DELTA2_NMR
Field_Strength = 9.389766[T]
X_Acq_Duration = 1.04333312[s
X_Domain = 13C
X_Freq = 100.52530333
X_Offset = 100[ppm]
X_Points = 32768
X_Prescans = 4
X_Resolution = 0.95846665[H
X_Sweep = 31.40703518[
X_Sweep_Clipped = 25.12562814[
Irr_Domain = Proton
Irr_Freq = 399.78219838
Irr_Offset = 5[ppm]
Clipped = FALSE
Scans = 1024
Total_Scans = 1024
Relaxation_Delay = 2[s]
Recvr_Gain = 60
Temp_Get = 21.7[dC]
X_90_Width = 7.87[us]
X_Acq_Time = 1.04333312[s
X_Angle = 30[deg]
abundance
0
0.1
0.2
0.3
0.4
0.5
0.6
200.0
100.0
0
158.500
129.800
128.923
127.426
125.500
124.232
124.194
114.450
68.093
42.130
37.353
37.048
32.033
29.725
29.706
29.563
29.458
26.217
22.822
14.270
X : parts per Million : Carbon13

13d

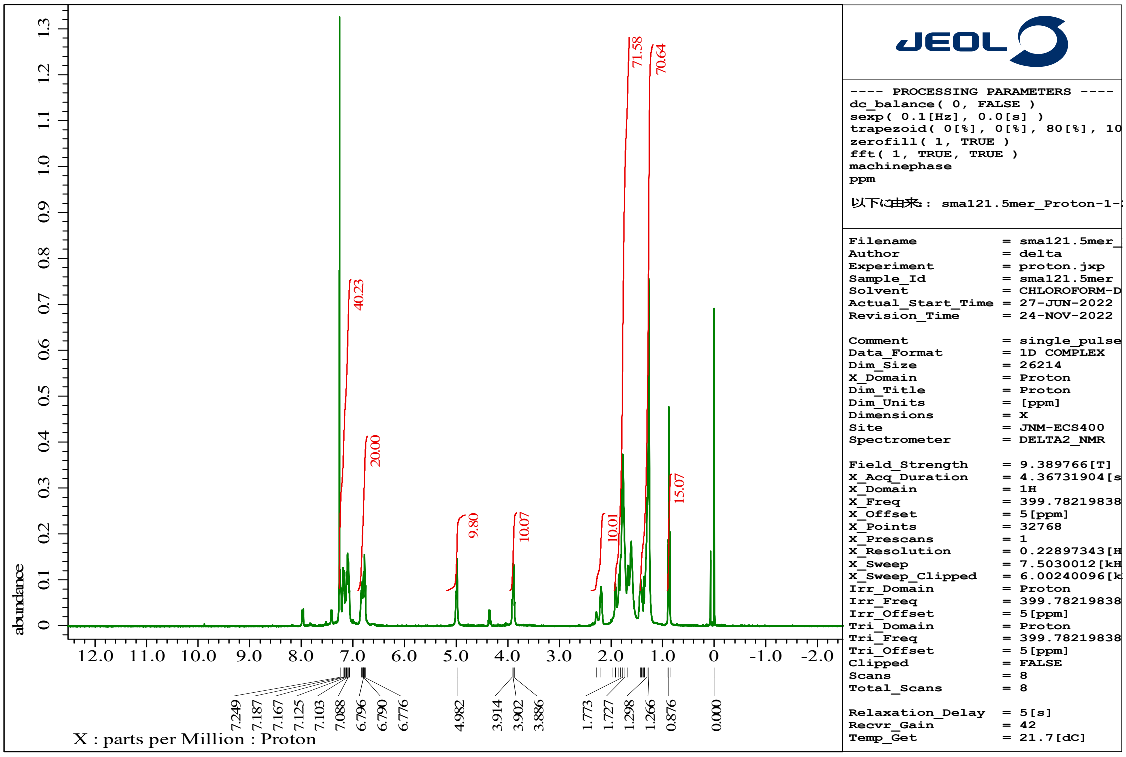
JEOL
---- PROCESSING PARAMETERS ----
dc_balance(0, FALSE)
sexp(0.1[Hz], 0.0[s])
trapezoid(0[%], 0[%], 80[%], 10
zerofill(1, TRUE)
fft(1, TRUE, TRUE)
machinephase
ppm
以下に由来： sma121.5mer_Proton-1-
Filename = sma121.5mer_
Author = delta
Experiment = proton.jxp
Sample_Id = sma121.5mer
Solvent = CHLOROFORM-D
Actual_Start_Time = 27-JUN-2022
Revision_Time = 24-NOV-2022
Comment = single_pulse
Data_Format = 1D COMPLEX
Dim_Size = 26214
X_Domain = Proton
Dim_Title = Proton
Dim_Units = [ppm]
Dimensions = X
Site = JNM-ECS400
Spectrometer = DELTA2_NMR
Field_Strength = 9.389766[T]
X_Acq_Duration = 4.36731904[s
X_Domain = 1H
X_Freq = 399.78219838
X_Offset = 5[ppm]
X_Points = 32768
X_Prescans = 1
X_Resolution = 0.22897343[H
X_Sweep = 7.5030012[kH
X_Sweep_Clipped = 6.00240096[k
Irr_Domain = Proton
Irr_Freq = 399.78219838
Irr_Offset = 5[ppm]
Tri_Domain = Proton
Tri_Freq = 399.78219838
Tri_Offset = 5[ppm]
Clipped = FALSE
Scans = 8
Total_Scans = 8
Relaxation_Delay = 5[s]
Recvr_Gain = 42
Temp_Get = 21.7[dC]
abundance
40.23
20.00
9.80
10.07
10.01
71.58
70.64
15.07
7.249 7.187 7.167 7.125 7.103 7.088 6.796 6.790 6.776 4.982 3.914 3.902 3.886 1.773 1.727 1.298 1.266 0.876 0.000
X : parts per Million : Proton

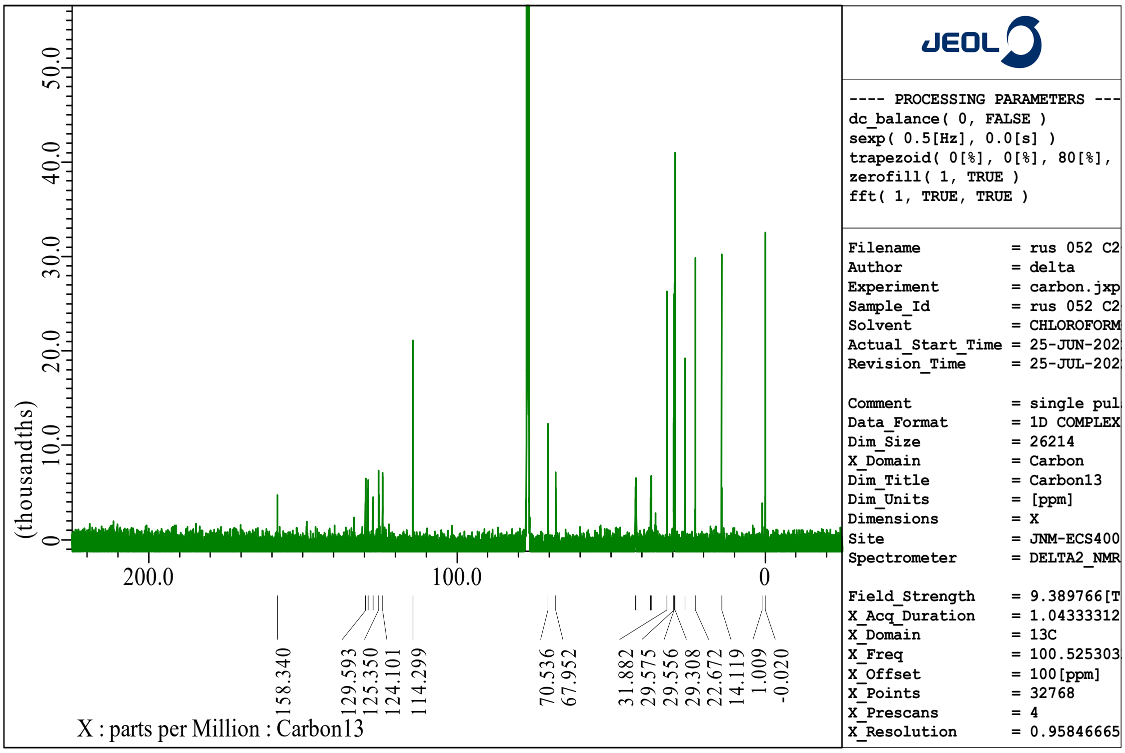
JEOL
---- PROCESSING PARAMETERS ---
dc_balance(0, FALSE)
sexp(0.5[Hz], 0.0[s])
trapezoid(0[%], 0[%], 80[%],
zerofill(1, TRUE)
fft(1, TRUE, TRUE)
Filename = rus 052 C2
Author = delta
Experiment = carbon.jxp
Sample_Id = rus 052 C2
Solvent = CHLOROFORM
Actual_Start_Time = 25-JUN-202
Revision_Time = 25-JUL-202
Comment = single pul
Data_Format = 1D COMPLEX
Dim_Size = 26214
X_Domain = Carbon
Dim_Title = Carbon13
Dim_Units = [ppm]
Dimensions = X
Site = JNM-ECS400
Spectrometer = DELTA2_NMR
Field_Strength = 9.389766[T
X_Acq_Duration = 1.04333312
X_Domain = 13C
X_Freq = 100.525303
X_Offset = 100[ppm]
X_Points = 32768
X_Prescans = 4
X_Resolution = 0.95846665
(thousandths)
158.340 129.593 125.350 124.101 114.299 70.536 67.952 31.882 29.575 29.556 29.308 22.672 14.119 1.009 -0.020
X : parts per Million : Carbon13

14a

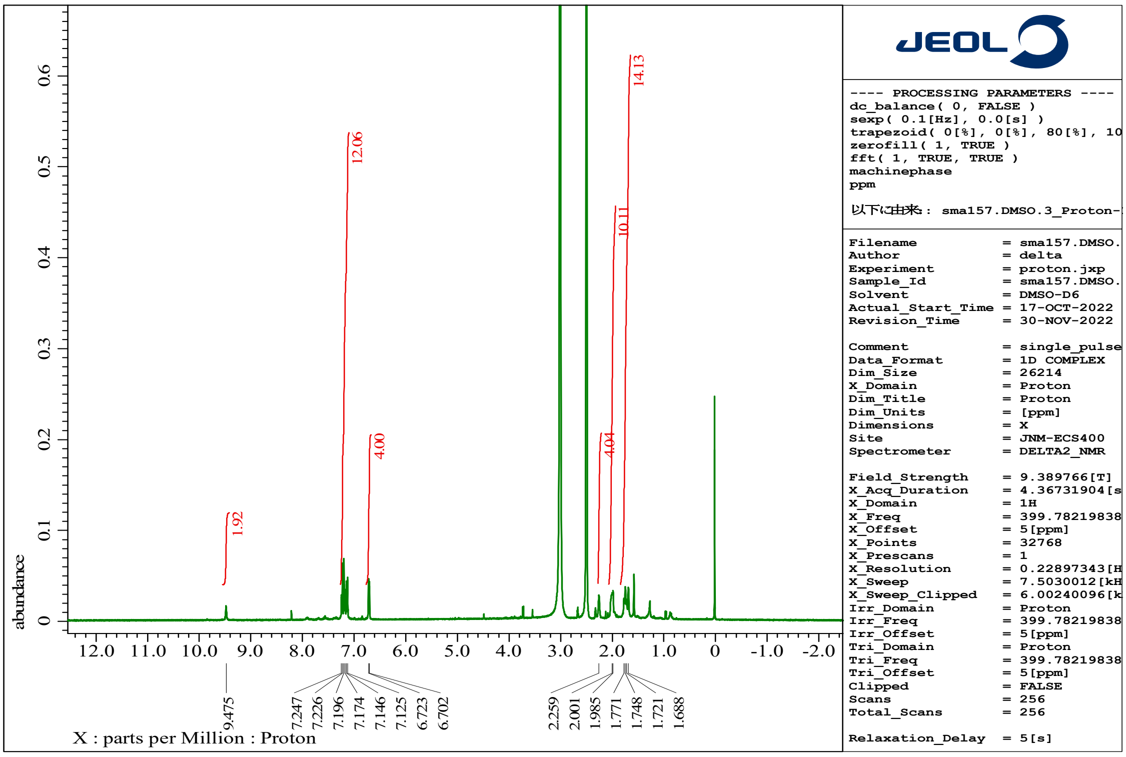
JEOL
---- PROCESSING PARAMETERS ----
dc_balance(0, FALSE)
sexp(0.1[Hz], 0.0[s])
trapezoid(0[%], 0[%], 80[%], 10
zerofill(1, TRUE)
fft(1, TRUE, TRUE)
machinephase
ppm
以下に由来： sma157.DMSO.3_Proton-
Filename = sma157.DMSO.
Author = delta
Experiment = proton.jxp
Sample_Id = sma157.DMSO.
Solvent = DMSO-D6
Actual_Start_Time = 17-OCT-2022
Revision_Time = 30-NOV-2022
Comment = single_pulse
Data_Format = 1D COMPLEX
Dim_Size = 26214
X_Domain = Proton
Dim_Title = Proton
Dim_Units = [ppm]
Dimensions = X
Site = JNM-ECS400
Spectrometer = DELTA2_NMR
Field_Strength = 9.389766[T]
X_Acq_Duration = 4.36731904[s
X_Domain = 1H
X_Freq = 399.78219838
X_Offset = 5[ppm]
X_Points = 32768
X_Prescans = 1
X_Resolution = 0.22897343[H
X_Sweep = 7.5030012[kH
X_Sweep_Clipped = 6.00240096[k
Irr_Domain = Proton
Irr_Freq = 399.78219838
Irr_Offset = 5[ppm]
Tri_Domain = Proton
Tri_Freq = 399.78219838
Tri_Offset = 5[ppm]
Clipped = FALSE
Scans = 256
Total_Scans = 256
Relaxation_Delay = 5[s]
abundance
0 0.1 0.2 0.3 0.4 0.5 0.6
1.92 12.06 4.00 4.04 10.11 14.13
12.0 11.0 10.0 9.0 8.0 7.0 6.0 5.0 4.0 3.0 2.0 1.0 0 -1.0 -2.0
9.475 7.247 7.226 7.196 7.174 7.146 7.125 6.723 6.702 2.259 2.001 1.985 1.771 1.748 1.721 1.688
X : parts per Million : Proton

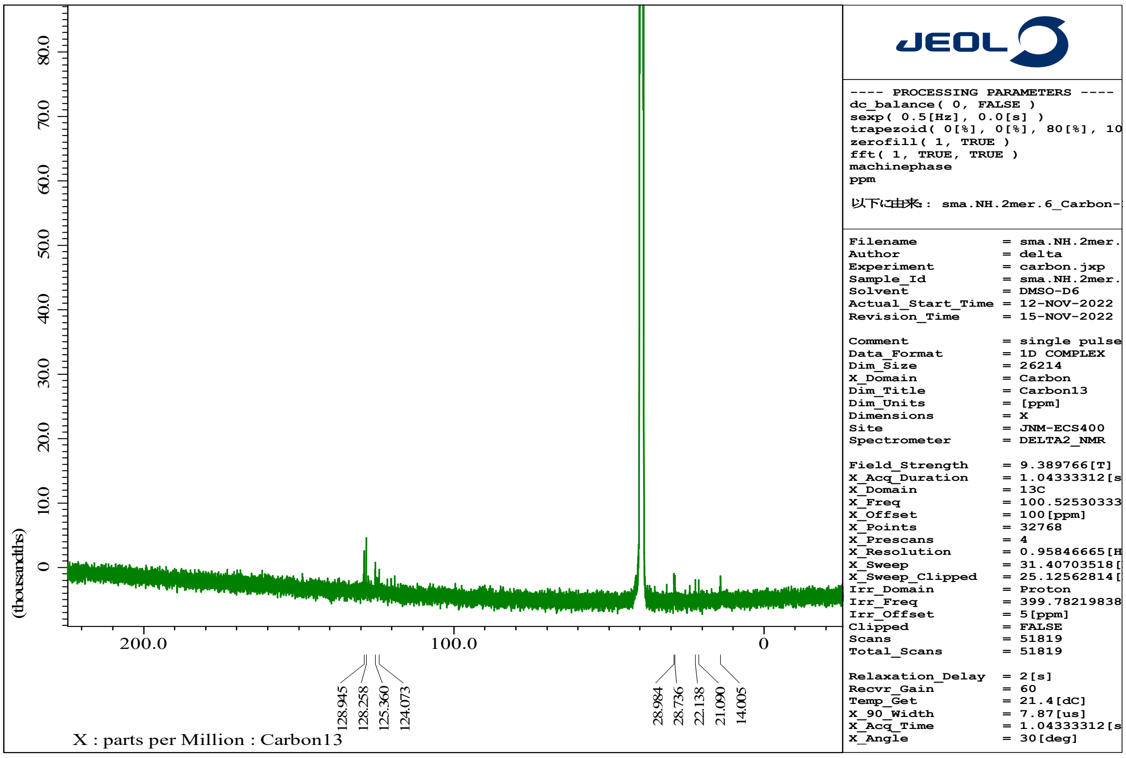
JEOL
---- PROCESSING PARAMETERS ----
dc_balance(0, FALSE)
sexp(0.5[Hz], 0.0[s])
trapezoid(0[%], 0[%], 80[%], 10
zerofill(1, TRUE)
fft(1, TRUE, TRUE)
machinephase
ppm
以下に由来： sma.NH.2mer.6_Carbon-
Filename = sma.NH.2mer.
Author = delta
Experiment = carbon.jxp
Sample_Id = sma.NH.2mer.
Solvent = DMSO-D6
Actual_Start_Time = 12-NOV-2022
Revision_Time = 15-NOV-2022
Comment = single pulse
Data_Format = 1D COMPLEX
Dim_Size = 26214
X_Domain = Carbon
Dim_Title = Carbon13
Dim_Units = [ppm]
Dimensions = X
Site = JNM-ECS400
Spectrometer = DELTA2_NMR
Field_Strength = 9.389766[T]
X_Acq_Duration = 1.04333312[s
X_Domain = 13C
X_Freq = 100.52530333
X_Offset = 100[ppm]
X_Points = 32768
X_Prescans = 4
X_Resolution = 0.95846665[H
X_Sweep = 31.40703518[
X_Sweep_Clipped = 25.12562814[
Irr_Domain = Proton
Irr_Freq = 399.78219838
Irr_Offset = 5[ppm]
Clipped = FALSE
Scans = 51819
Total_Scans = 51819
Relaxation_Delay = 2[s]
Recvr_Gain = 60
Temp_Get = 21.4[dC]
X_90_Width = 7.87[us]
X_Acq_Time = 1.04333312[s
X_Angle = 30[deg]
(thousandths)
0 10.0 20.0 30.0 40.0 50.0 60.0 70.0 80.0
200.0 100.0 0
128.945 128.258 125.360 124.073 28.984 28.736 22.138 21.090 14.005
X : parts per Million : Carbon13

14b

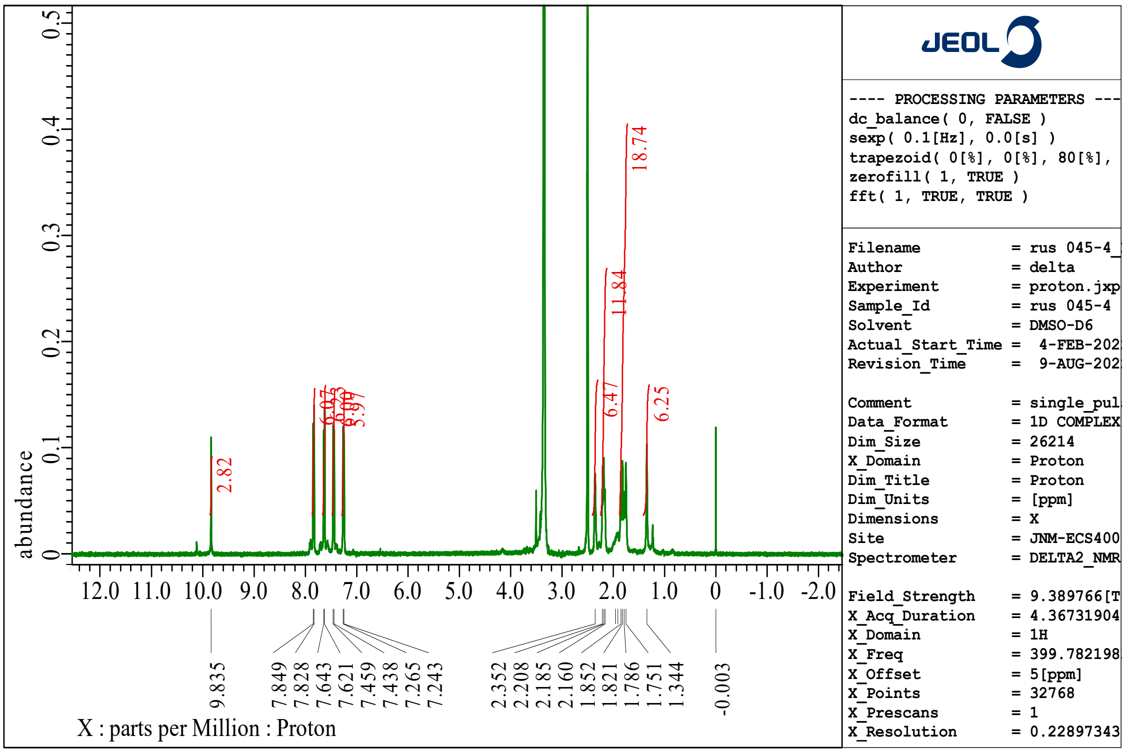

abundance
0
0.1
0.2
0.3
0.4
0.5
2.82
6.07
6.15
6.03
5.97
6.47
11.84
18.74
6.25
12.0 11.0 10.0 9.0 8.0 7.0 6.0 5.0 4.0 3.0 2.0 1.0 0 -1.0 -2.0
9.835
7.849 7.828 7.643 7.621 7.459 7.438 7.265 7.243
2.352 2.208 2.185 2.160 1.852 1.821 1.786 1.751 1.344
-0.003
X : parts per Million : Proton
---- PROCESSING PARAMETERS ---
dc_balance(0, FALSE)
sexp(0.1[Hz], 0.0[s])
trapezoid(0[%], 0[%], 80[%],
zerofill(1, TRUE)
fft(1, TRUE, TRUE)
Filename = rus 045-4_
Author = delta
Experiment = proton.jxp
Sample_Id = rus 045-4
Solvent = DMSO-D6
Actual_Start_Time = 4-FEB-202
Revision_Time = 9-AUG-202
Comment = single_pul
Data_Format = 1D COMPLEX
Dim_Size = 26214
X_Domain = Proton
Dim_Title = Proton
Dim_Units = [ppm]
Dimensions = X
Site = JNM-ECS400
Spectrometer = DELTA2_NMR
Field_Strength = 9.389766[T
X_Acq_Duration = 4.36731904
X_Domain = 1H
X_Freq = 399.782198
X_Offset = 5[ppm]
X_Points = 32768
X_Prescans = 1
X_Resolution = 0.22897343

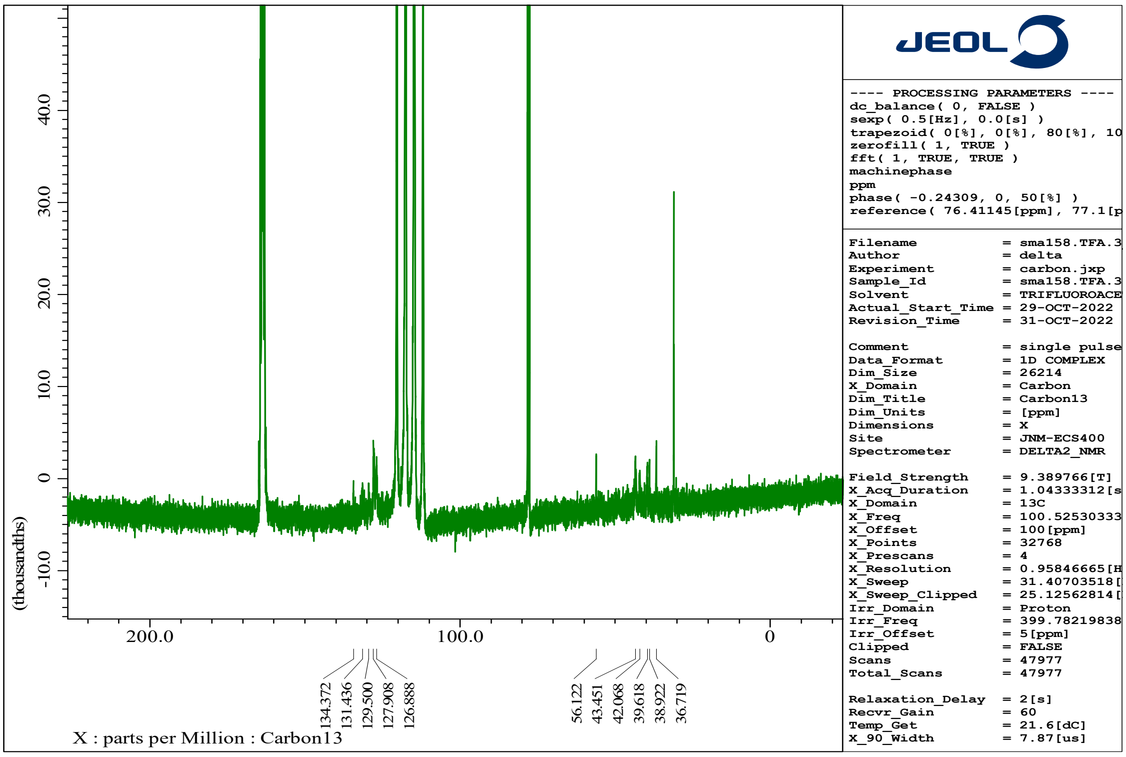

(thousandths)
-10.0
0
10.0
20.0
30.0
40.0
200.0 100.0 0
134.372 131.436 129.500 127.908 126.888
56.122 43.451 42.068 39.618 38.922 36.719
X : parts per Million : Carbon13
---- PROCESSING PARAMETERS ----
dc_balance(0, FALSE)
sexp(0.5[Hz], 0.0[s])
trapezoid(0[%], 0[%], 80[%], 10
zerofill(1, TRUE)
fft(1, TRUE, TRUE)
machinephase
ppm
phase(-0.24309, 0, 50[%])
reference(76.41145[ppm], 77.1[p
Filename = sma158.TFA.3
Author = delta
Experiment = carbon.jxp
Sample_Id = sma158.TFA.3
Solvent = TRIFLUOROACE
Actual_Start_Time = 29-OCT-2022
Revision_Time = 31-OCT-2022
Comment = single pulse
Data_Format = 1D COMPLEX
Dim_Size = 26214
X_Domain = Carbon
Dim_Title = Carbon13
Dim_Units = [ppm]
Dimensions = X
Site = JNM-ECS400
Spectrometer = DELTA2_NMR
Field_Strength = 9.389766[T]
X_Acq_Duration = 1.04333312[s
X_Domain = 13C
X_Freq = 100.52530333
X_Offset = 100[ppm]
X_Points = 32768
X_Prescans = 4
X_Resolution = 0.95846665[H
X_Sweep = 31.40703518[
X_Sweep_Clipped = 25.12562814[
Irr_Domain = Proton
Irr_Freq = 399.78219838
Irr_Offset = 5[ppm]
Clipped = FALSE
Scans = 47977
Total_Scans = 47977
Relaxation_Delay = 2[s]
Recvr_Gain = 60
Temp_Get = 21.6[dC]
X_90_Width = 7.87[us]

14c

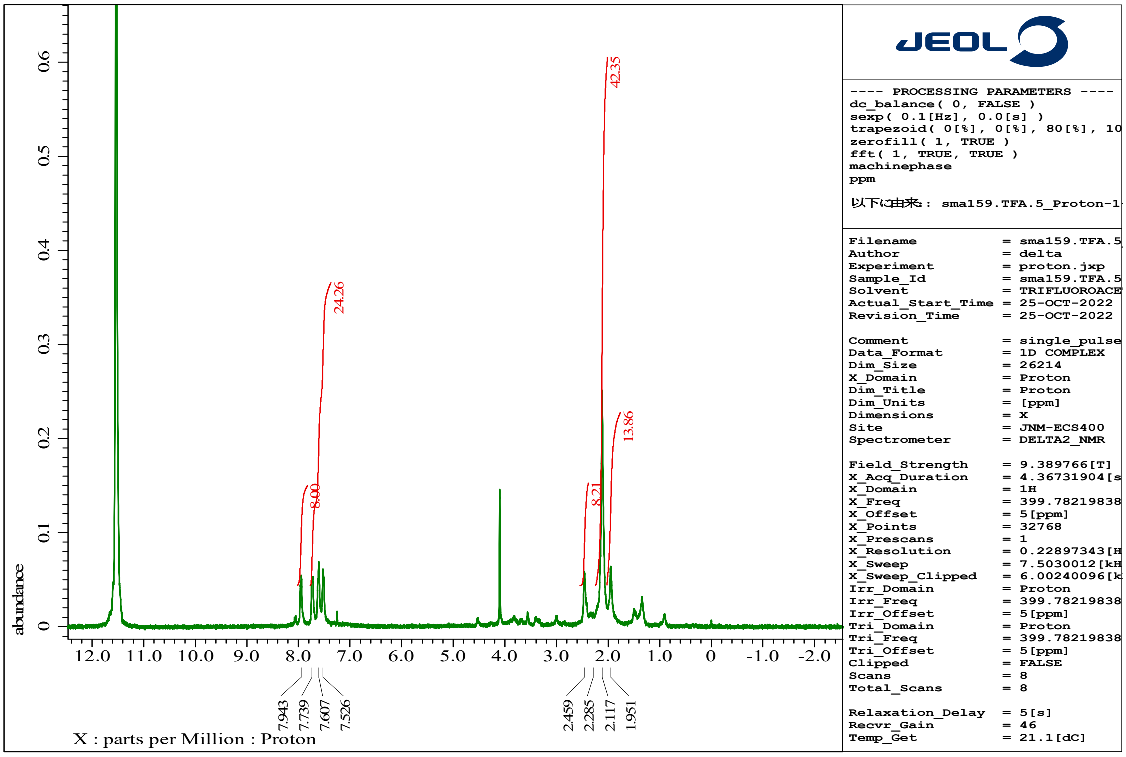

abundance
X : parts per Million : Proton
8.00
24.26
8.21
42.35
13.86
7.943
7.739
7.607
7.526
2.459
2.285
2.117
1.951
---- PROCESSING PARAMETERS ----
dc_balance(0, FALSE)
sexp(0.1[Hz], 0.0[s])
trapezoid(0[%], 0[%], 80[%], 10
zerofill(1, TRUE)
fft(1, TRUE, TRUE)
machinephase
ppm
以下に由来： sma159.TFA.5_Proton-1
Filename = sma159.TFA.5
Author = delta
Experiment = proton.jxp
Sample_Id = sma159.TFA.5
Solvent = TRIFLUOROACE
Actual_Start_Time = 25-OCT-2022
Revision_Time = 25-OCT-2022
Comment = single_pulse
Data_Format = 1D COMPLEX
Dim_Size = 26214
X_Domain = Proton
Dim_Title = Proton
Dim_Units = [ppm]
Dimensions = X
Site = JNM-ECS400
Spectrometer = DELTA2_NMR
Field_Strength = 9.389766[T]
X_Acq_Duration = 4.36731904[s
X_Domain = 1H
X_Freq = 399.78219838
X_Offset = 5[ppm]
X_Points = 32768
X_Prescans = 1
X_Resolution = 0.22897343[H
X_Sweep = 7.5030012[kH
X_Sweep_Clipped = 6.00240096[k
Irr_Domain = Proton
Irr_Freq = 399.78219838
Irr_Offset = 5[ppm]
Tri_Domain = Proton
Tri_Freq = 399.78219838
Tri_Offset = 5[ppm]
Clipped = FALSE
Scans = 8
Total_Scans = 8
Relaxation_Delay = 5[s]
Recvr_Gain = 46
Temp_Get = 21.1[dC]

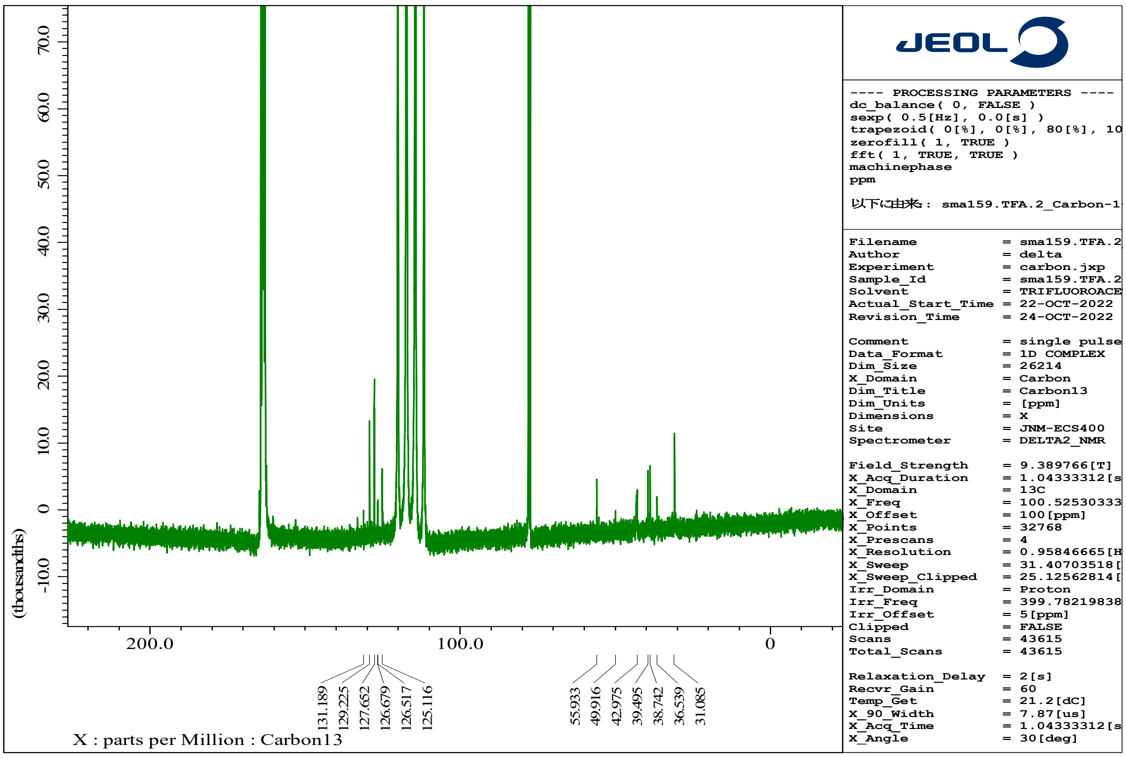

(thousandths)
X : parts per Million : Carbon13
131.189
129.225
127.652
126.679
126.517
125.116
55.933
49.916
42.975
39.495
38.742
36.539
31.085
---- PROCESSING PARAMETERS ----
dc_balance(0, FALSE)
sexp(0.5[Hz], 0.0[s])
trapezoid(0[%], 0[%], 80[%], 10
zerofill(1, TRUE)
fft(1, TRUE, TRUE)
machinephase
ppm
以下に由来： sma159.TFA.2_Carbon-1
Filename = sma159.TFA.2
Author = delta
Experiment = carbon.jxp
Sample_Id = sma159.TFA.2
Solvent = TRIFLUOROACE
Actual_Start_Time = 22-OCT-2022
Revision_Time = 24-OCT-2022
Comment = single pulse
Data_Format = 1D COMPLEX
Dim_Size = 26214
X_Domain = Carbon
Dim_Title = Carbon13
Dim_Units = [ppm]
Dimensions = X
Site = JNM-ECS400
Spectrometer = DELTA2_NMR
Field_Strength = 9.389766[T]
X_Acq_Duration = 1.04333312[s
X_Domain = 13C
X_Freq = 100.52530333
X_Offset = 100[ppm]
X_Points = 32768
X_Prescans = 4
X_Resolution = 0.95846665[H
X_Sweep = 31.40703518[
X_Sweep_Clipped = 25.12562814[
Irr_Domain = Proton
Irr_Freq = 399.78219838
Irr_Offset = 5[ppm]
Clipped = FALSE
Scans = 43615
Total_Scans = 43615
Relaxation_Delay = 2[s]
Recvr_Gain = 60
Temp_Get = 21.2[dC]
X_90_Width = 7.87[us]
X_Acq_Time = 1.04333312[s
X_Angle = 30[deg]

14d

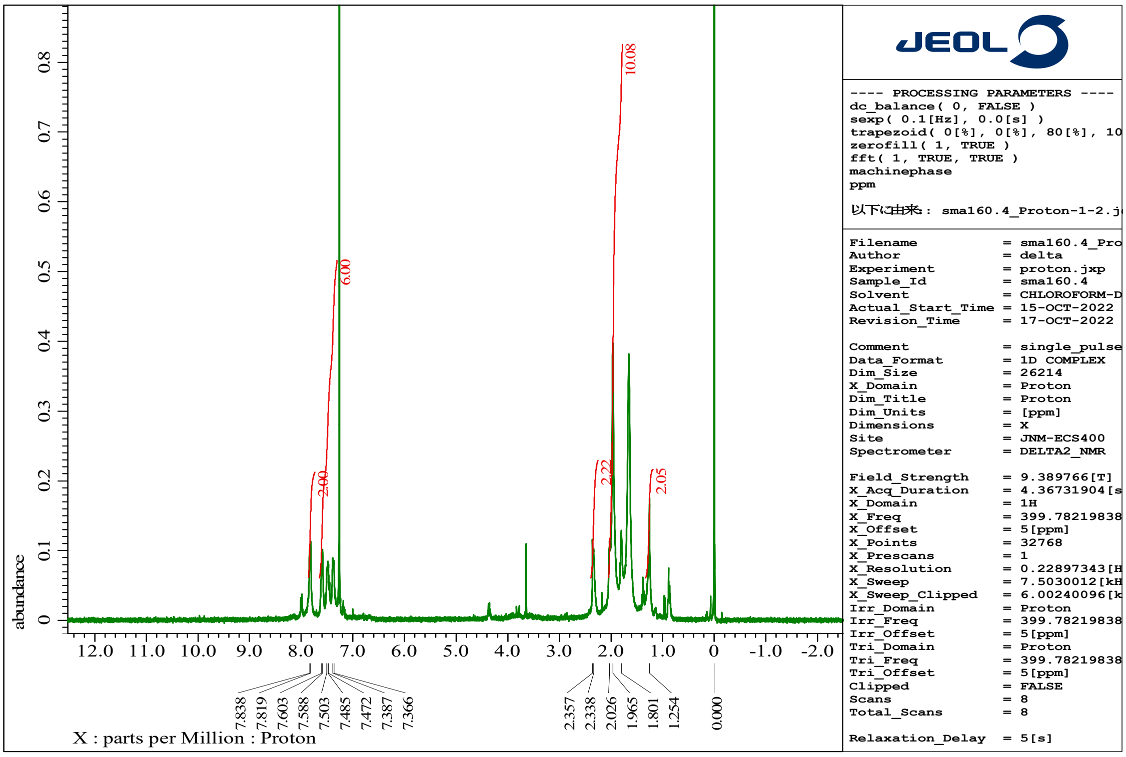
JEOL
---- PROCESSING PARAMETERS ----
dc_balance(0, FALSE)
sexp(0.1[Hz], 0.0[s])
trapezoid(0[%], 0[%], 80[%], 10
zerofill(1, TRUE)
fft(1, TRUE, TRUE)
machinephase
ppm
以下に由来：sma160.4_Proton-1-2.j
Filename = sma160.4_Pro
Author = delta
Experiment = proton.jxp
Sample_Id = sma160.4
Solvent = CHLOROFORM-D
Actual_Start_Time = 15-OCT-2022
Revision_Time = 17-OCT-2022
Comment = single_pulse
Data_Format = 1D COMPLEX
Dim_Size = 26214
X_Domain = Proton
Dim_Title = Proton
Dim_Units = [ppm]
Dimensions = X
Site = JNM-ECS400
Spectrometer = DELTA2_NMR
Field_Strength = 9.389766[T]
X_Acq_Duration = 4.36731904[s
X_Domain = 1H
X_Freq = 399.78219838
X_Offset = 5[ppm]
X_Points = 32768
X_Prescans = 1
X_Resolution = 0.22897343[H
X_Sweep = 7.5030012[kH
X_Sweep_Clipped = 6.00240096[k
Irr_Domain = Proton
Irr_Freq = 399.78219838
Irr_Offset = 5[ppm]
Tri_Domain = Proton
Tri_Freq = 399.78219838
Tri_Offset = 5[ppm]
Clipped = FALSE
Scans = 8
Total_Scans = 8
Relaxation_Delay = 5[s]
abundance
0 0.1 0.2 0.3 0.4 0.5 0.6 0.7 0.8
6.00
2.00
10.08
2.22
2.05
12.0 11.0 10.0 9.0 8.0 7.0 6.0 5.0 4.0 3.0 2.0 1.0 0 -1.0 -2.0
7.838 7.819 7.603 7.588 7.503 7.485 7.472 7.387 7.366
2.357 2.338 2.026 1.965 1.801 1.254
0.000
X : parts per Million : Proton

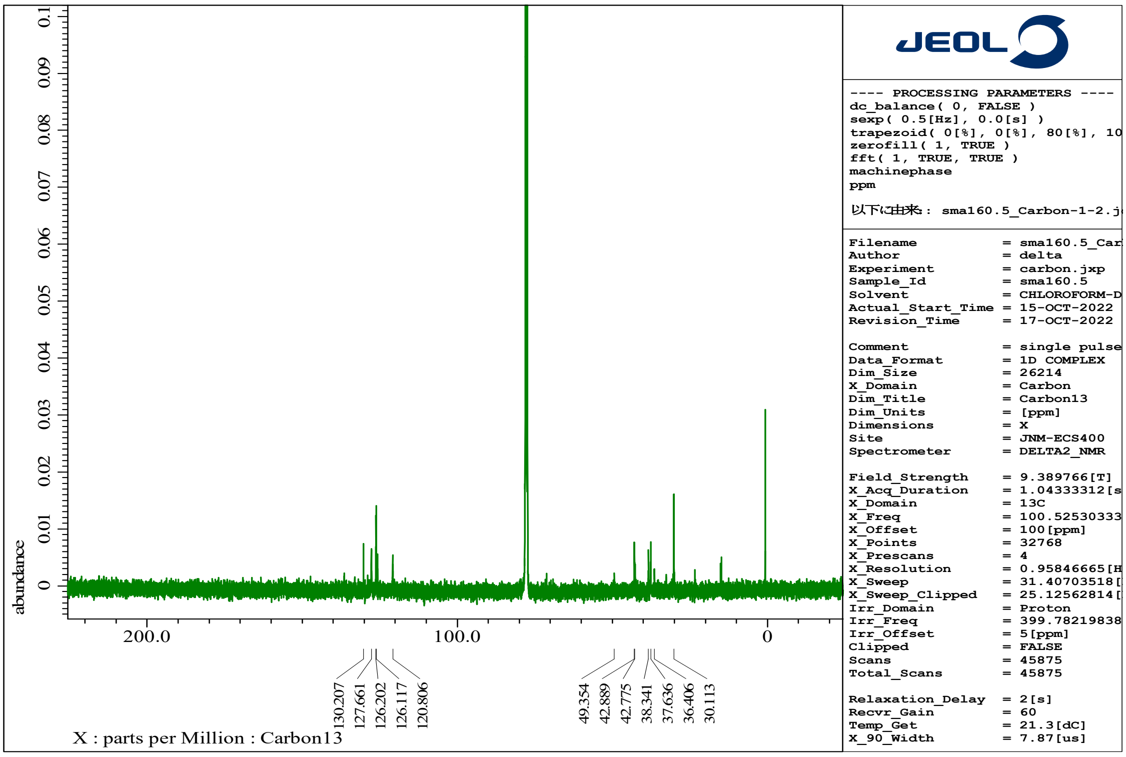
JEOL
---- PROCESSING PARAMETERS ----
dc_balance(0, FALSE)
sexp(0.5[Hz], 0.0[s])
trapezoid(0[%], 0[%], 80[%], 10
zerofill(1, TRUE)
fft(1, TRUE, TRUE)
machinephase
ppm
以下に由来：sma160.5_Carbon-1-2.j
Filename = sma160.5_Car
Author = delta
Experiment = carbon.jxp
Sample_Id = sma160.5
Solvent = CHLOROFORM-D
Actual_Start_Time = 15-OCT-2022
Revision_Time = 17-OCT-2022
Comment = single pulse
Data_Format = 1D COMPLEX
Dim_Size = 26214
X_Domain = Carbon
Dim_Title = Carbon13
Dim_Units = [ppm]
Dimensions = X
Site = JNM-ECS400
Spectrometer = DELTA2_NMR
Field_Strength = 9.389766[T]
X_Acq_Duration = 1.04333312[s
X_Domain = 13C
X_Freq = 100.52530333
X_Offset = 100[ppm]
X_Points = 32768
X_Prescans = 4
X_Resolution = 0.95846665[H
X_Sweep = 31.40703518[
X_Sweep_Clipped = 25.12562814[
Irr_Domain = Proton
Irr_Freq = 399.78219838
Irr_Offset = 5[ppm]
Clipped = FALSE
Scans = 45875
Total_Scans = 45875
Relaxation_Delay = 2[s]
Recvr_Gain = 60
Temp_Get = 21.3[dC]
X_90_Width = 7.87[us]
abundance
0 0.01 0.02 0.03 0.04 0.05 0.06 0.07 0.08 0.09 0.1
200.0 100.0 0
130.207 127.661 126.202 126.117 120.806
49.354 42.889 42.775 38.341 37.636 36.406 30.113
X : parts per Million : Carbon13

S3. References

32. Sheldrick, G.M. SHELXT—Integrated space-group and crystal-structure determination. *Acta Crystallogr.* **2014**, *A70*, C1437.
33. Sheldrick, G.M. Crystal structure refinement with SHELXL. *Acta Crystallogr.* **2015**, *C71*, 3−8.
34. Spek, A.L. PLATON SQUEEZE: A tool for the calculation of the disordered solvent contribution to the calculated structure factors. *Acta Crystallogr.* **2015**, *C71*, 9−18.
